# The complete mitochondrial genome of *Cenchrus fungigraminus* indicates structural dynamics and sequence divergences in Poaceae family

**DOI:** 10.3389/fpls.2025.1589847

**Published:** 2025-05-30

**Authors:** Wentao Sheng

**Affiliations:** ^1^ Department of Biological Technology, Nanchang Normal University, Nanchang, Jiangxi, China; ^2^ Jiangxi Provincial Key Laboratory of Poultry Genetic Improvement, Nanchang Normal University, Nanchang, China

**Keywords:** C*. fungigraminus*, rps2, Ka/Ks, phylogenetic analysis, mitochondria

## Abstract

**Background:**

*Cenchrus fungigraminus* is a perennial grass species, which is used in the cultivation of edible fungi, helping to resolve land use conflicts between edible fungus cultivation and forest production. It can be also utilized for bioethanol production and fiberboard manufacturing. Currently, no report was released on the mitochondrial genome. Thus, the *C. fungigraminus* mitochondrial genome was assembled and annotated utilizing a combination of Illumina and PacBio sequencing data.

**Results:**

The assembled genome measures 404,398 bp and exhibits a linear structure. The GC content is 43. 98%, and 64 characteristic genes were identified, containing 34 protein-coding, six rRNA, 22 tRNA and two pseudo-genes, with 17 genes containing 30 introns. The mitochondrial genome codon usage displayed a significant A/T preference. A total of 235 dispersed, 93 simple sequence, and 38 tandem repeats were detected. Furthermore, 34 sequence fragments (17,634 bp) were transferred from the chloroplast to the mitochondria, accounting for 4.36% of the complete mitochondrial genome. The gene *rps2* was identified as having the highest polymorphism. Additionally, these genes (*ccmB*, *nad2*, and *rps7*) were influenced by positive selection. Among the reported Poaceae mitochondrial genomes, the genome sizes range from 381,065 to 704,100 bp, with GC content ranging from 42.27% to 44.43%. Three genes (*sdh3*, *rps10*, and *rpl6*) are missing in the Poaceae mitochondrial genome, indicating significant rearrangements in the overall genome structure and low homology. It was shown that *C. fungigraminus* is most closely related to *Saccharum officinarum* within the Poaceae family by Phylogenetic analysis. The interspecific Ka/Ks comparison indicated that 28 protein-coding genes have a Ka/Ks ratio < 1.

**Conclusion:**

Therefore, the mitochondrial genome data of *C. fungigraminus* was enriched and a theoretical basis was provided for germplasm evaluation and new variety utilization.

## 1 Introduction

Mitochondria in plants produce energy through aerobic respiration, participating in key metabolic processes that regulate cell differentiation, growth and apoptosis (Wang et al., 2023). They are also closely related to important traits such as plant growth vigor, stress tolerance, and cytoplasmic male sterility (Tan et al., 2022), making them essential tools for research on plant evolution, species identification, genetic diversity, and molecular breeding (Lu et al., 2023a). The mitochondrial genome is characterized by a relatively small size, highly conserved gene composition, uneven distribution, and highly variable non-coding regions (Ma et al., 2022). Compared with the chloroplast genome of plants, the study of its mitochondrial genome is more difficult in organelle genomes, with large intergenic regions, higher assembly and annotation difficulties, and greater structural variation (Wu et al., 2022; Liu et al., 2024). Currently, its research lags far behind that of complete chloroplast genomes. Although nearly 13,000 complete chloroplast genomes were released in NCBI, only 673 entire plant mitochondrial genomes are available (Wang et al., 2024a). Mitochondrial genomes exhibit significant differences in structure, gene content, repeat sequences, and nucleotide substitution rates in different plants (Gualberto et al., 2014; Vothknecht and Szabo, 2020), resulting in complex structures such as circular, branched, reticulated, and their combined forms. Genome lengths vary widely, from 22 kb in *Avicennia marina* to 11.7 Mb in *Larix gmelinii* (Chen et al., 2023). Thus, assembling plant mitochondrial genome is challenging. Consequently, plant systematic studies are mainly focused on nuclear and chloroplast genome sequence, with complete assembly of plant mitochondrial genomes still representing a bottleneck in evolutionary biology. With the advancement of high-throughput sequencing technologies and Bioinformatic methods, software programs suitable for mitochondrial genome assembly and annotation have been developed, such as GetOrganelle (Jin et al., 2020), GSAT (He et al., 2023), Mitofiner (Allio et al., 2020), and PMAT (Bi et al., 2024), making mitochondrial genome research more efficient (Kuang and Yu, 2019), and providing strong technical support for in-depth understanding of species’ genetic characteristics.

*Cenchrus fungigraminus* Z.X. Lin & D.M. Lin & S.R. Lan sp. nov. (JUJUNCAO) is a C4 plant of the genus *Cenchrus* in the Poaceae family, native to tropical regions (Zhou et al., 2020; Song et al., 2023). This species is characterized by strong tillering ability, a well-developed root system, and high biomass yield. Evidence based on morphology, plastid genomes, nuclear genomes, and population genetics further supports *C. fungigraminus* as a new species within the subfamily Panicoideae of the Poaceae family. This plant can grow 5 to 10 meters tall, with branched upper nodes on its stems; the leaves are 1.8 to 4.8 centimeters wide; the inflorescences have 6 to 15 clusters of raceme flowers per centimeter, with many bristles densely covered in long hairs, distinguishing it from all known other species of the genus *Cenchrus* (Lin et al., 2022). Studies indicate that *C. fungigraminus* exhibits tolerance to drought (Zhou et al., 2021), salt stress (Hayat et al., 2020), and heavy metal pollution (Wang et al., 2015), making it a pioneer plant for ecological restoration. Additionally, *C. fungigraminus* has significant economic value due to its high biomass production, serving as a new energy plant (Lu et al., 2015; Ao et al., 2021) and an important forage (Zhao et al., 2020). It has also become a high-quality grass species suitable for China’s climate and soil conditions, though it is sensitive to low-temperature stress (Song et al., 2023) and has limited chilling tolerance (Li et al., 2020). Enhancing the *C. fungigraminus* resistance to abiotic stresses such as cold is crucial for new energy supply, global ecology, and livestock industry. Previous research on *C. fungigraminus* has primarily focused on cultivation techniques and resource conversion applications, including the cultivation of edible fungi (Lei et al., 2019; Lin et al., 2021), environmental restoration, ecological management (Wang et al., 2015), and animal feed development (Zhao et al., 2020), with varying degrees of applied research conducted. It is evident that the foundational scientific research on *C. fungigraminus* remains weak, lacking a systematic theoretical research framework, with significant scientific problems urgently needing resolution, such as the related growth mechanisms behind its large biomass, stress resistance mechanisms, and the innovation of unique germplasm resources for chilling resistance.

Up to now, in the field of *C. fungigraminus* genetics, only reports have been made on its nuclear genome (https://www.ncbi.nlm.nih.gov/datasets/genome/GCA_036936925.1/, GCA_036936925.1) (Zheng et al., 2023) and chloroplast genome (NC_082919.1); no sequences were registered on the *C. fungigraminus* mitochondrial genome, and 19 single nucleotide sequences were retrieved from NCBI (https://www.ncbi.nlm.nih.gov/nuccore/?term=Cenchrus+fungigraminus). Therefore, we utilized Illumina and PacBio sequencing data of *C. fungigraminus* as experimental materials, successfully obtaining its mitochondrial and chloroplast genome. Furthermore,the structural characteristics of the mitochondrial genome were deeply explored, aiming to provide *C. fungigraminus* genome information for its genetic background and evolutionary relationship, and to promote its utilization in genetic improvement work.

## 2 Methods

### 2.1 Data acquisition

Raw Illumina and PacBio sequencing data of *C. fungigraminus* were downloaded from NCBI under bioproject PRJNA882214 (https://www.ncbi.nlm.nih.gov/bioproject/?term=PRJNA882214).

### 2.2 Genome assembly and annotation

The SMRT (V2.3.0) (https://www.pacb.com/) was used to filter low-quality raw long-read sequences and sequencing adapters, resulting in clean long-read (CLR) sequences. Minimap 2 (V2.1) (https://github.com/lh3/minimap2) was used to compare CLR of core mitochondrial genes from *Oryza sativa japonica* (BA000029) (Li, 2021). Sequences longer than 50 bp were chosen as candidate sequences, with those having more alignments and higher quality considered as seed sequences. Then, we assembled mitochondrial genomes using the “autoMito” model using HiFi reads and PMAT v1.5.3 software. The assembly parameter settings are as follows: “- st HiFi-g 250M tp mt ml 40 mi 90” (Bi et al., 2024). The mitochondrial genome size of *C. fungigraminus* was inferred through k-mer analysis of second-generation clean reads. The preliminary assembly sequence obtained was visualized using Bandage v0.8.1 software, and chloroplasts and nuclear contigs were manually removed (Wick et al., 2015). The HiFi reads were used to map the mitochondrial genome of *C. fungygramminus*, in order to identify repetitive regions using the Minimap2 v2.24 tool (Li, 2021) and ultimately generate a mitochondrial contig of *C. fungygramminus*. The chloroplast genome of *C. fungygramminus* was assembled using second-generation sequencing clean reads and Getorganelle v1.7.7 software (Jin et al., 2020).

Blast (https://blast.ncbi.nlm.nih.gov/Blast.cgi) was used to compare *C. fungigraminus* with published plant mitochondrial protein-coding and rRNA gene, followed by manual adjustments based on the reference genome of *Oryza sativa indica* (JF281153) and *Saccharum officinarum* (LC107874). tRNAs were annotated using tRNAscan-SE (http://lowelabucsc.edu/tRNAscan-SE/) (Zou et al., 2015). Open Reading Frame (ORF) Finder (http://www.ncbi.nlm.nih.gov/gorf.html) was used to annotate ORFs, and the software Draw Organelle Genome Maps (https://chlorobox.mpimp-golm.mpg.de/OGDraw.html) was utilized to visualize the mitochondrial genome map (Greiner et al., 2019).

### 2.3 Repeat sequence analysis and editing site prediction

Simple sequence repeat (SSR) analysis of the *C. fungigraminus* mitochondrial genome was conducted using MISA (http://webblast.ipk-gatersleben.de/misa/) (Beier et al., 2017). Tandem Repeats Finder V4.09 (https://tandem.bu.edu/trf/trf.whatnew.html) was made to discriminate tandem repeat sequences longer than 6 bp with a matching degree exceeding 95%, with parameters set to: 27 780 10 50 2000-f-d-m (Benson, 1999). Dispersed repeat sequences were detected using Blastn (V2.10.1), with parameters set to: sequence length of 7, e-value of 1e-5. Visualization of the above repeat sequences was accomplished using Circos V0.69-5 (http://circos.ca/software/download) (Krzywinski et al., 2009). Potential RNA editing sites of *C. fungigraminus* protein coding genes (PCGs) were predicted with PREPACT3 (http://www.prepact.de/prepact-main.php) (Henning et al., 2018) to explore modifications that may affect protein function or regulation.

### 2.4 Homologous sequence analysis of chloroplast genome

The *C. fungigraminus* chloroplast genome was assembled from NCBI (https://www.ncbi.nlm.nih.gov/bioproject/?term=PRJNA882214). Homologous fragments between the mitochondrial and chloroplast genomes were discriminated using BLAST (https://blast.ncbi.nlm.nih.gov/Blast.cgi) available on NCBI, with filtering criteria set to: matching rate ≥70%, E-value ≤1e-5, and length ≥30 bp. The software Circos (V0.69-5) (Krzywinski et al., 2009) was used to visualize the selected sequence fragments.

### 2.5 Codon preference and selection pressure analysis

The mitochondrial codon composition and its relative synonymous codon usage (RSCU) of each species were analyzed using the online bioinformatics cloud platform (http://112.86.217.82:9919/#). These species were included *C. fungigraminus* (PQ720777), *Aegilops* sp*eltoides* (AP013107), *Aegilops longissimi* (KJ078648.1), *Eleusine inadica* (MF616338), *Hordeum vulgare* subsp. *vulgar* (MN127968), *Hordeum vulgare* subsp*. spontaneum* (MN127974), *Lolium perenne* (JX999996), *Oryza coarctata* (MG429050), *Oryza rufipogon* (AP012527), *Oryza minuta* (KU176938), *Oryza sativa* Japonica (BA000029), *Oryza sativa* Indica (JF281153), *Saccharum officinarum* (LC107874), *Sorghum bicolor* subsp. *drummondii* (MZ506736.1), *Sorghum bicolor* (DQ984518), *Triticum aestivum* (AP008982), *Triticum timopheevii* (AP013106), *Tripsacum dactyloides* (NC_008362), *Zea luxurians* (DQ645537), *Zea mays* subsp. *may* (NC_007982), *Zea perennis* (DQ645538), *Zea mays* subsp. *parviglumis* (DQ645539), *Chrysopogon zizanioides* (NC_056367), *Elymus magellanicus* (OQ086977.1), *Agropyron cristatum* (PP503006), and *Thinopyrum obtusiflorum* (OK120846.1). Data from these 27 Poaceae species were used to calculate the nonsynonymous/synonymous substitution values (Ka/Ks) of 32 conserved genes, utilizing R programming language for analysis (Zhang et al., 2006).

### 2.6 Mitochondrial genome synteny and phylogenetic analysis

The Blast Ring Image Generator (BRIG) software (Alikhan et al., 2011) was used for mitochondrial genome similarity. Mitochondrial genome sequences from 27 previously published Poaceae plants and the out-group *Arabidopsis thaliana* (NC_037304.1) were downloaded from NCBI (https://www.ncbi.nlm.nih.gov/). PhyloSuite (V1.2.1) (Zhang et al., 2020) was utilized to discriminate and extract the 32 conserved gene sequences (*rpl16*, *rps1*, *rps12*, *rps13*, *rps2*, *rps3*, *rps4*, *rps7, nad1*, *nad2*, *nad3*, *nad4*, *nad4L*, *nad5*, *nad6*, *nad7*, *nad9*, *atp1*, *atp4*, *atp6*, *atp8*, *atp9*, *ccmB*, *ccmC*, *ccmFc*, *ccmFn*, *cob*, *cox1*, *cox2*, *cox3*, *matR*, and *mttB*) for each species. MAFFT (V7.450) (Katoh et al., 2019) was used to align the conserved gene sequences, and their sequences were concatenated to build a phylogenetic tree. The best model was constructed using ModelFind, and maximum likelihood (ML) analysis with 1,000 bootstrap replications was carried out in RaxML (V8.2.4) (Höhler et al., 2022).

## 3 Results

### 3.1 Characteristics of the *C. fungigraminus* mitochondrial genome

**Figure 1:**
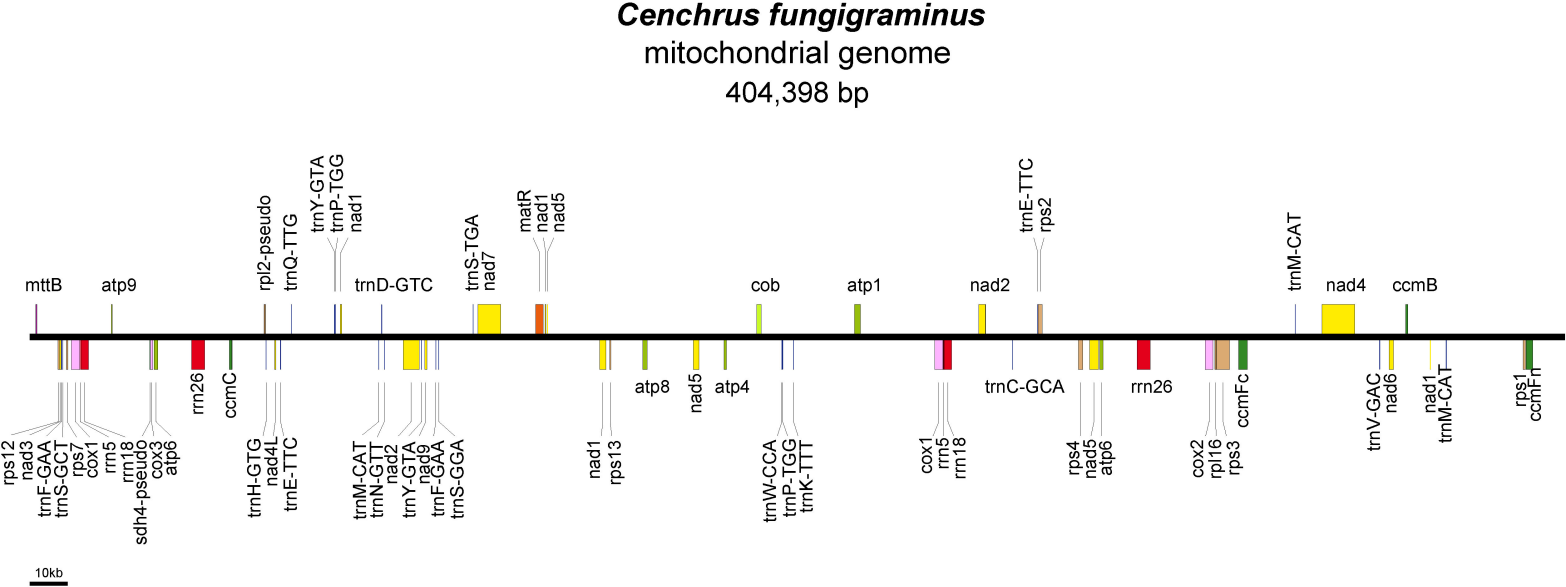
Mitochondrial genome of *Cenchrus fungigraminus.*.

We obtained a complete mitochondrial genome structure through *de novo* assembly (**Supplementary Figure S1A**: gfa diagram). Based on connection relationships and sequence depth, this genome is a non-circular complex structure composed of 9 contigs, with contig5 and contig9 capable of recombination. Unraveling it yielded a linear genome of 404,398 bp (**Supplementary Figure S1B**, **Figure 1**). The assembly result has been uploaded to NCBI with the sequence accession number PQ720776. The nucleotide composition of the *C. fungigraminus* mitochondrial genome is as follows: A=27.94%, T=28.08%, G=21.71%, C=22.27%, with a GC content of 43.98%. A total of 64 genes were annotated, containing 34 PCGs, 6 rRNA genes, 22 tRNA genes, and 2 pseudogenes (*rpl2* and *sdh4*). The total PCGs length is 35,124 bp, with 43.02% GC content; the total tRNA length is 1658 bp, with 51.03% GC content; the total rRNA length is 11,118 bp, with 52.96% GC content. Furthermore, eight genes (*atp6*, *cox1*, *rrn18*, *rrn26*, *rrn5*, *trnE-TTC*, *trnY-GTA*, and *trnP-TGG*) contain two copies, *trnM-CAT* has three copies, *ccmFc*, *cox2*, *rps3*, and *trnF-GAA* contain one intron, *nad4* has three introns, and *nad1*, *nad2*, *nad5*, and *nad7* include four introns (**Supplementary Table S1**).

The *C. fungigraminus* mitochondrial genome includes 11 types of genes, which consist of six ATP synthases, four Cytochrome c biogenesis, one Ubiquinol cytochrome c reductase, four Cytochrome c oxidase, one Maturase, one Transport membrane protein, nine NADH dehydrogenase, one Large ribosomal protein, seven Small ribosomal protein genes, six Ribosomal RNA, and 22 Transfer RNA genes, with no Succinate dehydrogenase genes. The PCGs lengths range from 348 to 2229 bp, with *cox1* being the longest, encoding 743 amino acids, and *mttB* being the shortest, encoding 116 amino acids. The rRNA gene length range from 351 to 1671 bp, and the tRNA gene length range from 70 to 88 bp (**Supplementary Table S2**).

### 3.2 Repeat sequence analysis of the *C. fungigraminus* mitochondrial genome

**Figure 2:**
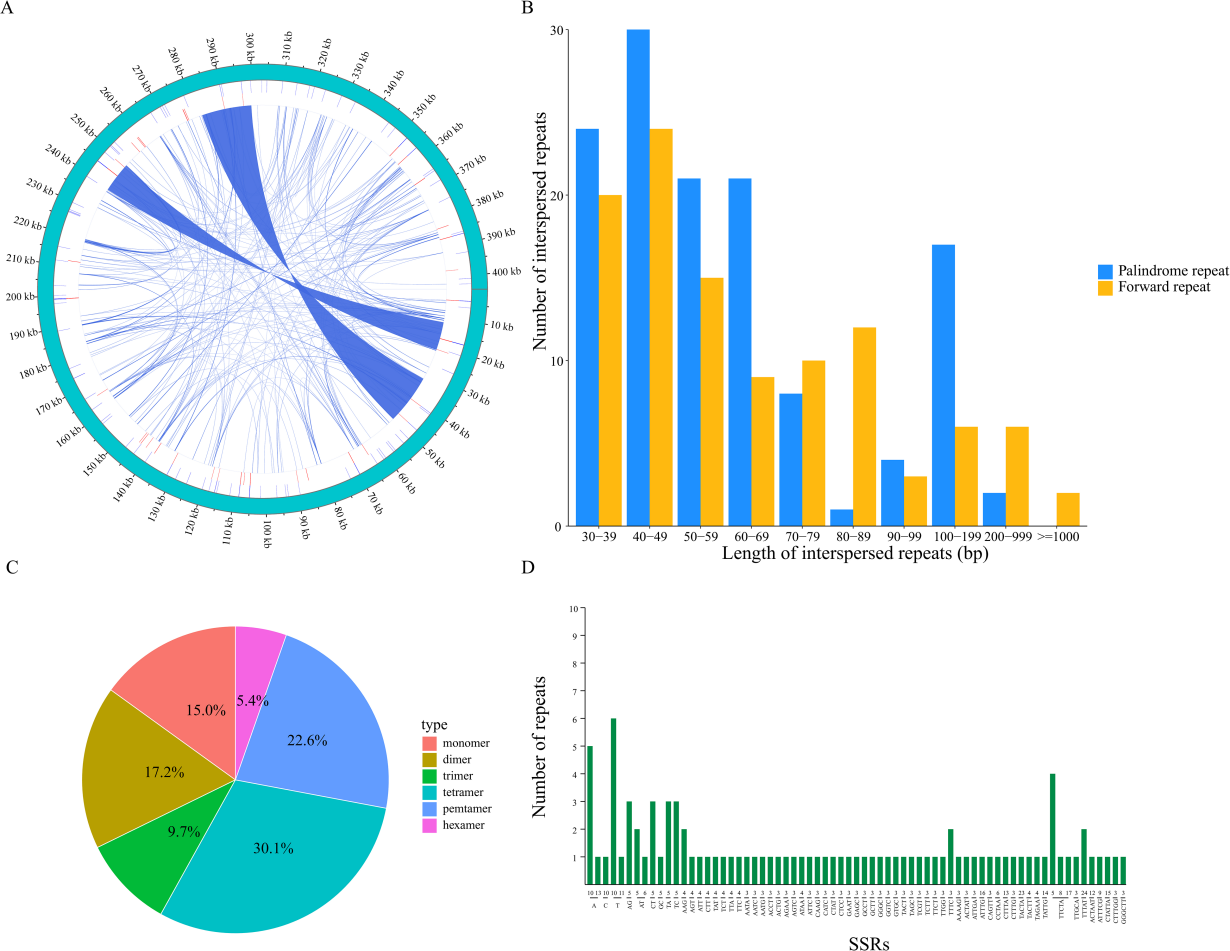
Mitochondrial genomic repeat sequences of *Cenchrus fungigraminus.* **(A)** The distribution of repetitive sequences; **(B)** Length distribution of dispersed repeat; **(C)** SSR type analysis; **(D)** Statistics of repeated sequences with different lengths.

In the *C. fungigraminus* mitochondrial genome, 235 dispersed repeat sequences of lengths ≥30 bp were checked, comprising two types: forward and palindromic dispersed repeats. No forward or complementary repeats were identified, and these dispersed repeats were widely distributed in the intergenic regions. Visual representation was achieved using the Circos software package (**Figure 2A**), with a length of 235 dispersed repetitive sequences amounting to 44,437 bp, which accounts for 10.99% of the complete genome length. Meanwhile, 128 forward repeats and 107 palindromic repeats were discriminated, with the most common repeats being those of 40–49 bp in length, totaling 54 (**Figure 2B** and **Supplementary Table S3**). In total, 93 SSRs were checked in the mitochondrial genome, with tetranucleotide repeats being predominant, comprising 35% (28) of the total, followed by pentanucleotide (21), dinucleotide (16), mononucleotide (14), trinucleotide (9), and hexanucleotide repeats (5). In the mononucleotide SSRs, base A repeat was the most common (92.85%); in the dinucleotide repeats, the highest proportion was found in AG and CT bases, both at 37.5% (**Figures 2C, D**; **Supplementary Table S4**). Tandem repeats are characterized by a repeat length of 1–200 base pairs with varying repeat counts. Additionally, 38 tandem repeat sequences were identified ranging in length from 26 to 120 bp, all located in the intergenic regions (**Supplementary Table S5**).

### 3.3 Prediction of *C. fungigraminus* RNA editing

**Figure 3:**
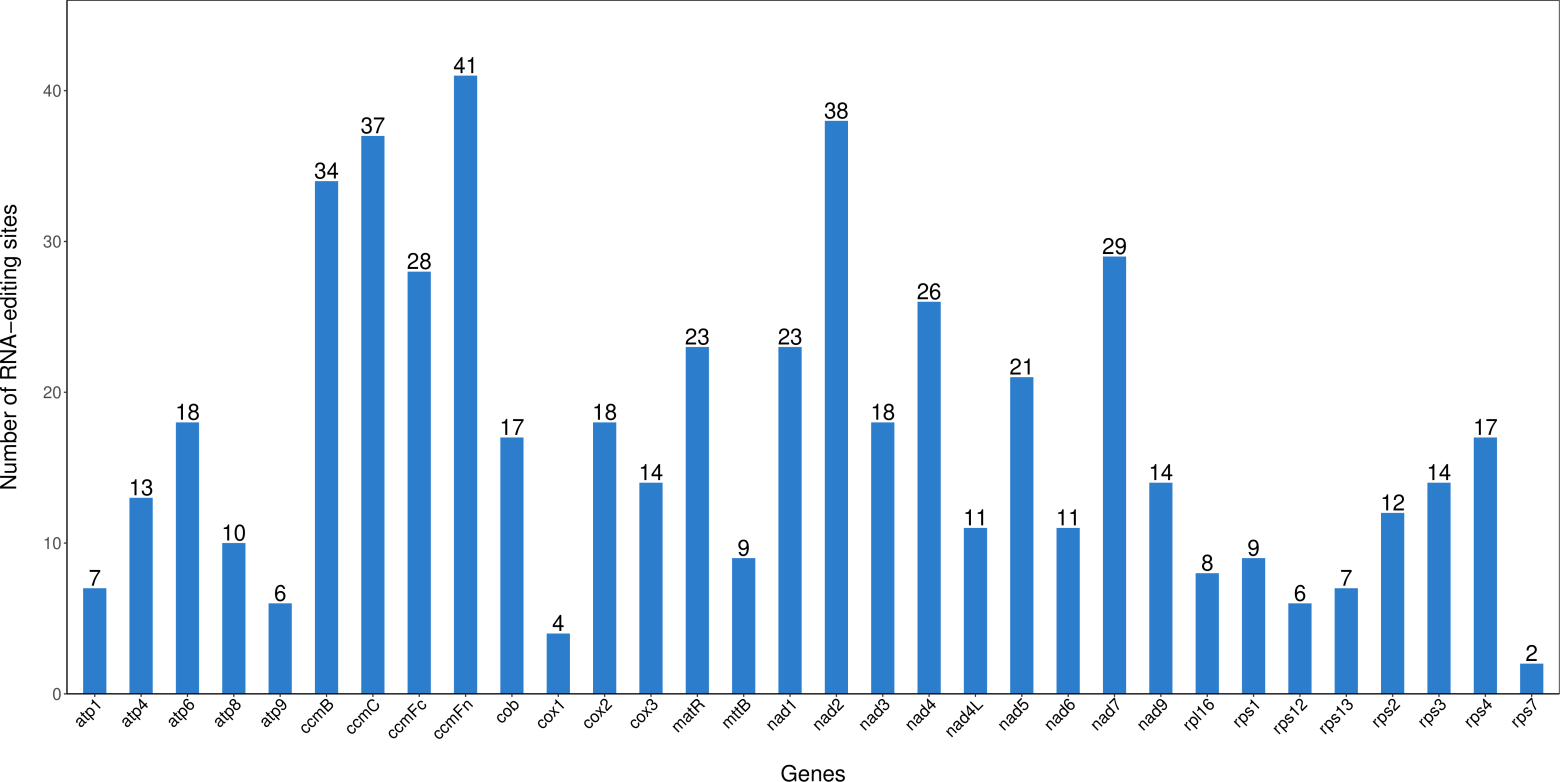
Prediction of *Cenchrus fungigraminus* RNA editing sites.

RNA editing sites were predicted in all PCGs of its genome, revealing characteristics of post-transcriptional modifications. A total of 545 RNA editing events were identified (**Figure 3**), primarily showing C to T conversions, corresponding to C to U transitions in RNA. The *ccmFn* gene owned the most significant editing sites (41), followed by *nad2* with 38 editing sites, while *rps7* had the fewest with 2 RNA editing sites. The study further observed that editing events concentrated on the first and second base positions of its codon. These RNA editing events resulted in amino acid inter-changes, such as threonine (T) to isoleucine (I), leucine (L) to phenylalanine (F), serine (S) to phenylalanine (F), arginine (R) to cysteine (C), histidine (H) to tyrosine (Y), and arginine (R) to tryptophan (W). Among these RNA editing site types, the conversion number of hydrophilic to hydrophobic amino acid was 264, accounting for 48.44%; the conversion number of hydrophobic to hydrophobic amino acid was 177, accounting for 32.48%; and the conversion number of hydrophobic to hydrophilic amino acid was 45, accounting for 8.26% (**Supplementary Table S6**).

### 3.4 Homologous analysis of the *C. fungigraminus* mitochondrial and chloroplast genome

**Figure 4:**
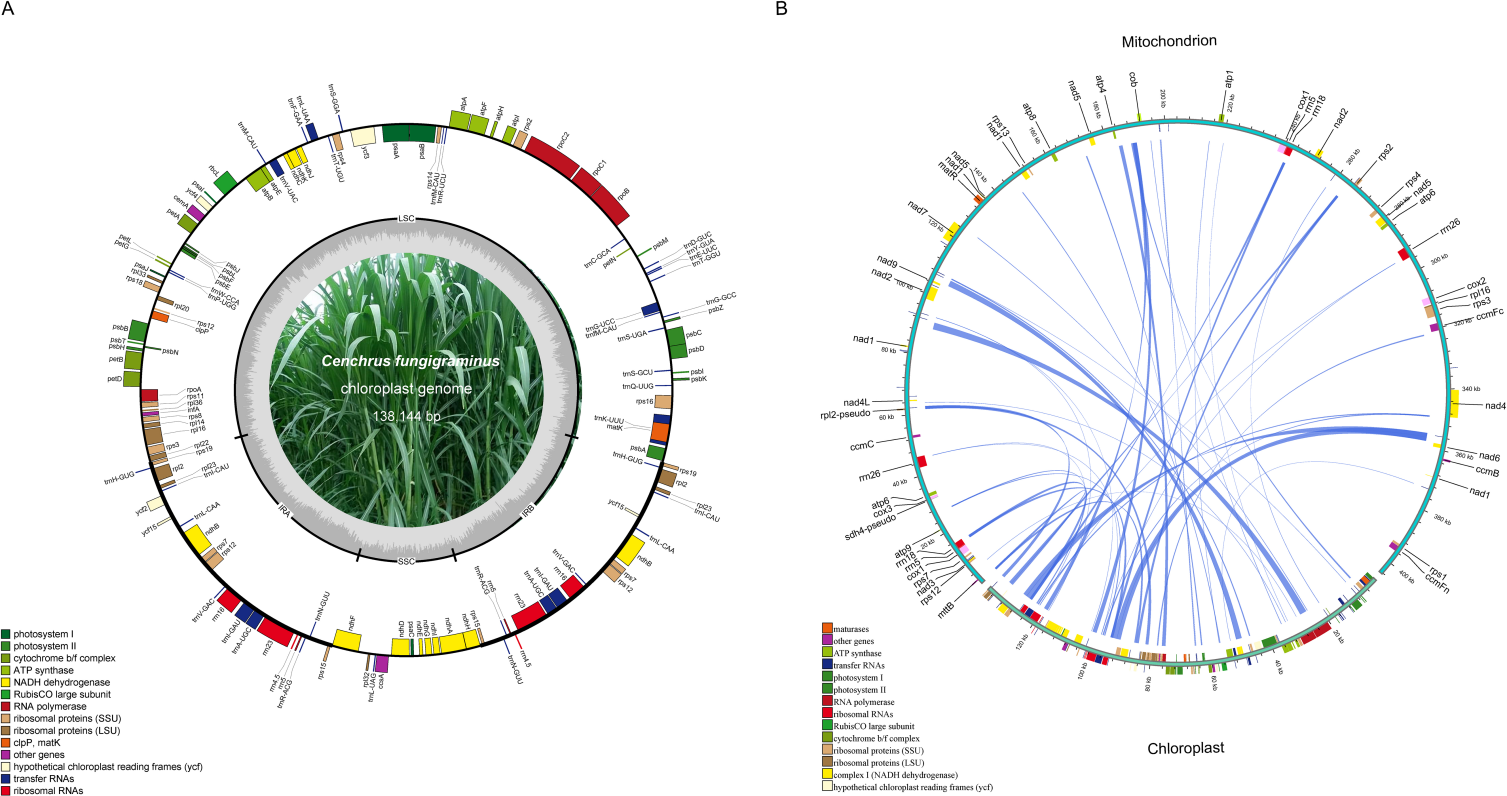
Fragments transferred from chloroplasts to mitochondria in the *Cenchrus fungigraminus* genome. **(A)** The *Cenchrus fungigraminus* chloroplast genome; **(B)** Sequence of *Cenchrus fungigraminus* chloroplast genome transfer to the mitochondrial genome.

The *C. fungigraminus* mitochondrial genome size is approximately 2.93 times that of the chloroplast genome (PQ720777, 138,144 bp). Comparing to the *C. fungigraminus* chloroplast genome, its mitochondrial gene distribution is relatively sparse (**Figure 4A**). Based on the sequence similarity, 34 chloroplast gene fragments were identified to transfer into the mitochondrial genome, with a 17,634 bp length, accounting for 4.36% of its mitochondrial genome. Fifteen genes including *rrn4.5*, *rrn5*, *trnV-GAC*, *trnF-GAA*, *ndhJ*, *trnH-GUG*, *ycf15*, *psbE*, *trnS-GGA*, *trnN-GUU*, *trnP-UGG*, *trnC-GCA*, *trnW-CCA*, *trnS-GCU*, and *trnM-CAU*, were completely transferred. And nineteen genes including *rpoC1*, *rpoC2*, *rps12*, *rpoA*, *rps11*, *rps19*, *rpl2*, *ycf2*, *rrn23*, *trnA-UGC*, *rps16*, *psbF*, *trnA-UGC*, *rrn16*, *rpl14*, *atpF*, *rbcL*, *ndhK*, and *atpI*, underwent partial transfer (**Figure 4B**, **Supplementary Table S7**). The longest transferred fragment was 2287 bp, containing parts of *rpoC1* and *rpoC2*, while the shortest transferred fragment was 42 bp, which was a part of *atpI* (**Supplementary Table S7**).

### 3.5 Codon preference of the *C. fungigraminus* mitochondrial genome

**Figure 5:**
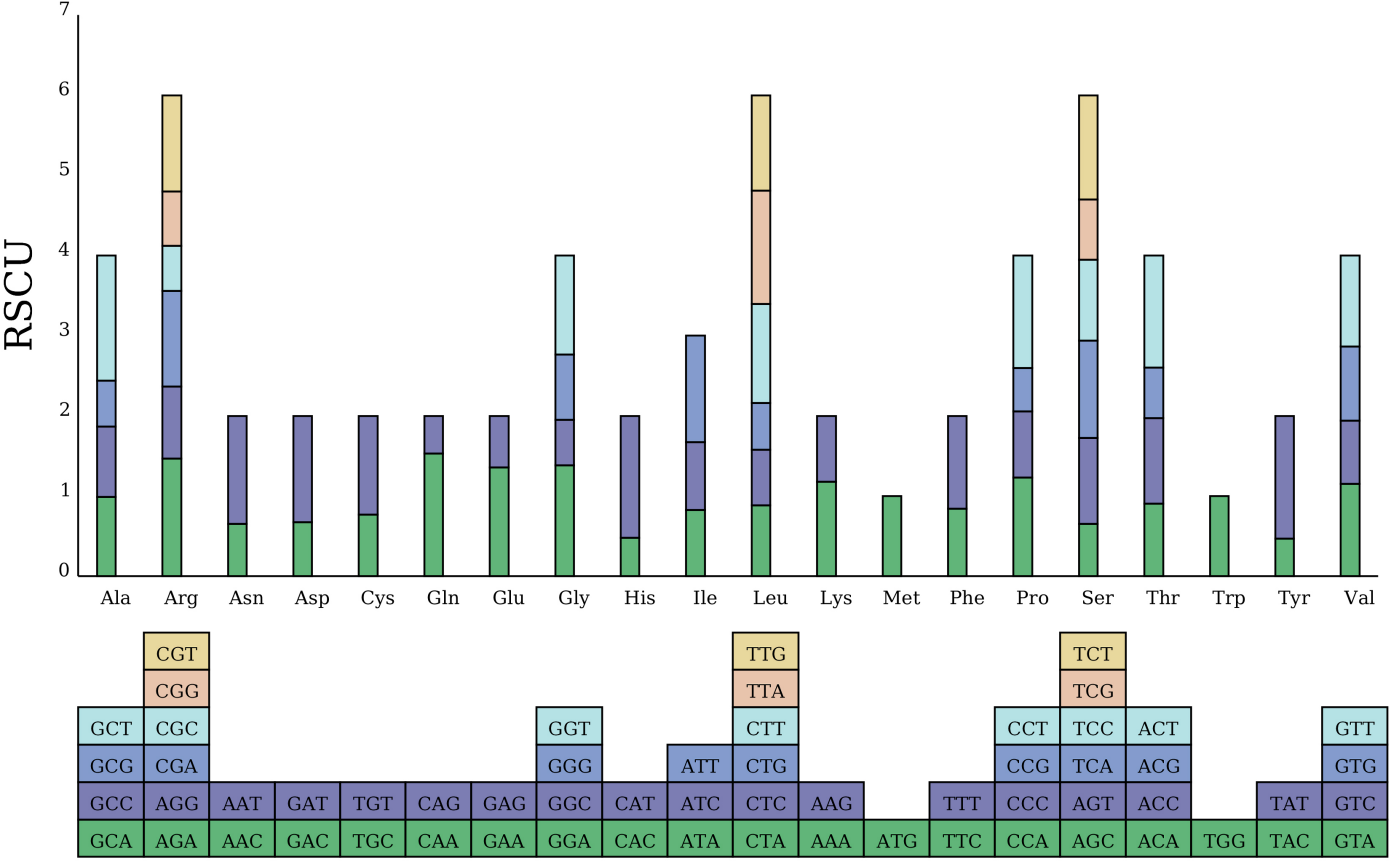
Analysis of RSCU value in the *Cenchrus fungigraminus* mitochondrial genome.

The total length of the 34 PCGs in the *C. fungigraminus* mitochondrial genome is 35,124 bp, containing 10,664 codons. All PCGs select ATG as the start codon, while *nad1* and *nad4L* also choose ACG as the start codon. There are three stop codon types: TAG, TGA, and TAA, with usage frequencies of 32.35%, 20.58%, and 47.06%, respectively (**Supplementary Table S8**). RSCU value can eliminate the amino acid composition influence on codon usage, directly reflecting codon usage pattern difference. An RSCU value equal to one shows no bias in codon usage; RSCU values greater than one indicates higher usage frequency; and RSCU values less than one displays lower usage frequency (Quax et al., 2015). Analysis using the RSCU method revealed that the average RSCU value for all codons was 0.999; the codon GCU coding for alanine (Ala) had the highest frequency, with an RSCU value equal to 1.5589; while the codon CAG coding for glutamine had the lowest frequency, with an RSCU value of 0.4671. There were 31 codons with RSCU > 1, most of which ended in A or U; there were two codons with RSCU = 1, namely methionine (Met) and tryptophan (Trp) (**Figure 5**).

### 3.6 Gene composition in the Poaceae mitochondrial genome

**Figure 6:**
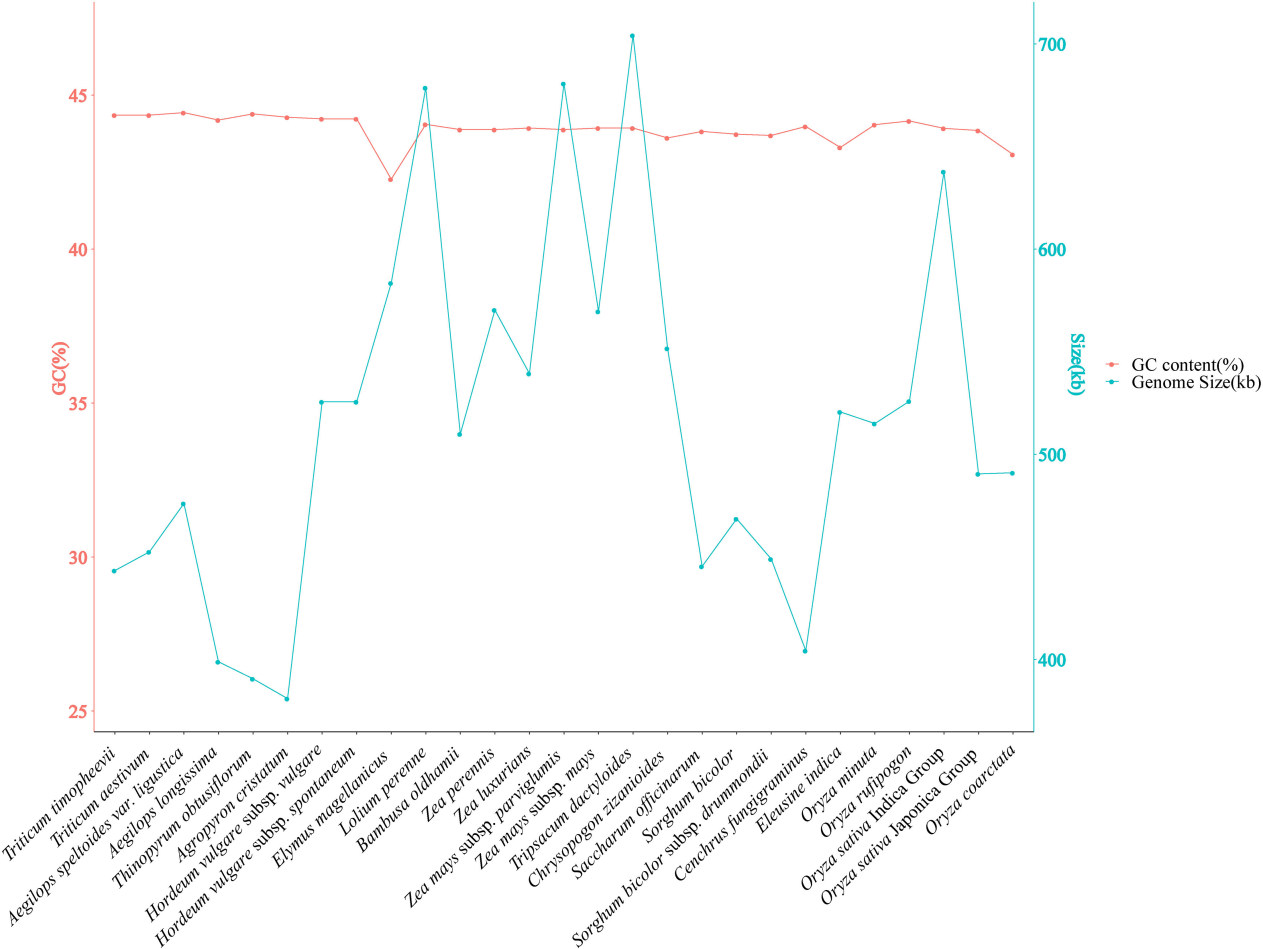
Changes in mitochondrial genome GC content and genome size of 27 species in Poaceae.

The mitochondrial gene content and genome size were compared among 27 Poaceae species. A variation in genome size of two-fold difference was observed, ranging from 381,065 bp in *Agropyron cristatum* to 704,100 bp in *Tripsacum dactyloides*. The GC content of the 27 Poaceae mitochondrial genomes ranged from 42.27% in *Elymus magellanicus* to 44.43% in *Aegilops* sp*eltoides* var. ligustica (**Figure 6**, **Supplementary Table S9**).

**Figure 7:**
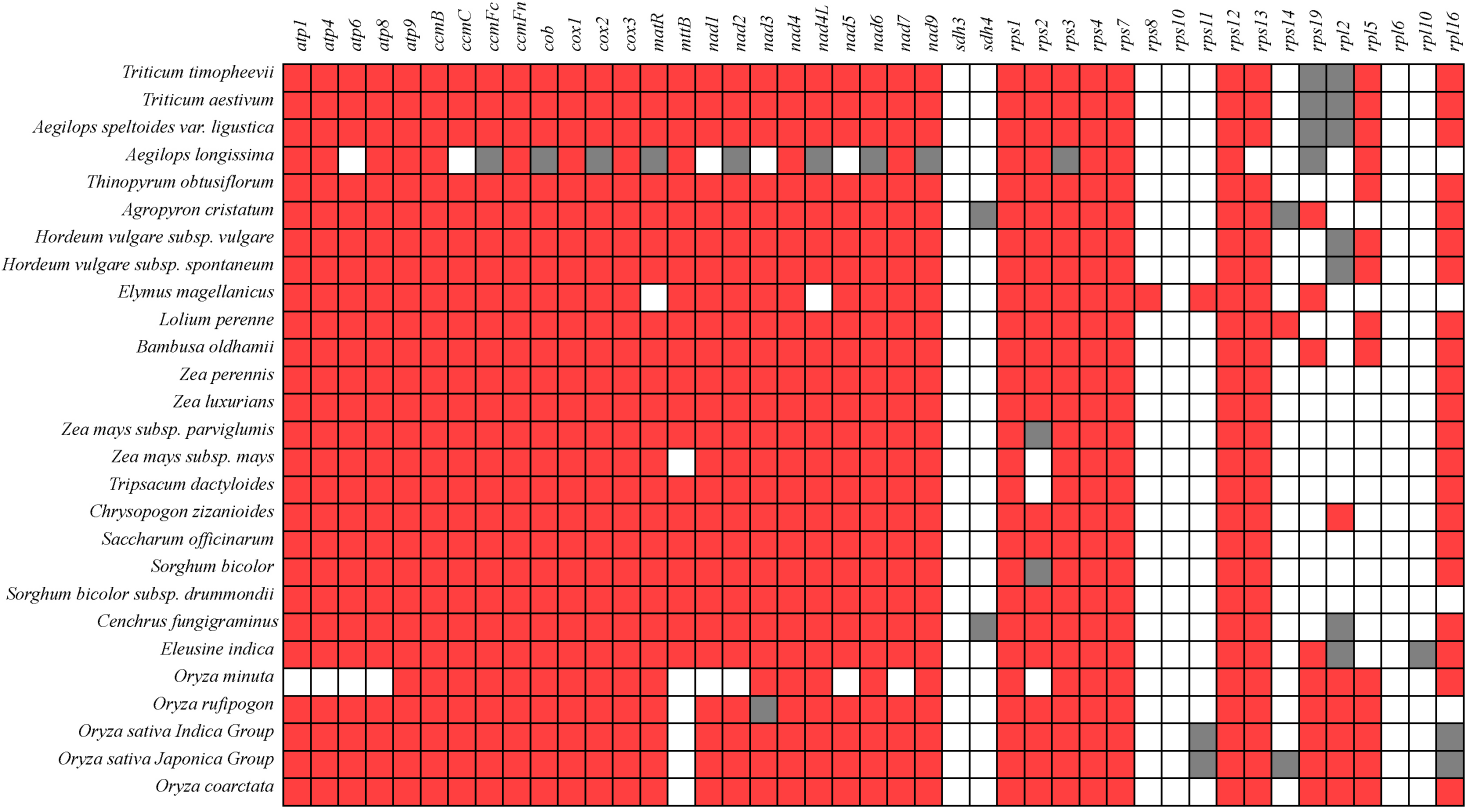
Composition changes of mitochondrial genome genes in 27 species of Poaceae plants (Red indicates existence; White indicates missing; Gray represents pseudogenes).

The mitochondrial genomes exhibit significant differences in size and gene composition. Most PCGs are conserved across different plant mitochondrial genomes, particularly those in groups such as complex I (III, and V), cytochrome c biogenesis, maturation enzymes, and transport membrane proteins (**Figure 7**). The gene conservativeness indicates their critical role in mitochondrial function. Among these genes, *atp9*, *ccmB*, *ccmFn*, *cox1*, *cox3*, *nad4*, *nad9*, *rps1*, *rps4*, *rps7*, and *rps12* are completely present in all genomes. These genes *sdh3*, *rps10*, and *rpl6* are completely absent across all genomes. Genes such as *atp*, *ccm*, *cob*, *cox*, *matR*, *mttB*, *nad*, *sdh*, *rps*, and *rpl* gene exhibit gene loss phenomena among different species, indicating high polymorphism in succinate dehydrogenase and ribosomal protein genes. Notably, *sdh3*, *rps8*, *rps10*, *rps11*, *rps14*, *rps19*, *rpl5*, *rpl6*, and *rpl10* are absent, while *sdh4* and *rpl2* are identified as pseudo-genes in *C. fungigraminus* (**Figure 7**, **Supplementary Table S10**).

### 3.7 Selection pressure analysis in *C. fungigraminus*

**Figure 8:**
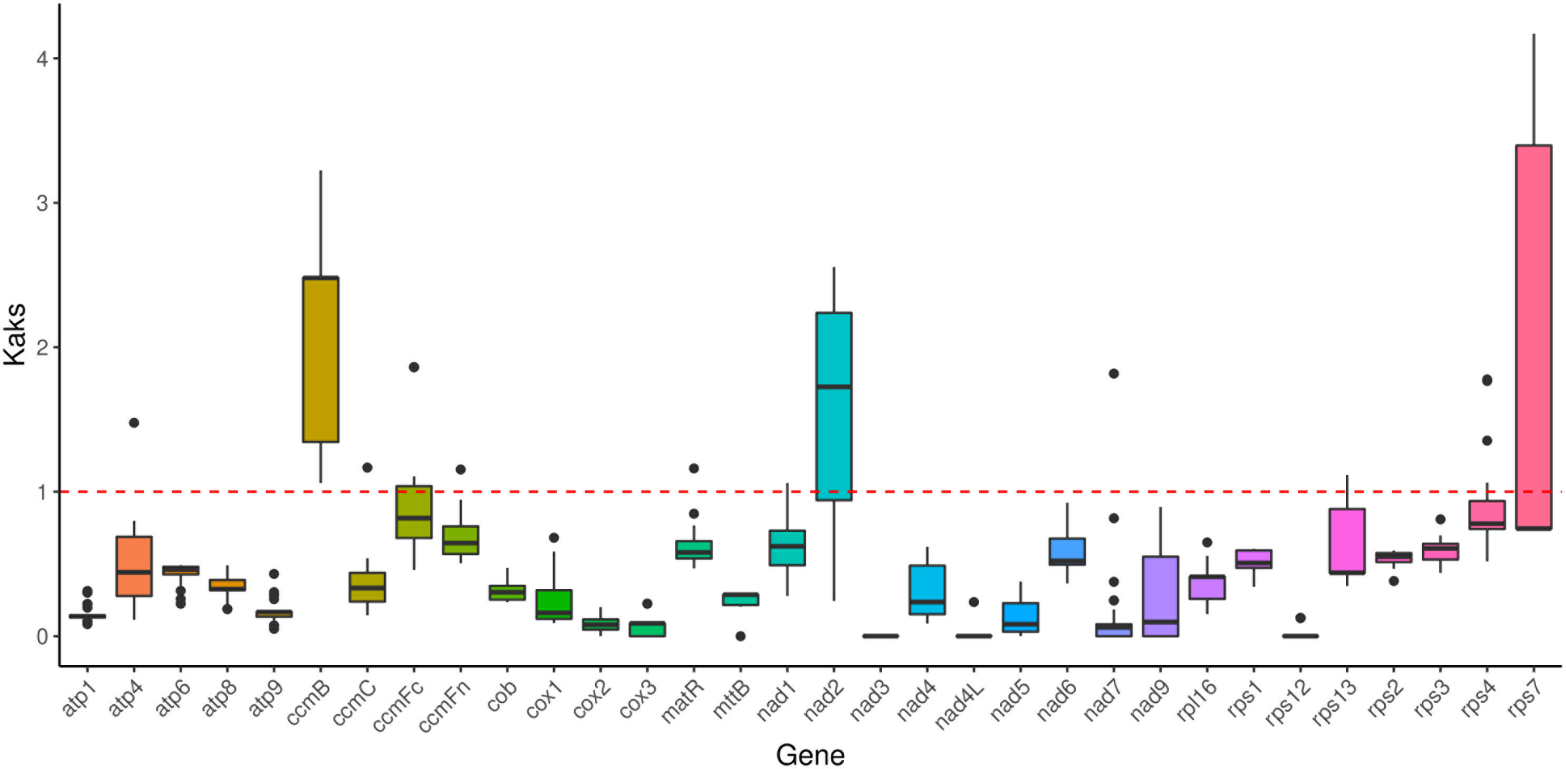
Boxplots of the Ka/Ks ratio in *Cenchrus fungigraminus* and 26 other plant species.

Selection pressure analysis among 27 Poaceae species revealed an average Ka/Ks value of 0.5235 for 32 homologous proteins, indicating that most genes exhibit negative selection effects, and showing that the majority of PCGs in the *C. fungigraminus* mitochondrial genome are highly conserved in evolution. Notably, the Ka/Ks values for *ccmB*, *nad2*, and *rps7* are >1, exhibiting that the three genes may have been subject to positive selection (**Figure 8**, **Supplementary Table S11**).

### 3.8 Identification of highly variable regions

**Figure 9:**
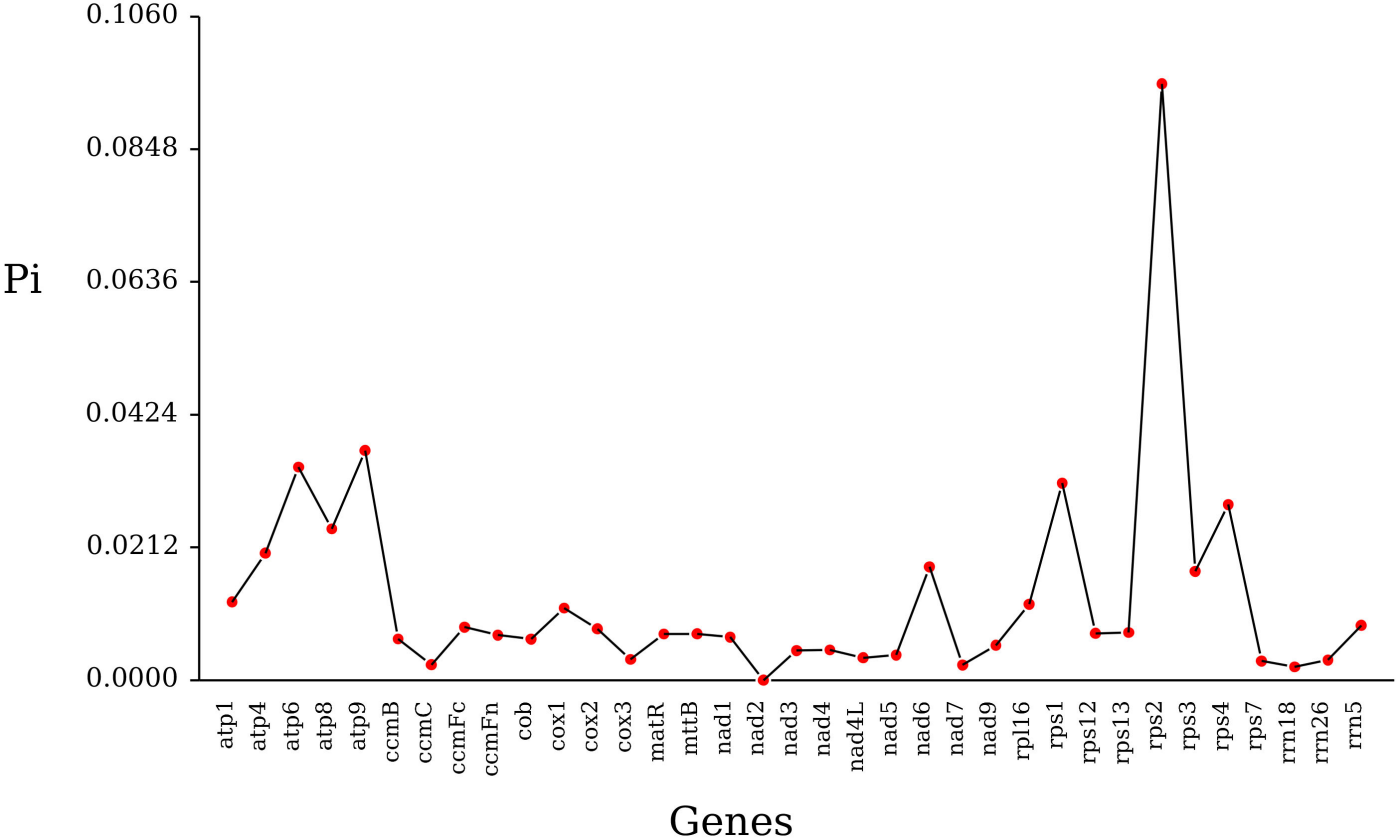
Gene polymorphism sites analysis in the *Cenchrus fungigraminus* mitochondrial genome.

Using DnaSP.6 software, nucleotide polymorphism (Pi) analysis was performed on mitochondrial genes from 27 Poaceae species. A total of 1300 polymorphic sites were detected, with *rps2* and *rps3* having the most polymorphic sites at 194 and 107, while *rrn5* and *nad4L* had the fewest, with three and five polymorphic sites (**Supplementary Table S12**). The Pi values ranged from 0.00212 to 0.09525, with *rrn18* being the smallest and *rps2* having the largest value. The analysis indicates that *rps2* has the highest polymorphism and can serve as a candidate gene locus for population genetics (**Figure 9**).

### 3.9 Collinearity relationships of mitochondrial genomes in Poaceae

**Figure 10:**
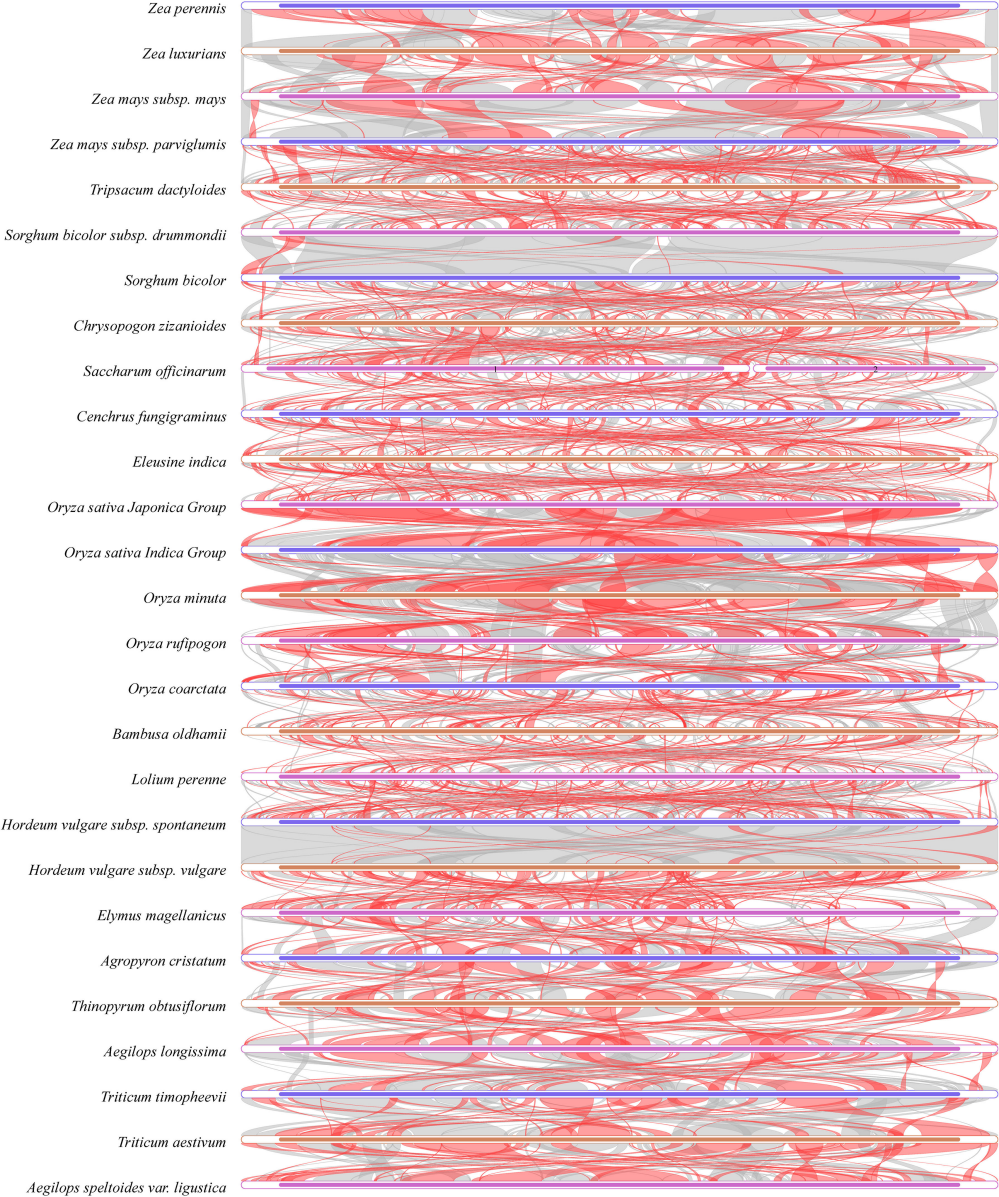
Collinearity analysis of *Cenchrus fungigraminus* with 26 other species sequences in the Poaceae family. Red indicates inversion, while gray indicates good homology.

Gene collinearity includes the arrangement order of gene regions and intergenic regions. Using MUMmer 4.0.0beta2 (https://github.com/topics/mummer) with the –maxmatch parameter, comparisons were made between *C. fungigraminus* (PQ720776) and other Poaceae sequences. Low collinearity was indicated among the Poaceae mitochondrial genome structures, with considerable structural variation; within the same genus, the similarity is relatively high, while there is significant structural variation and low similarity among different genera (**Figure 10**, **Supplementary Table S13**). The *C. fungigraminus* mitochondrial genome shows significant differences from other species, with only 53.72% similarity to *Eleusine indica*, along with notable structural inversions and rearrangements.

### 3.10 Homologous sequences in mitochondria of Poaceae plants

**Figure 11:**
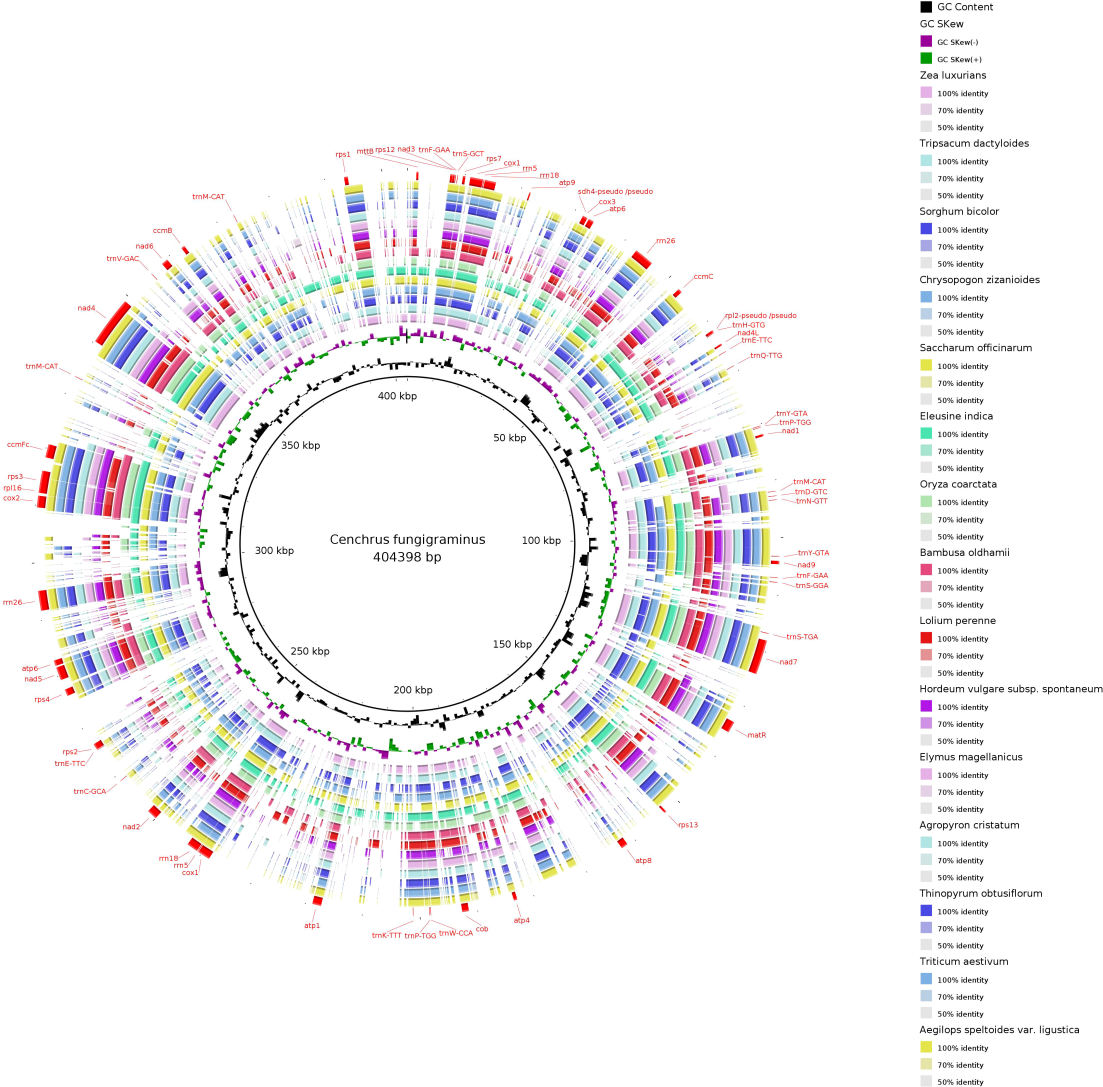
Mitochondrial genome sequence similarity comparison ring map of 15 genera in the Poaceae family.

The *C. fungigraminus* mitochondrial genome sequence was selected as a reference, and BRIG-0.95 software (http://mirrors.sohu.com/ubuntu/pool/universe/b/brig/) was utilized to conduct sequence similarity research of the 27 plant mitochondrial genomes across 15 genera of Poaceae. The results displayed a circular plot with overall poor continuity, indicating varying degrees of differences among species (**Figure 11**). The strength of differences between species is significantly correlated with their phylogenetic relationships, with closely related genera or species forming high similarity circular plots.

### 3.11 Phylogenetic relationships of Poaceae mitochondrial genomes

**Figure 12:**
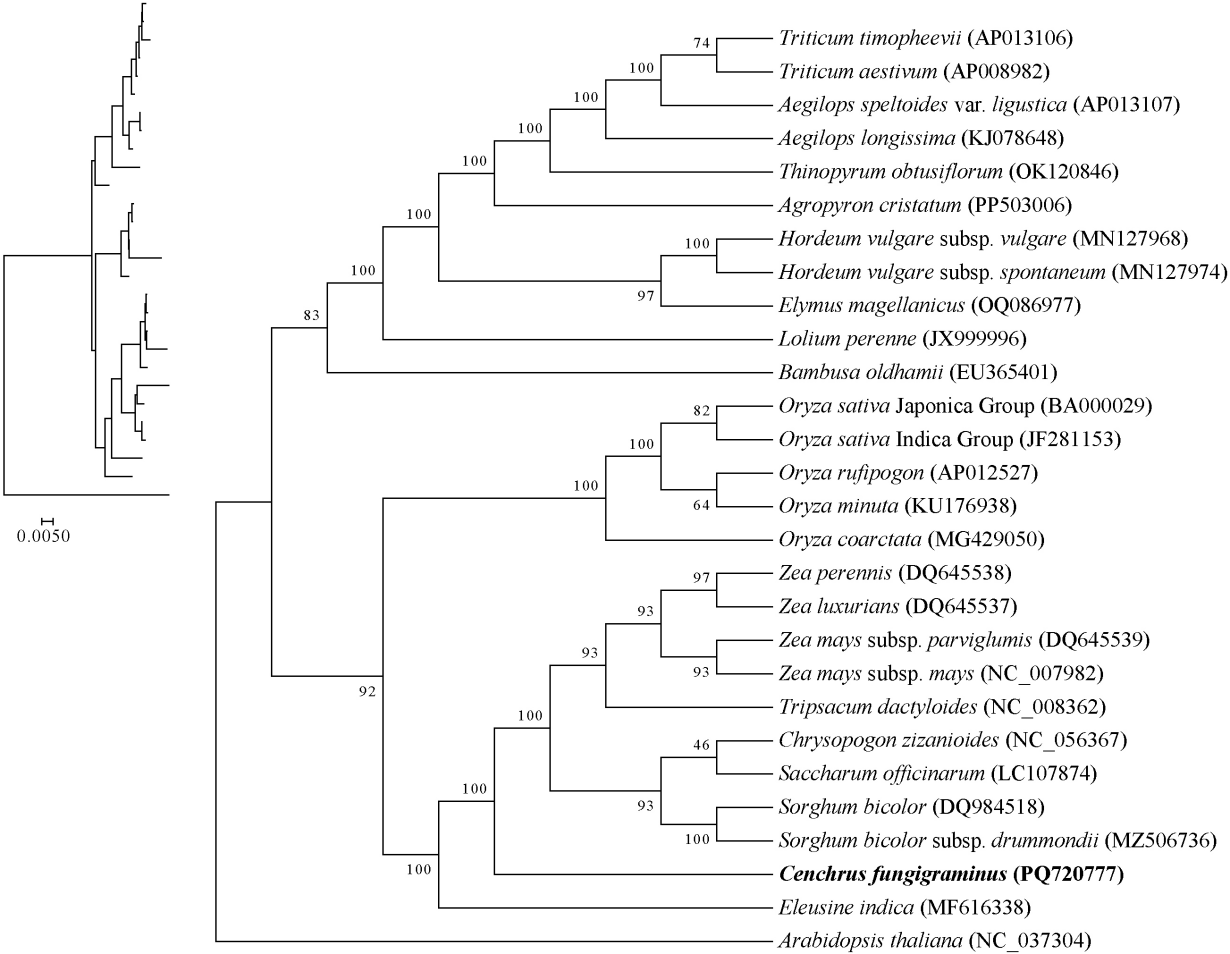
The phylogenetic tree of 27 Poaceae mitochondrion based on 32 protein coding genes.

Based on 32 conserved PCGs in the mitochondria, a phylogenetic tree was constructed that divided the 27 Poaceae species into 3 categories. *Aegilops* sp*eltoides* clustered with *Triticum aestivum* and *Triticum timopheevii*, while simultaneously clustering with *Hordeum vulgare* subsp. *spontaneum* and *Hordeum vulgare* subsp. *vulgar*, and *Lolium perenne*, indicating closer relationships among these species than with others (**Figure 12**). Within the genus *Oryza*, *Oryza rufipogon* and *Oryza sativa*Indica cluster together, followed by *Oryza minuta*, *Oryza coarctata* and *Oryza sativa* Japonica, indicating decreasing phylogenetic relationships, while also clustering with *Bambusa oldhamii*, suggesting a certain phylogenetic relationship between *Bambusa oldhamii* and several *Oryza* species. The genus *Zea*, including *Zea luxurians*, *Zea perennis*, *Zea mays* subsp. *parviglumis* and *Zea mays* subsp. *may*, clusters as one evolutionary branch, subsequently clustering with *Tripsacum dactyloides*, *Saccharum officinarum*, *Sorghum bicolor*, *Chrysopogon zizanioides*, *Eleusine inadica* and *C. fungigraminus* as a large evolutionary branch, indicating close phylogenetic relationships among these genera, with *C. fungigraminus* and *S. officinarum* being the closest. The above results are similar to the CDS gene sequence analysis of chloroplast genome (**Supplementary Figure S2**), indicating that mitochondrial genome can effectively carry out phylogenetic analysis of Poaceae plants.

## 4 Discussion

### 4.1 Genome structure, size, and gene composition

Plant mitochondrial genomes display complex combinations, diverse genome structures, fluctuating intergenic region, and high recombination rates of repetitive sequences, which complicate plant mitochondrial genome studies (Wu et al., 2022). The size reported in NCBI typically ranges from 200 kb to 3 Mb in plant mitochondrial genomes, exhibiting rich structural composition types. Plant mitochondrial DNA structure has been observed, consisting of master circular molecules, sub-genomic circular molecules, linear molecules, and highly branched multi-genomic structures (Mower et al., 2012; Li et al., 2025). For example, the *Salvia miltiorrhiza* and *Salvia officinalis* mitochondrial genomes resulted in two-unit graphs corresponding to branched multi-genomic structures with seven and three bifurcations, respectively (Yang et al., 2022, 2023); the *Punica granatum* mitochondrial genome was identified as a linear structure, measuring 404,807 bp (Lu et al., 2023b). Based on third-generation sequencing, long sequencing fragments can be read (Zhang et al., 2021; Liu et al., 2024). Therefore, it is believed that the mitochondrial genome of the linear *C. fungigraminus* has been successfully assembled with Illumina and PacBio raw data. At the same time, the mitochondrial genome we assembled had uniform coverage, with an average sequencing depth of 223.6× (**Supplementary Figure S3** and **Figure 4**).

In this research, 64 genes were found in *C. fungigraminus* mitochondrial genome, including 34 PCGs, 22 tRNA, 6 rRNA, and 2 pseudo-genes, with gene types and numbers consistent with those of most land plants’ circular mitochondrial genomes (Petersen et al., 2020). The length of PCGs is 35,124 bp, accounting for 8.68% of its genome, similar to that of the *Primulina hunanensis* linear genome, which has a PCG proportion of 9.02% (Chen et al., 2024). The *C. fungigraminus* mitochondrial genome mainly composed of non-coding regions, which may be attributed to the gradual increase of repetitive sequences (Nardi et al., 2012). Several genes, such as *atp6*, *cox1*, *rrn18*, *rrn26*, *rrn5*, *trnE-TTC*, *trnP-TGG*, *trnY-GTA*, and *trnM-CAT*, exist in two or three copies, exhibiting significant structural variation and species specificity within the mitochondrial genome. While plant mitochondria show considerable diversity in genome size, most mitochondrial PCGs are highly conserved, primarily comprised 24 core conserved genes and 17 variable genes, which can be categorized into complex I (*nad*), complex II (*sdh*), complex III (*cob*), complex IV (*cox*), complex V (*atp*), cytochrome c biosynthesis genes (*ccm*), and tRNA genes (Møller et al., 2021). The *C. fungigraminus* mitochondrial genome includes 24 core conserved genes, with the variable gene *sdh3* being absent and *sdh4* being a pseudo-gene. The presence of the *sdh4* pseudogene in the *Agropyron cristatum* mitochondrial genome in Poaceae indicates that its function has been transferred to nuclear genes (Ou et al., 2024).

### 4.2 RSCU values

When the RSCU value of a codon is greater than 1, its usage rate exceeds that of other synonymous codons, indicating a preference for that codon (Liu et al., 2020). In *C. fungigraminus*, there are 31 preferred codons, while the maximum number in *Zea perennis* and *Eleusine inadica* is 30, and the smallest number in *Triticum timopheevii* and *Chrysopogon zizanioides* is 25, with A or U commonly appearing in the third position of these preferred codons (Yang et al., 2024). This indicates a similarity in the third base codon usage among Poaceae, suggesting that the codon usage pattern may be relatively conserved in the Poaceae evolutionary relationship and it is not likely influenced by the mitochondrial base composition preference (Sloan and Wu, 2014). Despite different tRNA types present in Poaceae, similar codon usage preferences are observed, consistent with the mitochondrial codon usage bias.

### 4.3 RNA editing

As a necessary step for gene expression in the plant mitochondrial genome, RNA editing is widely present and it is an important gene regulation mechanism (Small et al., 2023). The RNA editing sites contained in the Poaceae mitochondrial genomes varies significantly, and not all 27 Poaceae mitochondrial genomes contain corresponding RNA editing sites. Similarly, not all Poaceae mitochondrial genomes contain ORFs, and most ORF functions remain unrecognized (Li et al., 2023). In *C. fungigraminus*, many amino acid changes induced by RNA editing introduce more hydrophobic amino acids into protein structures, potentially playing a key role in regulating mitochondrial gene expression.

### 4.4 Repetitive sequences

Existing research indicates that a large number of repetitive sequences mediate high-frequency recombination within or between higher plant mitochondrial DNA molecules, making them a primary reason for the structural diversity (Cole et al., 2018). During the plant mitochondrial genome evolutionary process, irreversible recombination mediated by short repetitive sequences with low recombination activity plays an important role, and reversible recombination mediated by long repetitive sequences with high recombination activity leads to continuous genome expansion and complexity (Yang et al., 2023b). Over 80% of the *C. fungigraminus* mitochondrial genome consists of non-coding regions, with coding regions only accounting for 19.23%, which may be due to the gradual increase of repetitive sequences (Wynn and Christensen, 2019).

A total of 235 repetitive sequences were checked, including 128 pairs of forward repeats and 107 pairs of palindromic repeats, suggesting frequent molecular recombination. This frequent recombination may have a vital role in dynamically altering the mitochondrial genome conformation and composition, resulting in the assembly of a linear genome (Cole et al., 2018). Additionally, 93 SSRs were discriminated, distributed across different *C. fungigraminus* mitochondrial genomic regions, with a general A/T bias. The most common SSR type was tetranucleotide repeats (35%), followed by pentanucleotide repeats (20%), similarity to the component of the *Perilla frutescens* mitochondrial genome, composed of forward repeats and palindromic repeats, and tetranucleotide and dinucleotide SSRs (Wang et al., 2024b). The repetitive sequence composition reflects both the similarity and the specificity in plant mitochondrial genomes.

### 4.5 Ka/Ks values

Genetically, the Ka/Ks ratio helps infer natural selection effect acting on homologous PCGs. This ratio provides a stronger neutral evolution model test, requiring fewer assumptions (Kang et al., 2020). When the Ka/Ks ratio < 1, it indicates purifying selection, while a ratio > 1 indicates positive selection, and a ratio of 1 indicates neutral selection. Importantly, unless there are at least some advantageous mutations, the Ka/Ks ratio is unlikely to be greater than 1 (Gualberto and Newton, 2017). The Ka/Ks ratio was calculated by comparing 32 C*. fungigraminus* PCGs with 26 other Poaceae plants (**Figure 8**). Almost all Ka/Ks ratios were < 1, indicating that most PCGs are under stabilizing selection. Combining the information from **Figure 8** and **Supplementary Table S11**, the Ka/Ks value for all complex I-V genes were < 1, demonstrating that they are highly conserved. Most of mitochondrial genes (Ka/Ks < 1) may play a vital role in maintaining normal mitochondrial function (Warren and Sloan, 2020).

As shown in **Figure 8**, the Ka/Ks ratio for *ccmB* is > 1, suggesting that *ccmB* may have undergone positive selection after diverging from common ancestor gene. The Ka/Ks value of *ccmB* (3.2) between *C. fungigraminus* and *Oryza minuta* is significantly > 1, indicating positive natural selection, which can be associated with adaptive evolution. A Ka/Ks ratio > 1 for *ccmB* was also detected in *Mesona chinensis*, suggesting that this gene is commonly under selection (Tang et al., 2023a). Additionally, the Ka/Ks ratios for *nad2* and *rps7* are also > 1, indicating that these genes are in a state of positive selection. The high Ka/Ks value of *nad2* may be critically important for the *C. fungigraminus* evolution and it is related to environmental adaptability (Wang et al., 2024c). It is speculated that C. fungigraminus lives in poor soil and requires the *nad2* gene to adapt to extreme poor living environments, so this gene is positively selected. Reports of Ka/Ks ratios > 1 for other mitochondrial genes, such as *rps7*, suggest that different mitochondrial genes may have been subjected to varying selection pressures (Liao et al., 2018). Most importantly, the Ka/Ks ratio of homologous gene pairs is an average value based on the entire sequence. As a result, the overall value can be less than one even in the presence of positive selection at specific sites, since other sites may be subject to purifying selection.

### 4.6 Chloroplast DNA transfer

When exogenous genes are inserted into the mitochondrial genome, preferentially integrating into intergenic regions. The DNA length from different species’ chloroplast genes integrated into the mitochondrial genome varies, typically ranging from 1% to 12% of the chloroplast genome sequences in angiosperms (Garcia et al., 2021). For instance, this is 1.7% in *Garcinia mangostana* (Wee et al., 2022) and 11.6% in *Cucurbita pepo* (Alverson et al., 2010), which is one of the main reasons leading to differences in plant mitochondrial genome size. Therefore, tracking gene transfer is crucial for exploring the plant mitochondrial genome evolution (Bergthorsson et al., 2003). The tRNA gene sequence transfer is common from plant chloroplast to mitochondrial genome (Chevigny et al., 2020). In this study, *rpl2* and 13 tRNA genes from *C. fungigraminus* chloroplast were completely transferred to its mitochondrial genome, where chloroplast-derived tRNAs may have potential functional complementation. Additionally, a large number of chloroplast gene fragments were identified to have migrated to its mitochondrial genome, containing genes that play important roles in the chloroplast, although their function in the mitochondrial genome remains unclear.

### 4.7 Changes in structure and composition of Poaceae genomes

Plant mitochondrial genomes exhibit complex structure with two significant characteristics: different species have mitochondrial genomes of varying sizes, ranging from 200 kb to 2.34 Mb; and there is considerable variation and frequent recombination among the forms of mitochondrial genomes, as well as horizontal gene transfer phenomena (Morley and Nielsen, 2017; Gualberto and Newton, 2017). Compared to other Poaceae mitochondrial genomes, the *C. fungigraminus* mitochondrial genome is moderate size, similar to that of the published *Aegilops longissima* mitochondrial genome (399,005 bp), although its genome is a circular structure (Noyszewski et al., 2014). This indicates significant mitochondrial genome structural variation and species specificity. The GC content is an important species assessment factor (Liu et al., 2020), with the GC content of the *C. fungigraminus* mitochondrial genome being 43.98%. This result is similar to other Poaceae species, such as *Elymus magellanicus* at 42.27%, *Tripsacum dactyloides* at 43.93%, and *Aegilops* sp*eltoides* var. ligustica at 44.43%, suggesting that the GC content is relatively conserved.

In higher plants, the types of PCGs in the mitochondria are relatively consistent, but their numbers, positions, and arrangements often differ among different species and even among different varieties (Grosser et al., 2023). Although the gene products encoded by mitochondrial genomes of 27 Poaceae species are almost identical and the types of tRNA used are also similar, the number of PCGs and tRNA type varies among different species. Overall, there is a significant gene loss phenomenon in the cytochrome c synthesis and ribosomal protein genes. The relatively conserved five complex genes also exhibit differences in mitochondria gene loss (Tang et al., 2023b). Among the 27 Poaceae mitochondrial genomes, aside from varying degrees of gene loss in complex I genes and ribosomal protein genes, there are no losses in cytochrome c synthesis genes and complex I (III, and V) genes. In addition, collinear regions and inversion structures not only reveal the conservation patterns of mitochondrial genomes but also reflect plant evolutionary relationship, providing new perspectives for exploring their genetic basis and evolutionary history (Morley and Nielsen, 2017). It is evident that, aside from complex II, ribosomal proteins, and tRNA genes, other genes in the Poaceae mitochondrial genome are relatively conserved.

### 4.8 Structural changes and phylogeny of Poaceae mitochondrial genomes

In addition to gene loss, frequent structural rearrangements are another characteristic of plant mitochondrial genomes. Reports have indicated that the angiosperm mitochondrial genomes undergo rapid structural differentiation, even demonstrating collinearity loss among closely related species (Cole et al., 2018; Sun et al., 2022). The mitochondrial genome collinearity among 27 Poaceae plants also reflects this characteristic, with considerable structural variation and low collinearity levels among mitochondrial genomes of different genera. Nevertheless, a certain number of conserved gene clusters remain.

During the evolutionary process, frequent PCG losses occur, and the phylogenetic relationship provides a background for further exploring gene losses (Liu et al., 2022). The ML phylogenetic tree constructed based on 32 conserved PCGs indicates that *C. fungigraminus* has the closest phylogenetic relationship with *Saccharum officinarum*. Moreover, the phylogenetic relationship among the 27 Poaceae species is consistent with previously published research findings (Huang et al., 2022). Therefore, it is further demonstrated that phylogenetic analyses can be used for analyzing Poaceae genetic relationship and provide references for phylogenetic studies among other genera and families, based on mitochondrial genome sequences.

## 5 Conclusion

*C. fungigraminus* is a fiber plant with significant economic and energy utilization value. This study assembled the *C. fungigraminus* mitochondrial genome, achieving a comprehensive comparison of organelle genomes, and providing broader perspectives for studying gene transfer between mitochondria and chloroplast. Furthermore, through phylogenetic analysis of 27 Poaceae mitochondrial genomes, the *C. fungigraminus* evolutionary status was confirmed. This study will provide theoretical references for the genetic variation, genome evolution, and breeding research of *C. fungigraminus*, guiding its cultivation, development, and utilization, and indicating the necessity to further obtain mitochondrial genome information of smaller taxonomic groups for more accurately determining the Poaceae phylogenetic relationship.

## Data Availability

The datasets presented in this study can be found in online repositories. The names of the repository/repositories and accession number(s) can be found in the article/**Supplementary Material**.
