# Supplementary material for: The complete mitochondrial genome of Cenchrus fungigraminus indicates structural dynamics and sequence divergences in Poaceae family
Source: Front Plant Sci. 2025 May 30;16:1589847. doi: 10.3389/fpls.2025.1589847 (PMC12162664; doi:10.3389/fpls.2025.1589847)
Supplement: Supplementary file 1 [file Table1.docx]

**Supplementary table 1. Gene composition in** ***Cenchrus fungigraminus* mitochondrial genome**

| **Group of genes** | **Gene name** |
| --- | --- |
| ATP synthase | *atp1, atp4, atp6(2), atp8, atp9* |
| Cytohrome c biogenesis | *ccmB, ccmC, ccmFc*, ccmFn* |
| Ubiquinol cytochrome c reductase | *cob* |
| Cytochrome c oxidase | *cox1(2), cox2*, cox3* |
| Maturases | *matR* |
| Transport membrane protein | *mttB* |
| NADH dehydrogenase | *nad1,**** nad2,**** nad3, nad4***, nad4L, nad5****, nad6, nad7****, nad9* |
| Ribosomal proteins (LSU) | *#rpl2, rpl16* |
| Ribosomal proteins (SSU) | *rps1, rps12, rps13, rps2, rps3*, rps4, rps7* |
| Succinate dehydrogenase | *#sdh4* |
| Ribosomal RNAs | *rrn18(2), rrn26(2), rrn5(2)* |
| Transfer RNAs | *trnC-GCA, trnD-GTC, trnE-TTC(2), trnF-GAA, trnF-GAA*, trnH-GTG, trnK-TTT, trnM-CAT(3), trnN-GTT, trnP-TGG(2), trnQ-TTG, trnS-GCT, trnS-GGA, trnS-TGA, trnV-GAC, trnW-CCA, trnY-GTA(2)* |

Notes: *:intron number; #Gene:Pseudo gene; Gene(2):Number of copies of multi-copy genes;

**Supplementary table 2. Gene characteristics in *Cenchrus fungigraminus* mitochondrial genome**

| **Group of genes** | **Gene name** | **Length** | **Start codon** | **Stop codon** | **Amino acid** |
| --- | --- | --- | --- | --- | --- |
| ATP synthase | *atp1* | 1542 | ATG | TAG | 514 |
|  | *atp4* | 618 | ATG | TAA | 206 |
|  | *atp6* | 969 | ATG | CAA(TAA) | 323 |
|  | *atp6* | 969 | ATG | CAA(TAA) | 323 |
|  | *atp8* | 1185 | ATG | TAA | 395 |
|  | *atp9* | 225 | ATG | TAG | 75 |
| Cytohrome c biogenesis | *ccmB* | 621 | ATG | TGA | 207 |
|  | *ccmC* | 723 | ATG | TAG | 241 |
|  | *ccmFc* | 1314 | ATG | CGA(TGA) | 438 |
|  | *ccmFn* | 1734 | ATG | TGA | 578 |
| Ubichinol cytochrome c reductase | *cob* | 1167 | ATG | TAG | 389 |
| Cytochrome c oxidase | *cox1* | 2229 | ATG | TAG | 743 |
|  | *cox1* | 2163 | ATG | TAA | 721 |
|  | *cox2* | 783 | ATG | TAA | 261 |
|  | *cox3* | 798 | ATG | TGA | 266 |
| Maturases | *matR* | 1977 | ATG | TAG | 659 |
| Transport membrance protein | *mttB* | 348 | ATG | TAG | 116 |
| NADH dehydrogenase | *nad1* | 978 | ACG(ATG) | TAA | 326 |
|  | *nad2* | 1467 | ATG | TAA | 489 |
|  | *nad3* | 357 | ATG | TAA | 119 |
|  | *nad4* | 1488 | ATG | TGA | 496 |
|  | *nad4L* | 303 | ACG(ATG) | TAA | 101 |
|  | *nad5* | 2013 | ATG | TAA | 671 |
|  | *nad6* | 1158 | ATG | TAG | 386 |
|  | *nad7* | 1185 | ATG | TAG | 395 |
|  | *nad9* | 573 | ATG | TAA | 191 |
| Ribosomal proteins (LSU) | *rpl16* | 516 | ATG | TAA | 172 |
| Ribosomal proteins (SSU) | *rps1* | 654 | ATG | TAG | 218 |
|  | *rps12* | 378 | ATG | TGA | 126 |
|  | *rps13* | 351 | ATG | TGA | 117 |
|  | *rps2* | 1038 | ATG | TAA | 346 |
|  | *rps3* | 1671 | ATG | TAG | 557 |
|  | *rps4* | 1182 | ATG | TAA | 394 |
|  | *rps7* | 447 | ATG | TAA | 149 |
| Ribosomal RNAs | *rrn18* | 1968 |  |  |  |
|  | *rrn18* | 1968 |  |  |  |
|  | *rrn26* | 3470 |  |  |  |
|  | *rrn26* | 3470 |  |  |  |
|  | *rrn5* | 121 |  |  |  |
|  | *rrn5* | 121 |  |  |  |
| Transfer RNAs | *trnC-GCA* | 71 |  |  |  |
|  | *trnD-GTC* | 74 |  |  |  |
|  | *trnE-TTC* | 73 |  |  |  |
|  | *trnE-TTC* | 73 |  |  |  |
|  | *trnF-GAA* | 70 |  |  |  |
|  | *trnF-GAA* | 73 |  |  |  |
|  | *trnH-GTG* | 74 |  |  |  |
|  | *trnK-TTT* | 73 |  |  |  |
|  | *trnM-CAT* | 74 |  |  |  |
|  | *trnM-CAT* | 74 |  |  |  |
|  | *trnM-CAT* | 73 |  |  |  |
|  | *trnN-GTT* | 72 |  |  |  |
|  | *trnP-TGG* | 75 |  |  |  |
|  | *trnP-TGG* | 74 |  |  |  |
|  | *trnQ-TTG* | 72 |  |  |  |
|  | *trnS-GCT* | 88 |  |  |  |
|  | *trnS-GGA* | 87 |  |  |  |
|  | *trnS-TGA* | 87 |  |  |  |
|  | *trnV-GAC* | 72 |  |  |  |
|  | *trnW-CCA* | 74 |  |  |  |
|  | *trnY-GTA* | 72 |  |  |  |
|  | *trnY-GTA* | 83 |  |  |  |

**Supplementary table 3. Dispersed repeat sequences** **in *Cenchrus fungigraminus* mitochondrial genome**

| **#Chr1** | **Chr2** | **type** | **alignment length** | **similarity** | **start1** | **end1** | **start2** | **end2** | **evalue** |
| --- | --- | --- | --- | --- | --- | --- | --- | --- | --- |
| chr1 | chr1 | F | 17557 | 99.989 | 281797 | 299353 | 32773 | 50328 | 0 |
| chr1 | chr1 | F | 9950 | 100 | 239014 | 248963 | 11645 | 21594 | 0 |
| chr1 | chr1 | F | 580 | 89.483 | 249096 | 249674 | 218915 | 219479 | 0 |
| chr1 | chr1 | F | 228 | 98.684 | 384896 | 385123 | 162106 | 162333 | ####### |
| chr1 | chr1 | F | 218 | 100 | 385927 | 386144 | 8127 | 8344 | ####### |
| chr1 | chr1 | P | 203 | 99.015 | 219596 | 219798 | 161251 | 161453 | 2.32E-99 |
| chr1 | chr1 | F | 190 | 100 | 234504 | 234693 | 203575 | 203764 | 1.80E-95 |
| chr1 | chr1 | P | 187 | 99.465 | 344079 | 344265 | 149174 | 149359 | 1.40E-91 |
| chr1 | chr1 | P | 194 | 96.907 | 133027 | 133219 | 40499 | 40692 | 3.93E-87 |
| chr1 | chr1 | P | 197 | 96.447 | 289523 | 289719 | 133024 | 133219 | 3.93E-87 |
| chr1 | chr1 | P | 197 | 96.447 | 40499 | 40695 | 133024 | 133219 | 3.93E-87 |
| chr1 | chr1 | P | 194 | 96.907 | 133027 | 133219 | 289523 | 289716 | 3.93E-87 |
| chr1 | chr1 | F | 208 | 92.788 | 183526 | 183733 | 10183 | 10386 | 2.38E-79 |
| chr1 | chr1 | P | 204 | 92.647 | 336214 | 336417 | 10830 | 11033 | 3.08E-78 |
| chr1 | chr1 | P | 169 | 96.45 | 207490 | 207657 | 139697 | 139865 | 3.10E-73 |
| chr1 | chr1 | P | 190 | 93.158 | 269933 | 270121 | 153202 | 153391 | 3.10E-73 |
| chr1 | chr1 | P | 171 | 95.906 | 363169 | 363338 | 182761 | 182929 | 4.01E-72 |
| chr1 | chr1 | F | 170 | 94.118 | 265142 | 265310 | 80103 | 80272 | 4.04E-67 |
| chr1 | chr1 | F | 342 | 78.655 | 189477 | 189811 | 42809 | 43147 | 1.91E-55 |
| chr1 | chr1 | F | 344 | 78.779 | 291833 | 292171 | 189477 | 189811 | 1.91E-55 |
| chr1 | chr1 | P | 116 | 97.414 | 233280 | 233395 | 4739 | 4854 | 2.49E-49 |
| chr1 | chr1 | P | 113 | 98.23 | 4742 | 4854 | 233280 | 233392 | 2.49E-49 |
| chr1 | chr1 | F | 180 | 86.111 | 65572 | 65751 | 40149 | 40328 | 3.21E-48 |
| chr1 | chr1 | F | 180 | 86.111 | 289173 | 289352 | 65572 | 65751 | 3.21E-48 |
| chr1 | chr1 | P | 102 | 100 | 81672 | 81773 | 42527 | 42628 | 1.50E-46 |
| chr1 | chr1 | P | 102 | 100 | 291551 | 291652 | 81672 | 81773 | 1.50E-46 |
| chr1 | chr1 | P | 126 | 93.651 | 366946 | 367066 | 168880 | 169005 | 6.96E-45 |
| chr1 | chr1 | P | 177 | 86.441 | 357873 | 358037 | 21751 | 21922 | 3.24E-43 |
| chr1 | chr1 | P | 174 | 86.782 | 21754 | 21922 | 357873 | 358034 | 3.24E-43 |
| chr1 | chr1 | F | 94 | 98.936 | 309255 | 309348 | 52201 | 52294 | 1.95E-40 |
| chr1 | chr1 | F | 125 | 91.2 | 375720 | 375839 | 80162 | 80286 | 2.52E-39 |
| chr1 | chr1 | F | 95 | 96.842 | 338389 | 338483 | 126164 | 126258 | 1.17E-37 |
| chr1 | chr1 | F | 86 | 98.837 | 238118 | 238203 | 161250 | 161335 | 5.46E-36 |
| chr1 | chr1 | F | 81 | 98.765 | 276383 | 276463 | 276356 | 276436 | 3.28E-33 |
| chr1 | chr1 | F | 87 | 96.552 | 358681 | 358766 | 65008 | 65094 | 1.18E-32 |
| chr1 | chr1 | P | 99 | 92.929 | 238105 | 238203 | 219714 | 219811 | 1.18E-32 |
| chr1 | chr1 | P | 105 | 91.429 | 219714 | 219817 | 238099 | 238203 | 1.18E-32 |
| chr1 | chr1 | F | 75 | 100 | 182752 | 182826 | 53146 | 53220 | 1.53E-31 |
| chr1 | chr1 | P | 75 | 98.667 | 269644 | 269718 | 202020 | 202094 | 7.11E-30 |
| chr1 | chr1 | P | 93 | 93.548 | 359484 | 359575 | 214102 | 214189 | 7.11E-30 |
| chr1 | chr1 | F | 72 | 98.611 | 358125 | 358196 | 238319 | 238390 | 3.31E-28 |
| chr1 | chr1 | F | 70 | 98.571 | 15820 | 15889 | 9371 | 9440 | 4.28E-27 |
| chr1 | chr1 | F | 70 | 98.571 | 243189 | 243258 | 9371 | 9440 | 4.28E-27 |
| chr1 | chr1 | P | 70 | 98.571 | 118852 | 118921 | 9373 | 9442 | 4.28E-27 |
| chr1 | chr1 | P | 67 | 100 | 281547 | 281613 | 136276 | 136342 | 4.28E-27 |
| chr1 | chr1 | F | 68 | 98.529 | 358628 | 358695 | 162087 | 162154 | 5.53E-26 |
| chr1 | chr1 | P | 99 | 88.889 | 384245 | 384343 | 326222 | 326319 | 5.53E-26 |
| chr1 | chr1 | P | 65 | 98.462 | 118857 | 118921 | 15822 | 15886 | 2.57E-24 |
| chr1 | chr1 | P | 68 | 97.059 | 243191 | 243258 | 118854 | 118921 | 2.57E-24 |
| chr1 | chr1 | P | 68 | 97.059 | 15822 | 15889 | 118854 | 118921 | 2.57E-24 |
| chr1 | chr1 | P | 65 | 98.462 | 118857 | 118921 | 243191 | 243255 | 2.57E-24 |
| chr1 | chr1 | F | 62 | 100 | 315412 | 315473 | 280799 | 280860 | 2.57E-24 |
| chr1 | chr1 | P | 61 | 100 | 164455 | 164515 | 41101 | 41161 | 9.26E-24 |
| chr1 | chr1 | P | 68 | 97.059 | 265170 | 265237 | 164135 | 164201 | 9.26E-24 |
| chr1 | chr1 | P | 61 | 100 | 290125 | 290185 | 164455 | 164515 | 9.26E-24 |
| chr1 | chr1 | P | 71 | 95.775 | 164135 | 164204 | 265167 | 265237 | 9.26E-24 |
| chr1 | chr1 | P | 64 | 96.875 | 139574 | 139637 | 46121 | 46184 | 4.31E-22 |
| chr1 | chr1 | P | 71 | 94.366 | 164135 | 164204 | 80128 | 80198 | 4.31E-22 |
| chr1 | chr1 | P | 67 | 95.522 | 295145 | 295211 | 139571 | 139637 | 4.31E-22 |
| chr1 | chr1 | P | 67 | 95.522 | 46121 | 46187 | 139571 | 139637 | 4.31E-22 |
| chr1 | chr1 | P | 68 | 95.588 | 80131 | 80198 | 164135 | 164201 | 4.31E-22 |
| chr1 | chr1 | P | 64 | 96.875 | 139574 | 139637 | 295145 | 295208 | 4.31E-22 |
| chr1 | chr1 | F | 111 | 84.685 | 375720 | 375825 | 265201 | 265310 | 1.55E-21 |
| chr1 | chr1 | P | 56 | 100 | 67724 | 67779 | 15835 | 15890 | 5.57E-21 |
| chr1 | chr1 | F | 89 | 88.764 | 311685 | 311772 | 34051 | 34134 | 5.57E-21 |
| chr1 | chr1 | P | 62 | 96.774 | 308818 | 308879 | 35673 | 35734 | 5.57E-21 |
| chr1 | chr1 | P | 56 | 100 | 243204 | 243259 | 67724 | 67779 | 5.57E-21 |
| chr1 | chr1 | P | 71 | 92.958 | 259412 | 259482 | 143147 | 143217 | 5.57E-21 |
| chr1 | chr1 | P | 68 | 94.118 | 143150 | 143217 | 259412 | 259479 | 5.57E-21 |
| chr1 | chr1 | F | 89 | 88.764 | 311685 | 311772 | 283075 | 283158 | 5.57E-21 |
| chr1 | chr1 | P | 62 | 96.774 | 308818 | 308879 | 284697 | 284758 | 5.57E-21 |
| chr1 | chr1 | P | 62 | 96.774 | 311369 | 311430 | 248967 | 249027 | 2.00E-20 |
| chr1 | chr1 | F | 71 | 92.958 | 324123 | 324193 | 238116 | 238182 | 7.21E-20 |
| chr1 | chr1 | P | 56 | 98.214 | 384964 | 385019 | 218738 | 218793 | 2.59E-19 |
| chr1 | chr1 | P | 55 | 98.182 | 67725 | 67779 | 9386 | 9440 | 9.33E-19 |
| chr1 | chr1 | P | 114 | 82.456 | 265476 | 265585 | 66017 | 66130 | 9.33E-19 |
| chr1 | chr1 | F | 69 | 92.754 | 324125 | 324193 | 161250 | 161314 | 9.33E-19 |
| chr1 | chr1 | F | 58 | 96.552 | 319205 | 319262 | 102742 | 102798 | 3.35E-18 |
| chr1 | chr1 | F | 54 | 98.148 | 276410 | 276463 | 276356 | 276409 | 3.35E-18 |
| chr1 | chr1 | F | 49 | 100 | 375709 | 375757 | 9375 | 9423 | 4.34E-17 |
| chr1 | chr1 | F | 49 | 100 | 375709 | 375757 | 15824 | 15872 | 4.34E-17 |
| chr1 | chr1 | F | 55 | 96.364 | 118854 | 118908 | 67725 | 67779 | 4.34E-17 |
| chr1 | chr1 | P | 49 | 100 | 375709 | 375757 | 118871 | 118919 | 4.34E-17 |
| chr1 | chr1 | F | 53 | 98.113 | 277866 | 277917 | 140820 | 140872 | 4.34E-17 |
| chr1 | chr1 | P | 49 | 100 | 251313 | 251361 | 150314 | 150362 | 4.34E-17 |
| chr1 | chr1 | P | 49 | 100 | 218738 | 218786 | 162181 | 162229 | 4.34E-17 |
| chr1 | chr1 | F | 49 | 100 | 375709 | 375757 | 243193 | 243241 | 4.34E-17 |
| chr1 | chr1 | F | 49 | 100 | 384896 | 384944 | 358647 | 358695 | 4.34E-17 |
| chr1 | chr1 | P | 52 | 98.077 | 118871 | 118922 | 375706 | 375757 | 4.34E-17 |
| chr1 | chr1 | F | 48 | 100 | 44087 | 44134 | 14148 | 14195 | 1.56E-16 |
| chr1 | chr1 | F | 48 | 100 | 293111 | 293158 | 14148 | 14195 | 1.56E-16 |
| chr1 | chr1 | F | 66 | 90.909 | 139702 | 139767 | 34224 | 34289 | 1.56E-16 |
| chr1 | chr1 | F | 48 | 100 | 241517 | 241564 | 44087 | 44134 | 1.56E-16 |
| chr1 | chr1 | F | 66 | 90.909 | 283248 | 283313 | 139702 | 139767 | 1.56E-16 |
| chr1 | chr1 | P | 48 | 100 | 391487 | 391534 | 214016 | 214063 | 1.56E-16 |
| chr1 | chr1 | F | 48 | 100 | 293111 | 293158 | 241517 | 241564 | 1.56E-16 |
| chr1 | chr1 | F | 52 | 98.077 | 365784 | 365834 | 365734 | 365785 | 1.56E-16 |
| chr1 | chr1 | P | 73 | 89.041 | 324121 | 324193 | 219735 | 219803 | 5.61E-16 |
| chr1 | chr1 | P | 55 | 94.545 | 372118 | 372172 | 50164 | 50218 | 2.02E-15 |
| chr1 | chr1 | P | 55 | 94.545 | 364258 | 364312 | 64665 | 64719 | 2.02E-15 |
| chr1 | chr1 | F | 49 | 97.959 | 164133 | 164181 | 67741 | 67789 | 2.02E-15 |
| chr1 | chr1 | P | 55 | 94.545 | 372118 | 372172 | 299188 | 299242 | 2.02E-15 |
| chr1 | chr1 | P | 52 | 96.154 | 64668 | 64719 | 364258 | 364309 | 2.02E-15 |
| chr1 | chr1 | P | 52 | 96.154 | 299191 | 299242 | 372118 | 372169 | 2.02E-15 |
| chr1 | chr1 | P | 52 | 96.154 | 50167 | 50218 | 372118 | 372169 | 2.02E-15 |
| chr1 | chr1 | P | 66 | 89.394 | 207587 | 207652 | 34224 | 34289 | 7.26E-15 |
| chr1 | chr1 | F | 45 | 100 | 64466 | 64510 | 39699 | 39743 | 7.26E-15 |
| chr1 | chr1 | P | 68 | 89.706 | 363272 | 363338 | 53155 | 53220 | 7.26E-15 |
| chr1 | chr1 | F | 45 | 100 | 288723 | 288767 | 64466 | 64510 | 7.26E-15 |
| chr1 | chr1 | F | 73 | 89.041 | 357923 | 357995 | 95460 | 95525 | 7.26E-15 |
| chr1 | chr1 | P | 66 | 89.394 | 283248 | 283313 | 207587 | 207652 | 7.26E-15 |
| chr1 | chr1 | P | 50 | 96 | 192743 | 192792 | 10857 | 10906 | 2.61E-14 |
| chr1 | chr1 | P | 47 | 97.872 | 265191 | 265237 | 67743 | 67789 | 2.61E-14 |
| chr1 | chr1 | F | 50 | 96 | 336341 | 336390 | 192743 | 192792 | 2.61E-14 |
| chr1 | chr1 | P | 47 | 97.872 | 383337 | 383383 | 212473 | 212519 | 2.61E-14 |
| chr1 | chr1 | P | 44 | 100 | 67746 | 67789 | 265191 | 265234 | 2.61E-14 |
| chr1 | chr1 | F | 91 | 83.516 | 370128 | 370217 | 325028 | 325115 | 2.61E-14 |
| chr1 | chr1 | P | 44 | 100 | 212476 | 212519 | 383337 | 383380 | 2.61E-14 |
| chr1 | chr1 | P | 42 | 100 | 391555 | 391596 | 19737 | 19778 | 3.38E-13 |
| chr1 | chr1 | F | 48 | 95.833 | 185753 | 185800 | 118851 | 118898 | 3.38E-13 |
| chr1 | chr1 | P | 42 | 100 | 391555 | 391596 | 247106 | 247147 | 3.38E-13 |
| chr1 | chr1 | P | 42 | 100 | 369717 | 369758 | 252559 | 252600 | 3.38E-13 |
| chr1 | chr1 | F | 88 | 82.955 | 34172 | 34259 | 10302 | 10386 | 1.21E-12 |
| chr1 | chr1 | F | 88 | 82.955 | 283196 | 283283 | 10302 | 10386 | 1.21E-12 |
| chr1 | chr1 | F | 69 | 86.957 | 355006 | 355074 | 28157 | 28224 | 1.21E-12 |
| chr1 | chr1 | P | 47 | 95.745 | 80152 | 80198 | 67743 | 67789 | 1.21E-12 |
| chr1 | chr1 | P | 44 | 97.727 | 67746 | 67789 | 80152 | 80195 | 1.21E-12 |
| chr1 | chr1 | P | 50 | 94 | 335060 | 335109 | 272514 | 272563 | 1.21E-12 |
| chr1 | chr1 | P | 53 | 92.453 | 272514 | 272566 | 335057 | 335109 | 1.21E-12 |
| chr1 | chr1 | P | 56 | 91.071 | 363418 | 363473 | 359300 | 359355 | 1.21E-12 |
| chr1 | chr1 | P | 53 | 92.453 | 359303 | 359355 | 363418 | 363470 | 1.21E-12 |
| chr1 | chr1 | P | 49 | 93.878 | 367056 | 367104 | 168822 | 168870 | 4.37E-12 |
| chr1 | chr1 | P | 47 | 95.745 | 326275 | 326320 | 238105 | 238151 | 4.37E-12 |
| chr1 | chr1 | F | 46 | 95.652 | 384245 | 384290 | 238106 | 238151 | 4.37E-12 |
| chr1 | chr1 | F | 60 | 88.333 | 355282 | 355341 | 15409 | 15468 | 1.57E-11 |
| chr1 | chr1 | P | 42 | 97.619 | 185759 | 185800 | 15845 | 15886 | 1.57E-11 |
| chr1 | chr1 | F | 46 | 95.652 | 210136 | 210180 | 18594 | 18639 | 1.57E-11 |
| chr1 | chr1 | P | 53 | 92.453 | 190943 | 190995 | 32657 | 32705 | 1.57E-11 |
| chr1 | chr1 | F | 45 | 95.556 | 185756 | 185800 | 67725 | 67769 | 1.57E-11 |
| chr1 | chr1 | P | 39 | 100 | 367502 | 367540 | 100503 | 100541 | 1.57E-11 |
| chr1 | chr1 | F | 80 | 83.75 | 359996 | 360071 | 104648 | 104727 | 1.57E-11 |
| chr1 | chr1 | P | 45 | 95.556 | 243214 | 243258 | 185756 | 185800 | 1.57E-11 |
| chr1 | chr1 | P | 45 | 95.556 | 15845 | 15889 | 185756 | 185800 | 1.57E-11 |
| chr1 | chr1 | P | 56 | 91.071 | 32657 | 32708 | 190940 | 190995 | 1.57E-11 |
| chr1 | chr1 | F | 46 | 95.652 | 245963 | 246008 | 210136 | 210180 | 1.57E-11 |
| chr1 | chr1 | P | 39 | 100 | 231749 | 231787 | 212533 | 212571 | 1.57E-11 |
| chr1 | chr1 | F | 60 | 88.333 | 355282 | 355341 | 242778 | 242837 | 1.57E-11 |
| chr1 | chr1 | P | 42 | 97.619 | 185759 | 185800 | 243214 | 243255 | 1.57E-11 |
| chr1 | chr1 | P | 47 | 93.617 | 185754 | 185800 | 9396 | 9442 | 5.65E-11 |
| chr1 | chr1 | P | 38 | 100 | 375720 | 375757 | 67742 | 67779 | 5.65E-11 |
| chr1 | chr1 | F | 62 | 87.097 | 316002 | 316063 | 161454 | 161515 | 5.65E-11 |
| chr1 | chr1 | P | 50 | 92 | 9396 | 9445 | 185751 | 185800 | 5.65E-11 |
| chr1 | chr1 | P | 38 | 100 | 325521 | 325558 | 218761 | 218798 | 5.65E-11 |
| chr1 | chr1 | F | 51 | 92.157 | 402587 | 402636 | 231781 | 231831 | 5.65E-11 |
| chr1 | chr1 | F | 89 | 80.899 | 183645 | 183733 | 34172 | 34259 | 2.03E-10 |
| chr1 | chr1 | F | 52 | 90.385 | 315009 | 315060 | 94258 | 94309 | 2.03E-10 |
| chr1 | chr1 | F | 89 | 80.899 | 283196 | 283283 | 183645 | 183733 | 2.03E-10 |
| chr1 | chr1 | F | 37 | 100 | 380226 | 380262 | 328562 | 328598 | 2.03E-10 |
| chr1 | chr1 | P | 39 | 97.436 | 164133 | 164171 | 9386 | 9424 | 7.31E-10 |
| chr1 | chr1 | P | 39 | 97.436 | 164133 | 164171 | 15835 | 15873 | 7.31E-10 |
| chr1 | chr1 | P | 36 | 100 | 93717 | 93752 | 93717 | 93752 | 7.31E-10 |
| chr1 | chr1 | F | 39 | 97.436 | 164133 | 164171 | 118870 | 118908 | 7.31E-10 |
| chr1 | chr1 | F | 55 | 89.091 | 341721 | 341774 | 135651 | 135705 | 7.31E-10 |
| chr1 | chr1 | P | 39 | 97.436 | 243204 | 243242 | 164133 | 164171 | 7.31E-10 |
| chr1 | chr1 | P | 70 | 84.286 | 375688 | 375757 | 164134 | 164202 | 7.31E-10 |
| chr1 | chr1 | F | 36 | 100 | 171230 | 171265 | 170878 | 170913 | 7.31E-10 |
| chr1 | chr1 | P | 46 | 93.478 | 328400 | 328444 | 214052 | 214097 | 7.31E-10 |
| chr1 | chr1 | P | 43 | 95.349 | 214055 | 214097 | 328400 | 328441 | 7.31E-10 |
| chr1 | chr1 | F | 42 | 95.238 | 311751 | 311791 | 32661 | 32702 | 2.63E-09 |
| chr1 | chr1 | P | 39 | 97.436 | 372848 | 372885 | 139323 | 139361 | 2.63E-09 |
| chr1 | chr1 | P | 50 | 90 | 329077 | 329126 | 179478 | 179527 | 2.63E-09 |
| chr1 | chr1 | P | 44 | 93.182 | 179484 | 179527 | 329077 | 329120 | 2.63E-09 |
| chr1 | chr1 | F | 37 | 97.297 | 178679 | 178715 | 3861 | 3897 | 9.46E-09 |
| chr1 | chr1 | F | 34 | 100 | 265201 | 265234 | 9386 | 9419 | 9.46E-09 |
| chr1 | chr1 | F | 34 | 100 | 265201 | 265234 | 15835 | 15868 | 9.46E-09 |
| chr1 | chr1 | P | 79 | 83.544 | 95460 | 95531 | 21799 | 21872 | 9.46E-09 |
| chr1 | chr1 | P | 46 | 91.304 | 238104 | 238149 | 30453 | 30498 | 9.46E-09 |
| chr1 | chr1 | P | 40 | 95 | 384249 | 384288 | 30453 | 30492 | 9.46E-09 |
| chr1 | chr1 | P | 34 | 100 | 191082 | 191115 | 89006 | 89039 | 9.46E-09 |
| chr1 | chr1 | F | 44 | 93.182 | 145517 | 145560 | 96970 | 97012 | 9.46E-09 |
| chr1 | chr1 | P | 37 | 97.297 | 265201 | 265237 | 118872 | 118908 | 9.46E-09 |
| chr1 | chr1 | F | 34 | 100 | 384257 | 384290 | 161250 | 161283 | 9.46E-09 |
| chr1 | chr1 | F | 34 | 100 | 265201 | 265234 | 243204 | 243237 | 9.46E-09 |
| chr1 | chr1 | P | 34 | 100 | 118875 | 118908 | 265201 | 265234 | 9.46E-09 |
| chr1 | chr1 | F | 77 | 81.818 | 34058 | 34132 | 10182 | 10256 | 3.40E-08 |
| chr1 | chr1 | F | 77 | 81.818 | 283082 | 283156 | 10182 | 10256 | 3.40E-08 |
| chr1 | chr1 | F | 39 | 94.872 | 139699 | 139737 | 10348 | 10386 | 3.40E-08 |
| chr1 | chr1 | P | 39 | 94.872 | 207617 | 207655 | 10348 | 10386 | 3.40E-08 |
| chr1 | chr1 | P | 45 | 91.111 | 266561 | 266605 | 65549 | 65593 | 3.40E-08 |
| chr1 | chr1 | P | 57 | 87.719 | 314259 | 314311 | 120309 | 120363 | 3.40E-08 |
| chr1 | chr1 | F | 45 | 91.111 | 186289 | 186333 | 121901 | 121945 | 3.40E-08 |
| chr1 | chr1 | F | 36 | 97.222 | 334909 | 334944 | 178061 | 178096 | 3.40E-08 |
| chr1 | chr1 | P | 36 | 97.222 | 238826 | 238861 | 218692 | 218727 | 3.40E-08 |
| chr1 | chr1 | P | 45 | 91.111 | 375605 | 375649 | 219887 | 219931 | 3.40E-08 |
| chr1 | chr1 | F | 36 | 97.222 | 358351 | 358386 | 238888 | 238923 | 3.40E-08 |
| chr1 | chr1 | F | 36 | 97.222 | 384255 | 384290 | 324123 | 324158 | 3.40E-08 |
| chr1 | chr1 | F | 82 | 81.707 | 311691 | 311770 | 10182 | 10256 | 1.22E-07 |
| chr1 | chr1 | F | 56 | 87.5 | 139396 | 139451 | 21275 | 21325 | 1.22E-07 |
| chr1 | chr1 | P | 35 | 97.143 | 324375 | 324409 | 28160 | 28194 | 1.22E-07 |
| chr1 | chr1 | F | 76 | 81.579 | 183526 | 183599 | 34059 | 34132 | 1.22E-07 |
| chr1 | chr1 | F | 58 | 86.207 | 152665 | 152722 | 102745 | 102798 | 1.22E-07 |
| chr1 | chr1 | F | 56 | 87.5 | 248644 | 248694 | 139396 | 139451 | 1.22E-07 |
| chr1 | chr1 | F | 41 | 92.683 | 315535 | 315575 | 143477 | 143517 | 1.22E-07 |
| chr1 | chr1 | F | 59 | 86.441 | 319208 | 319262 | 152665 | 152722 | 1.22E-07 |
| chr1 | chr1 | F | 76 | 81.579 | 283083 | 283156 | 183526 | 183599 | 1.22E-07 |
| chr1 | chr1 | F | 42 | 92.857 | 275728 | 275769 | 216320 | 216360 | 1.22E-07 |
| chr1 | chr1 | F | 34 | 97.059 | 80162 | 80195 | 9386 | 9419 | 4.40E-07 |
| chr1 | chr1 | F | 34 | 97.059 | 80162 | 80195 | 15835 | 15868 | 4.40E-07 |
| chr1 | chr1 | P | 34 | 97.059 | 333539 | 333572 | 46143 | 46176 | 4.40E-07 |
| chr1 | chr1 | F | 34 | 97.059 | 243204 | 243237 | 80162 | 80195 | 4.40E-07 |
| chr1 | chr1 | P | 34 | 97.059 | 118875 | 118908 | 80162 | 80195 | 4.40E-07 |
| chr1 | chr1 | P | 37 | 94.595 | 107690 | 107726 | 86867 | 86903 | 4.40E-07 |
| chr1 | chr1 | P | 37 | 94.595 | 80162 | 80198 | 118872 | 118908 | 4.40E-07 |
| chr1 | chr1 | P | 34 | 97.059 | 326275 | 326308 | 161250 | 161283 | 4.40E-07 |
| chr1 | chr1 | F | 35 | 97.143 | 272617 | 272650 | 168783 | 168817 | 4.40E-07 |
| chr1 | chr1 | F | 81 | 81.481 | 311692 | 311770 | 183526 | 183599 | 4.40E-07 |
| chr1 | chr1 | P | 34 | 97.059 | 333539 | 333572 | 295167 | 295200 | 4.40E-07 |
| chr1 | chr1 | F | 30 | 100 | 13276 | 13305 | 7393 | 7422 | 1.58E-06 |
| chr1 | chr1 | F | 30 | 100 | 240645 | 240674 | 7393 | 7422 | 1.58E-06 |
| chr1 | chr1 | P | 89 | 78.652 | 357518 | 357604 | 7533 | 7619 | 1.58E-06 |
| chr1 | chr1 | F | 59 | 84.746 | 326277 | 326332 | 30453 | 30510 | 1.58E-06 |
| chr1 | chr1 | F | 53 | 86.792 | 219768 | 219817 | 30453 | 30505 | 1.58E-06 |
| chr1 | chr1 | F | 43 | 90.698 | 139399 | 139441 | 116560 | 116600 | 1.58E-06 |
| chr1 | chr1 | P | 68 | 82.353 | 315410 | 315474 | 119529 | 119596 | 1.58E-06 |
| chr1 | chr1 | F | 46 | 89.13 | 365825 | 365870 | 210887 | 210931 | 1.58E-06 |
| chr1 | chr1 | P | 36 | 94.444 | 326275 | 326310 | 324123 | 324158 | 1.58E-06 |
| chr1 | chr1 | F | 33 | 96.97 | 384964 | 384996 | 325526 | 325558 | 1.58E-06 |
| chr1 | chr1 | F | 42 | 90.476 | 353674 | 353715 | 326274 | 326315 | 1.58E-06 |
| chr1 | chr1 | P | 43 | 90.698 | 384249 | 384291 | 353674 | 353715 | 1.58E-06 |
| chr1 | chr1 | P | 92 | 78.261 | 7533 | 7622 | 357515 | 357604 | 1.58E-06 |
| chr1 | chr1 | F | 35 | 94.286 | 136689 | 136723 | 4101 | 4135 | 5.69E-06 |
| chr1 | chr1 | P | 29 | 100 | 301510 | 301538 | 60655 | 60683 | 5.69E-06 |
| chr1 | chr1 | P | 36 | 94.444 | 256848 | 256883 | 97666 | 97700 | 5.69E-06 |
| chr1 | chr1 | P | 42 | 90.476 | 353675 | 353715 | 238110 | 238151 | 5.69E-06 |
| chr1 | chr1 | P | 42 | 90.476 | 97666 | 97706 | 256842 | 256883 | 5.69E-06 |

F:Forward dispersed repeats；P: palindromic dispersed repeats

**Supplementary table 4. SSR type in *Cenchrus fungigraminus* mitochondrial genome**

| **ID** | **SSR nr.** | **SSR type** | **SSR** | **size** | **start** | **end** |
| --- | --- | --- | --- | --- | --- | --- |
| chr1 | 1 | p5 | (TATTG)14 | 70 | 3727 | 3796 |
| chr1 | 2 | p4 | (TTTC)3 | 12 | 5810 | 5821 |
| chr1 | 3 | p5 | (TTTAT)24 | 120 | 17387 | 17506 |
| chr1 | 4 | p3 | (AAG)4 | 12 | 20850 | 20861 |
| chr1 | 5 | p6 | (CTTTGG)3 | 18 | 26875 | 26892 |
| chr1 | 6 | p5 | (TTCTA)5 | 25 | 40916 | 40940 |
| chr1 | 7 | p2 | (TA)5 | 10 | 41473 | 41482 |
| chr1 | 8 | p5 | (TTCTA)5 | 25 | 47634 | 47658 |
| chr1 | 9 | p1 | (T)10 | 10 | 51318 | 51327 |
| chr1 | 10 | p5 | (ACTAT)3 | 15 | 53331 | 53345 |
| chr1 | 11 | p1 | (A)10 | 10 | 56681 | 56690 |
| chr1 | 12 | p4 | (AGTC)3 | 12 | 57341 | 57352 |
| chr1 | 13 | p4 | (TTGG)3 | 12 | 60447 | 60458 |
| chr1 | 14 | p1 | (A)10 | 10 | 61161 | 61170 |
| chr1 | 15 | p4 | (GCTT)3 | 12 | 63642 | 63653 |
| chr1 | 16 | p5 | (ATTTG)16 | 80 | 69869 | 69948 |
| chr1 | 17 | p1 | (A)10 | 10 | 73100 | 73109 |
| chr1 | 18 | p2 | (AT)5 | 10 | 89404 | 89413 |
| chr1 | 19 | p4 | (CATC)3 | 12 | 93051 | 93062 |
| chr1 | 20 | p2 | (CT)5 | 10 | 93767 | 93776 |
| chr1 | 21 | p4 | (CTCC)3 | 12 | 96617 | 96628 |
| chr1 | 22 | p2 | (AG)5 | 10 | 101680 | 101689 |
| chr1 | 23 | p5 | (TTCTA)17 | 85 | 105164 | 105248 |
| chr1 | 24 | p6 | (ATTTCG)9 | 54 | 108425 | 108478 |
| chr1 | 25 | p3 | (CTT)4 | 12 | 111277 | 111288 |
| chr1 | 26 | p2 | (TC)5 | 10 | 115423 | 115432 |
| chr1 | 27 | p5 | (CCTAA)6 | 30 | 116072 | 116101 |
| chr1 | 28 | p3 | (AGT)4 | 12 | 116101 | 116112 |
| chr1 | 29 | p4 | (ACTG)3 | 12 | 117865 | 117876 |
| chr1 | 30 | p3 | (TCT)4 | 12 | 120000 | 120011 |
| chr1 | 31 | p5 | (TAGAA)4 | 20 | 125100 | 125119 |
| chr1 | 32 | p5 | (AAAAG)3 | 15 | 128789 | 128803 |
| chr1 | 33 | p5 | (TTCTA)8 | 40 | 131743 | 131782 |
| chr1 | 34 | p3 | (TAT)4 | 12 | 139558 | 139569 |
| chr1 | 35 | p4 | (AGAA)3 | 12 | 143881 | 143892 |
| chr1 | 36 | p3 | (TTC)4 | 12 | 147092 | 147103 |
| chr1 | 37 | p2 | (CT)5 | 10 | 157177 | 157186 |
| chr1 | 38 | p1 | (T)10 | 10 | 158034 | 158043 |
| chr1 | 39 | p3 | (ATT)4 | 12 | 162585 | 162596 |
| chr1 | 40 | p4 | (GTGC)3 | 12 | 166466 | 166477 |
| chr1 | 41 | p4 | (TTCT)3 | 12 | 173770 | 173781 |
| chr1 | 42 | p4 | (AATC)3 | 12 | 173803 | 173814 |
| chr1 | 43 | p1 | (T)10 | 10 | 177079 | 177088 |
| chr1 | 44 | p4 | (TCGT)3 | 12 | 189199 | 189210 |
| chr1 | 45 | p2 | (TC)5 | 10 | 197796 | 197805 |
| chr1 | 46 | p2 | (TA)5 | 10 | 198911 | 198920 |
| chr1 | 47 | p5 | (TACTA)23 | 115 | 199108 | 199222 |
| chr1 | 48 | p1 | (T)11 | 11 | 200272 | 200282 |
| chr1 | 49 | p1 | (T)10 | 10 | 203270 | 203279 |
| chr1 | 50 | p2 | (GC)5 | 10 | 211259 | 211268 |
| chr1 | 51 | p4 | (CAAG)3 | 12 | 215501 | 215512 |
| chr1 | 52 | p2 | (AT)6 | 12 | 226628 | 226639 |
| chr1 | 53 | p4 | (ACCT)3 | 12 | 226811 | 226822 |
| chr1 | 54 | p4 | (TCTT)3 | 12 | 226950 | 226961 |
| chr1 | 55 | p4 | (CTAT)3 | 12 | 227423 | 227434 |
| chr1 | 56 | p3 | (TTA)4 | 12 | 229761 | 229772 |
| chr1 | 57 | p5 | (CTTTG)3 | 15 | 238968 | 238982 |
| chr1 | 58 | p5 | (TTTAT)24 | 120 | 244756 | 244875 |
| chr1 | 59 | p3 | (AAG)4 | 12 | 248219 | 248230 |
| chr1 | 60 | p4 | (GGGC)3 | 12 | 250687 | 250698 |
| chr1 | 61 | p6 | (GGGCTT)3 | 18 | 251099 | 251116 |
| chr1 | 62 | p4 | (ATAA)4 | 16 | 252532 | 252547 |
| chr1 | 63 | p1 | (A)13 | 13 | 266950 | 266962 |
| chr1 | 64 | p5 | (CAGTT)3 | 15 | 270305 | 270319 |
| chr1 | 65 | p4 | (TACT)3 | 12 | 271040 | 271051 |
| chr1 | 66 | p2 | (CT)5 | 10 | 271842 | 271851 |
| chr1 | 67 | p4 | (TTTC)3 | 12 | 278384 | 278395 |
| chr1 | 68 | p5 | (TTCTA)5 | 25 | 289940 | 289964 |
| chr1 | 69 | p2 | (TA)5 | 10 | 290497 | 290506 |
| chr1 | 70 | p5 | (TTCTA)5 | 25 | 296658 | 296682 |
| chr1 | 71 | p2 | (AG)5 | 10 | 299583 | 299592 |
| chr1 | 72 | p2 | (AT)5 | 10 | 303065 | 303074 |
| chr1 | 73 | p5 | (TTGCA)3 | 15 | 304658 | 304672 |
| chr1 | 74 | p4 | (GAGC)3 | 12 | 309556 | 309567 |
| chr1 | 75 | p4 | (AATG)3 | 12 | 314038 | 314049 |
| chr1 | 76 | p1 | (A)10 | 10 | 318012 | 318021 |
| chr1 | 77 | p1 | (C)10 | 10 | 319487 | 319496 |
| chr1 | 78 | p4 | (AATA)3 | 12 | 323020 | 323031 |
| chr1 | 79 | p2 | (AG)5 | 10 | 328304 | 328313 |
| chr1 | 80 | p4 | (GCCT)3 | 12 | 332945 | 332956 |
| chr1 | 81 | p4 | (TAGC)3 | 12 | 345030 | 345041 |
| chr1 | 82 | p1 | (T)10 | 10 | 347617 | 347626 |
| chr1 | 83 | p6 | (CTATTA)15 | 90 | 352324 | 352413 |
| chr1 | 84 | p6 | (ACTAAT)12 | 72 | 356521 | 356592 |
| chr1 | 85 | p4 | (GAAT)3 | 12 | 364865 | 364876 |
| chr1 | 86 | p5 | (ATTGA)3 | 15 | 365535 | 365549 |
| chr1 | 87 | p1 | (A)10 | 10 | 368937 | 368946 |
| chr1 | 88 | p1 | (T)10 | 10 | 374982 | 374991 |
| chr1 | 89 | p2 | (TC)5 | 10 | 386266 | 386275 |
| chr1 | 90 | p5 | (CTTTA)13 | 65 | 386890 | 386954 |
| chr1 | 91 | p4 | (GGTC)3 | 12 | 392768 | 392779 |
| chr1 | 92 | p5 | (TACTT)4 | 20 | 401077 | 401096 |
| chr1 | 93 | p4 | (ATTC)3 | 12 | 402722 | 402733 |

Note: p1: one-base repat; p2: two-base repeat; p3: three-base repeat; p4: four-base repeat; p5: five-base repeat; p6: six-base repeat.

**Supplementary table 5. Tandem repeats in *Cenchrus fungigraminus* mitochondrial genome**

| **NO.** | **Chr** | **Size** | **Copy** | **Repeat sequence** | **Percent Matches** | **Start** | **End** |
| --- | --- | --- | --- | --- | --- | --- | --- |
| 1 | chr1 | 5 | 14.2 | TATTG | 100 | 3727 | 3797 |
| 2 | chr1 | 5 | 24 | TTTAT | 100 | 17387 | 17506 |
| 3 | chr1 | 17 | 2 | ACTATACTAGACTAGA | 94 | 21379 | 21411 |
| 4 | chr1 | 5 | 6.2 | TTCTA | 92 | 40916 | 40947 |
| 5 | chr1 | 5 | 5 | TTCTA | 100 | 47634 | 47658 |
| 6 | chr1 | 18 | 2.1 | AATAAAAAGAAAGAAGAG | 95 | 64444 | 64481 |
| 7 | chr1 | 5 | 16 | ATTTG | 100 | 69869 | 69948 |
| 8 | chr1 | 23 | 2.3 | GGTCAAAGCGGCCAAAGACAAGCT | 78 | 85829 | 85883 |
| 9 | chr1 | 15 | 1.9 | GGTTGTGCTCGAAAC | 100 | 89305 | 89333 |
| 10 | chr1 | 5 | 17.2 | TTCTA | 100 | 105164 | 105249 |
| 11 | chr1 | 18 | 2.1 | AAAAATAGAGGTTGGTAC | 100 | 107280 | 107317 |
| 12 | chr1 | 6 | 11.8 | ATTTCG | 96 | 108413 | 108483 |
| 13 | chr1 | 5 | 6 | CCTAA | 100 | 116072 | 116101 |
| 14 | chr1 | 11 | 2.5 | TCTTTCTTATT | 100 | 118009 | 118036 |
| 15 | chr1 | 5 | 8 | TTCTA | 100 | 131743 | 131782 |
| 16 | chr1 | 24 | 2.3 | AAGTAAGTGTACCAAGAGTAGCAA | 90 | 138577 | 138632 |
| 17 | chr1 | 15 | 1.9 | AAATAAATACTAAGG | 100 | 141939 | 141967 |
| 18 | chr1 | 23 | 2.2 | CATCTCTGTAGATAAAGGAATAG | 89 | 144087 | 144137 |
| 19 | chr1 | 17 | 2 | CTCCATCTATACTATAA | 100 | 144740 | 144773 |
| 20 | chr1 | 15 | 2 | CATTATCTAGAGAAA | 93 | 165392 | 165421 |
| 21 | chr1 | 5 | 23.2 | TACTA | 100 | 199108 | 199223 |
| 22 | chr1 | 30 | 2 | AGTCAACACTACACTATGGACTTGCCTTTC | 87 | 211579 | 211639 |
| 23 | chr1 | 5 | 24 | TTTAT | 100 | 244756 | 244875 |
| 24 | chr1 | 17 | 2 | ACTATACTAGACTAGA | 94 | 248748 | 248780 |
| 25 | chr1 | 16 | 2.9 | TTTCACTGCCAATCTC | 84 | 256308 | 256354 |
| 26 | chr1 | 23 | 1.9 | ATGATAAGATAATAAGAAATCAA | 100 | 258748 | 258791 |
| 27 | chr1 | 19 | 3 | TATGAGGTCTAGCCAGTAG | 100 | 259124 | 259180 |
| 28 | chr1 | 27 | 4 | AATGAGTAGGTAGTTTGATCCTTTTGA | 98 | 276356 | 276463 |
| 29 | chr1 | 22 | 2 | TAGGAATTTGCCTATGATTTTT | 100 | 276869 | 276913 |
| 30 | chr1 | 5 | 6.2 | TTCTA | 92 | 289940 | 289971 |
| 31 | chr1 | 5 | 5 | TTCTA | 100 | 296658 | 296682 |
| 32 | chr1 | 6 | 15.7 | CTATTA | 100 | 352324 | 352417 |
| 33 | chr1 | 6 | 12.2 | ACTAAT | 100 | 356521 | 356593 |
| 34 | chr1 | 49 | 2 | CGAAGAGATAGAAGACAAGGTTCCTATCCCTATAGAAAGAAACTATCCCT | 98 | 365734 | 365834 |
| 35 | chr1 | 26 | 2.4 | CGAATAGTAGGAATAACTAGAATA | 88 | 383087 | 383145 |
| 36 | chr1 | 5 | 13 | CTTTA | 100 | 386890 | 386954 |
| 37 | chr1 | 15 | 2.1 | ACTTTAATAGTAGAA | 93 | 386949 | 386979 |
| 38 | chr1 | 13 | 2 | TCCTTCTTTAATT | 100 | 398016 | 398041 |

**Supplementary table 6. RNA editing type in *Cenchrus fungigraminus* mitochondrial genome**

| **Type** | **RNA-editing** | **Number** | **Percentage** |
| --- | --- | --- | --- |
| hydrophilic-hydrophilic | CAC (H) => TAC (Y) | 8 |  |
|  | CAT (H) => TAT (Y) | 18 |  |
|  | CGC (R) => TGC (C) | 5 |  |
|  | CGT (R) => TGT (C) | 25 |  |
|  | total | 56 | 10.28% |
| hydrophilic-hydrophobic | ACA (T) => ATA (I) | 9 |  |
|  | ACC (T) => ATC (I) | 8 |  |
|  | ACG (T) => ATG (M) | 8 |  |
|  | ACT (T) => ATT (I) | 9 |  |
|  | CGG (R) => TGG (W) | 31 |  |
|  | TCA (S) => TTA (L) | 74 |  |
|  | TCC (S) => TTC (F) | 38 |  |
|  | TCG (S) => TTG (L) | 42 |  |
|  | TCT (S) => TTT (F) | 45 |  |
|  | total | 264 | 48.44% |
| hydrophilic-stop | CAA (Q) => TAA (X) | 1 |  |
|  | CAG (Q) => TAG (X) | 1 |  |
|  | CGA (R) => TGA (X) | 1 |  |
|  | total | 3 | 0.55% |
| hydrophobic-hydrophilic | CCA (P) => TCA (S) | 8 |  |
|  | CCC (P) => TCC (S) | 15 |  |
|  | CCG (P) => TCG (S) | 5 |  |
|  | CCT (P) => TCT (S) | 17 |  |
|  | total | 45 | 8.26% |
| hydrophobic-hydrophobic | CCA (P) => CTA (L) | 43 |  |
|  | CCC (P) => CTC (L) | 11 |  |
|  | CCC (P) => TTC (F) | 4 |  |
|  | CCG (P) => CTG (L) | 20 |  |
|  | CCT (P) => CTT (L) | 24 |  |
|  | CCT (P) => TTT (F) | 6 |  |
|  | CTC (L) => TTC (F) | 13 |  |
|  | CTT (L) => TTT (F) | 32 |  |
|  | GCA (A) => GTA (V) | 9 |  |
|  | GCC (A) => GTC (V) | 3 |  |
|  | GCG (A) => GTG (V) | 6 |  |
|  | GCT (A) => GTT (V) | 6 |  |
|  | total | 177 | 32.48% |

**Supplementary table 7. Chloroplast DNA chloroplast transfer** **in *Cenchrus fungigraminus* mitochondrial genome**

| subject-mt | percentage of identical matches | length | number of mismatches | number of gap openings | start of alignment in query | end of alignment in query | start of alignment in subject | end of alignment in subject | expect value | bitscore | Gene (cp) |
| --- | --- | --- | --- | --- | --- | --- | --- | --- | --- | --- | --- |
| 1 | 99.956 | 2287 | 1 | 0 | 24639 | 26925 | 91590 | 89304 | 0 | 4218 | *rpoC1(partical:60.82%);rpoC2(partical:19.54%)* |
| 1 | 99.928 | 1387 | 0 | 1 | 99641 | 101027 | 190001 | 188616 | 0 | 2555 | *rrn23(partical:15.72%);rrn4.5;rrn5* |
| 1 | 99.928 | 1387 | 0 | 1 | 118212 | 119598 | 188616 | 190001 | 0 | 2555 | *rrn5;rrn4.5;rrn23(partical:15.72%)* |
| 1 | 99.571 | 1398 | 6 | 0 | 92291 | 93688 | 356533 | 355136 | 0 | 2549 | *trnV-GAC;rrn16(partical:14.42%)* |
| 1 | 99.571 | 1398 | 6 | 0 | 125551 | 126948 | 355136 | 356533 | 0 | 2549 | *rrn16(partical:14.48%);trnV-GAC* |
| 1 | 89.604 | 1895 | 118 | 30 | 48782 | 50647 | 107088 | 105244 | 0 | 2335 | *trnF-GAA;ndhJ;ndhK(partical:76.67%)* |
| 1 | 98.708 | 1316 | 14 | 2 | 91012 | 92324 | 357872 | 356557 | 0 | 2333 | *rps12(trans_splicing)(partical:57.61%)* |
| 1 | 98.708 | 1316 | 14 | 2 | 126915 | 128227 | 356557 | 357872 | 0 | 2333 | *rps12(trans_splicing)(partical:57.61%)* |
| 1 | 98.925 | 1209 | 8 | 1 | 75325 | 76528 | 184991 | 183783 | 0 | 2156 | *rpoA(partical:70.00%);rps11(partical:98.61%)* |
| 1 | 99.106 | 1007 | 4 | 1 | 81224 | 82230 | 62632 | 61631 | 0 | 1805 | *rps19(partical:68.79%);trnH-GUG;rpl2(partical:49.64%)* |
| 1 | 99.106 | 1007 | 4 | 1 | 137009 | 138015 | 61631 | 62632 | 0 | 1805 | *rpl2(partical:49.64%);trnH-GUG;rps19(partical:68.79%)* |
| 1 | 99.529 | 850 | 4 | 0 | 84615 | 85464 | 264210 | 263361 | 0 | 1548 | *ycf2(partical:25.76%)* |
| 1 | 99.529 | 850 | 4 | 0 | 133775 | 134624 | 263361 | 264210 | 0 | 1548 |  |
| 1 | 99.703 | 674 | 1 | 1 | 133108 | 133780 | 674 | 1 | 0 | 1232 | *ycf15* |
| 1 | 99.703 | 674 | 1 | 1 | 85459 | 86131 | 1 | 674 | 0 | 1232 | *ycf15* |
| 1 | 94.541 | 403 | 12 | 2 | 121845 | 122238 | 349460 | 349059 | ####### | 614 | *rrn23(partical:0.78%);trnA-UGC(partical:25.93%)* |
| 1 | 94.541 | 403 | 12 | 2 | 97001 | 97394 | 349059 | 349460 | ####### | 614 | *trnA-UGC(partical:25.93%);rrn23(partical:0.78%)* |
| 1 | 88.248 | 451 | 40 | 8 | 5342 | 5787 | 188082 | 188524 | ####### | 527 | *rps16(partical:20.55%)* |
| 1 | 91.247 | 377 | 31 | 2 | 62602 | 62976 | 171877 | 172253 | ####### | 512 | *psbF(partical:55.83%);psbE* |
| 1 | 89.59 | 317 | 14 | 9 | 96484 | 96795 | 26799 | 26497 | ####### | 385 | *trnA-UGC(partical:35.33%)* |
| 1 | 89.59 | 317 | 14 | 9 | 122444 | 122755 | 26497 | 26799 | ####### | 385 | *trnA-UGC(partical:35.33%)* |
| 1 | 93.694 | 222 | 14 | 0 | 56324 | 56545 | 146771 | 146550 | 2.23E-90 | 333 | *rbcL(partical:15.55%)* |
| 1 | 73.678 | 889 | 179 | 41 | 93687 | 94550 | 15174 | 14316 | 1.05E-78 | 294 | *rrn16(partical:57.95%)* |
| 1 | 73.678 | 889 | 179 | 41 | 124689 | 125552 | 14316 | 15174 | 1.05E-78 | 294 | *rrn16(partical:57.91%)* |
| 1 | 73.678 | 889 | 179 | 41 | 93687 | 94550 | 242543 | 241685 | 1.05E-78 | 294 | *rrn16(partical:57.95%)* |
| 1 | 73.678 | 889 | 179 | 41 | 124689 | 125552 | 241685 | 242543 | 1.05E-78 | 294 | *rrn16(partical:57.91%)* |
| 1 | 82.114 | 369 | 41 | 16 | 45758 | 46113 | 107890 | 107534 | 3.79E-78 | 292 | *trnS-GGA* |
| 1 | 79.494 | 395 | 58 | 11 | 77861 | 78245 | 321127 | 321508 | 3.84E-68 | 259 | *rpl14(partical:97.31%)* |
| 1 | 86.498 | 237 | 22 | 3 | 64918 | 65154 | 198294 | 198520 | 6.42E-66 | 252 | *trnP-UGG* |
| 1 | 96.429 | 140 | 2 | 1 | 34176 | 34315 | 194880 | 195016 | 1.08E-58 | 228 | *atpF(partical:10.19%)* |
| 1 | 93.243 | 148 | 10 | 0 | 24091 | 24238 | 124689 | 124542 | 6.52E-56 | 219 | *rpoC1(partical:7.21%)* |
| 1 | 100 | 87 | 0 | 0 | 117782 | 117868 | 93448 | 93362 | 1.11E-38 | 161 | *trnN-GUU* |
| 1 | 100 | 87 | 0 | 0 | 101371 | 101457 | 93362 | 93448 | 1.11E-38 | 161 | *trnN-GUU* |
| 1 | 88.525 | 122 | 12 | 2 | 56644 | 56764 | 215223 | 215103 | 3.12E-34 | 147 | *rbcL(partical:8.47%)* |
| 1 | 87.705 | 122 | 9 | 4 | 19277 | 19396 | 258788 | 258905 | 1.88E-31 | 137 | *trnC-GCA* |
| 1 | 96.203 | 79 | 3 | 0 | 64729 | 64807 | 198090 | 198168 | 3.14E-29 | 130 | *trnW-CCA* |
| 1 | 96.104 | 77 | 3 | 0 | 50642 | 50718 | 105169 | 105093 | 4.06E-28 | 126 | *ndhK(partical:10.27%)* |
| 1 | 93.421 | 76 | 4 | 1 | 52740 | 52814 | 373156 | 373081 | 1.14E-23 | 111 | *trnM-CAU* |
| 1 | 87 | 100 | 6 | 2 | 32626 | 32718 | 63894 | 63795 | 5.29E-22 | 106 |  |
| 1 | 94.737 | 57 | 3 | 0 | 28495 | 28551 | 4623 | 4679 | 5.33E-17 | 89.8 | *rpoC2(partical:1.29%)* |
| 1 | 82.474 | 97 | 17 | 0 | 97796 | 97892 | 45545 | 45449 | 6.89E-16 | 86.1 | *rrn23(partical:3.61%)* |
| 1 | 82.474 | 97 | 17 | 0 | 121347 | 121443 | 45449 | 45545 | 6.89E-16 | 86.1 | *rrn23(partical:3.61%)* |
| 1 | 82.474 | 97 | 17 | 0 | 97796 | 97892 | 294569 | 294473 | 6.89E-16 | 86.1 | *rrn23(partical:3.61%)* |
| 1 | 82.474 | 97 | 17 | 0 | 121347 | 121443 | 294473 | 294569 | 6.89E-16 | 86.1 | *rrn23(partical:3.61%)* |
| 1 | 97.619 | 42 | 1 | 0 | 31814 | 31855 | 220083 | 220042 | 5.37E-12 | 73.1 | *atpI(partical:5.65%)* |
| 1 | 79.121 | 91 | 14 | 5 | 7609 | 7697 | 107734 | 107821 | 1.50E-07 | 58.4 | *trnS-GCU* |

Mt: mitochondrion; cp: chloroplast

**Supplementary table 8. Codon and RSCU analysis in *Cenchrus fungigraminus* mitochondrial genome**

| Amino Acid | Symbol | Codon | No. | RSCU |
| --- | --- | --- | --- | --- |
| * | Ter | UAA | 14 | 1.3125 |
| * | Ter | UAG | 11 | 1.0312 |
| * | Ter | UGA | 7 | 0.6562 |
| A | Ala | GCA | 178 | 0.9875 |
| A | Ala | GCC | 159 | 0.8821 |
| A | Ala | GCG | 103 | 0.5714 |
| A | Ala | GCU | 281 | 1.5589 |
| C | Cys | UGC | 56 | 0.7671 |
| C | Cys | UGU | 90 | 1.2329 |
| D | Asp | GAC | 125 | 0.672 |
| D | Asp | GAU | 247 | 1.328 |
| E | Glu | GAA | 307 | 1.3554 |
| E | Glu | GAG | 146 | 0.6446 |
| F | Phe | UUC | 291 | 0.8423 |
| F | Phe | UUU | 400 | 1.1577 |
| G | Gly | GGA | 255 | 1.3821 |
| G | Gly | GGC | 105 | 0.5691 |
| G | Gly | GGG | 150 | 0.813 |
| G | Gly | GGU | 228 | 1.2358 |
| H | His | CAC | 63 | 0.4773 |
| H | His | CAU | 201 | 1.5227 |
| I | Ile | AUA | 226 | 0.8278 |
| I | Ile | AUC | 230 | 0.8425 |
| I | Ile | AUU | 363 | 1.3297 |
| K | Lys | AAA | 282 | 1.1775 |
| K | Lys | AAG | 197 | 0.8225 |
| L | Leu | CUA | 172 | 0.8843 |
| L | Leu | CUC | 135 | 0.6941 |
| L | Leu | CUG | 113 | 0.581 |
| L | Leu | CUU | 241 | 1.2391 |
| L | Leu | UUA | 275 | 1.4139 |
| L | Leu | UUG | 231 | 1.1877 |
| M | Met | AUG | 280 | 1 |
| N | Asn | AAC | 113 | 0.6532 |
| N | Asn | AAU | 233 | 1.3468 |
| P | Pro | CCA | 175 | 1.2324 |
| P | Pro | CCC | 117 | 0.8239 |
| P | Pro | CCG | 77 | 0.5423 |
| P | Pro | CCU | 199 | 1.4014 |
| Q | Gln | CAA | 233 | 1.5329 |
| Q | Gln | CAG | 71 | 0.4671 |
| R | Arg | AGA | 171 | 1.4657 |
| R | Arg | AGG | 105 | 0.9 |
| R | Arg | CGA | 139 | 1.1914 |
| R | Arg | CGC | 66 | 0.5657 |
| R | Arg | CGG | 79 | 0.6771 |
| R | Arg | CGU | 140 | 1.2 |
| S | Ser | AGC | 103 | 0.654 |
| S | Ser | AGU | 169 | 1.073 |
| S | Ser | UCA | 191 | 1.2127 |
| S | Ser | UCC | 159 | 1.0095 |
| S | Ser | UCG | 118 | 0.7492 |
| S | Ser | UCU | 205 | 1.3016 |
| T | Thr | ACA | 119 | 0.9032 |
| T | Thr | ACC | 141 | 1.0702 |
| T | Thr | ACG | 83 | 0.63 |
| T | Thr | ACU | 184 | 1.3966 |
| V | Val | GUA | 184 | 1.1518 |
| V | Val | GUC | 126 | 0.7887 |
| V | Val | GUG | 148 | 0.9264 |
| V | Val | GUU | 181 | 1.133 |
| W | Trp | UGG | 154 | 1 |
| Y | Tyr | UAC | 75 | 0.4702 |
| Y | Tyr | UAU | 244 | 1.5298 |

**Supplementary table 9. Mitochondrial genome and GC value in** **Poaceae**

| Sample | GC(%) | Size(kb) | NCBI accession number | Genus |
| --- | --- | --- | --- | --- |
| *Triticum aestivum* | 44.35 | 452.528 | AP008982 | *Triticum* |
| *Triticum timopheevii* | 44.35 | 443.419 | AP013106 | *Triticum* |
| *Oryza coarctata* | 43.07 | 491.065 | MG429050 | *Oryza* |
| *Oryza sativa Indica Group* | 43.92 | 637.692 | JF281153 | *Oryza* |
| *Oryza rufipogon* | 44.16 | 525.913 | AP012527 | *Oryza* |
| *Oryza sativa Japonica Group* | 43.85 | 490.52 | BA000029 | *Oryza* |
| *Oryza minuta* | 44.04 | 515.022 | KU176938 | *Oryza* |
| *Zea luxurians* | 43.93 | 539.368 | DQ645537 | *Zea* |
| *Zea perennis* | 43.88 | 570.354 | DQ645538 | *Zea* |
| *Zea mays subsp. parviglumis* | 43.88 | 680.603 | DQ645539 | *Zea* |
| *Zea mays subsp. mays* | 43.93 | 569.63 | NC_007982 | *Zea* |
| *Sorghum bicolor* | 43.73 | 468.628 | MZ506736.1 | *Sorghum* |
| *Sorghum bicolor subsp. drummondii* | 43.69 | 449.028 | DQ984518 | *Sorghum* |
| *Hordeum vulgare subsp. vulgare* | 44.23 | 525.599 | MN127968 | *Hordeum* |
| *Hordeum vulgare subsp. spontaneum* | 44.23 | 525.605 | MN127974 | *Hordeum* |
| *Lolium perenne* | 44.05 | 678.58 | JX999996 | *Lolium* |
| *Aegilops longissima* | 44.19 | 399.005 | KJ078648.1 | *Aegilops* |
| *Saccharum officinarum* | 43.82 | 445.482 | LC107874 | *Saccharum* |
| *Eleusine indica* | 43.3 | 520.691 | MF616338 | *Eleusine* |
| *Tripsacum dactyloides* | 43.93 | 704.1 | NC_008362 | *Tripsacum* |
| *Chrysopogon zizanioides* | 43.61 | 551.622 | NC_056367 | *Chrysopogon* |
| *Thinopyrum obtusiflorum* | 44.39 | 390.725 | OK120846.1 | *Thinopyrum* |
| *Elymus magellanicus* | 42.27 | 583.45 | OQ086977.1 | *Elymus* |
| *Agropyron cristatum* | 44.28 | 381.065 | PP503006 | *Agropyron* |
| *Aegilops speltoides var. ligustica* | 44.43 | 476.091 | AP013107 | *Aegilops* |
| *Cenchrus macrourus* | 43.98 | 404.398 | PQ720777 | *Cenchrus* |
| *Bambusa oldhamii* | 43.88 | 509.941 | EU365401 | *Bambusa* |

**Supplementary table 10. Gene composition variation in Poaceae**

| sample/gene | *atp1* | *atp4* | *atp6* | *atp8* | *atp9* | *ccmB* | *ccmC* | *ccmFc* | *ccmFn* | *cob* | *cox1* | *cox2* | *cox3* | *matR* | *mttB* | *nad1* | *nad2* | *nad3* | *nad4* | *nad4L* | *nad5* | *nad6* | *nad7* | *nad9* | *sdh3* | *sdh4* | *rps1* | *rps2* | *rps3* | *rps4* | *rps7* | *rps8* | *rps10* | *rps11* | *rps12* | *rps13* | *rps14* | *rps19* | *rpl2* | *rpl5* | *rpl6* | *rpl10* | *rpl16* |
| --- | --- | --- | --- | --- | --- | --- | --- | --- | --- | --- | --- | --- | --- | --- | --- | --- | --- | --- | --- | --- | --- | --- | --- | --- | --- | --- | --- | --- | --- | --- | --- | --- | --- | --- | --- | --- | --- | --- | --- | --- | --- | --- | --- |
| A1 | 1 | 1 | 1 | 1 | 1 | 1 | 1 | 1 | 1 | 1 | 1 | 1 | 1 | 1 | 1 | 1 | 1 | 1 | 1 | 1 | 1 | 1 | 1 | 1 | 0 | 0 | 1 | 1 | 1 | 1 | 1 | 0 | 0 | 0 | 1 | 1 | 0 | -1 | -1 | 1 | 0 | 0 | 1 |
| A2 | 1 | 1 | 1 | 1 | 1 | 1 | 1 | 1 | 1 | 1 | 1 | 1 | 1 | 1 | 1 | 1 | 1 | 1 | 1 | 1 | 1 | 1 | 1 | 1 | 0 | 0 | 1 | 1 | 1 | 1 | 1 | 0 | 0 | 0 | 1 | 1 | 0 | -1 | -1 | 1 | 0 | 0 | 1 |
| A3 | 1 | 1 | 1 | 1 | 1 | 1 | 1 | 1 | 1 | 1 | 1 | 1 | 1 | 1 | 1 | 1 | 1 | 1 | 1 | 1 | 1 | 1 | 1 | 1 | 0 | 0 | 1 | 1 | 1 | 1 | 1 | 0 | 0 | 0 | 1 | 1 | 0 | -1 | -1 | 1 | 0 | 0 | 1 |
| A4 | 1 | 1 | 0 | 1 | 1 | 1 | 0 | -1 | 1 | -1 | 1 | -1 | 1 | -1 | 1 | 0 | -1 | 0 | 1 | -1 | 0 | -1 | 1 | -1 | 0 | 0 | 1 | 1 | -1 | 1 | 1 | 0 | 0 | 0 | 1 | 0 | 0 | -1 | 0 | 1 | 0 | 0 | 0 |
| A5 | 1 | 1 | 1 | 1 | 1 | 1 | 1 | 1 | 1 | 1 | 1 | 1 | 1 | 1 | 1 | 1 | 1 | 1 | 1 | 1 | 1 | 1 | 1 | 1 | 0 | 0 | 1 | 1 | 1 | 1 | 1 | 0 | 0 | 0 | 1 | 1 | 0 | 0 | 0 | 1 | 0 | 0 | 1 |
| A6 | 1 | 1 | 1 | 1 | 1 | 1 | 1 | 1 | 1 | 1 | 1 | 1 | 1 | 1 | 1 | 1 | 1 | 1 | 1 | 1 | 1 | 1 | 1 | 1 | 0 | -1 | 1 | 1 | 1 | 1 | 1 | 0 | 0 | 0 | 1 | 1 | -1 | 1 | 0 | 0 | 0 | 0 | 1 |
| A7 | 1 | 1 | 1 | 1 | 1 | 1 | 1 | 1 | 1 | 1 | 1 | 1 | 1 | 1 | 1 | 1 | 1 | 1 | 1 | 1 | 1 | 1 | 1 | 1 | 0 | 0 | 1 | 1 | 1 | 1 | 1 | 0 | 0 | 0 | 1 | 1 | 0 | 0 | -1 | 1 | 0 | 0 | 1 |
| A8 | 1 | 1 | 1 | 1 | 1 | 1 | 1 | 1 | 1 | 1 | 1 | 1 | 1 | 1 | 1 | 1 | 1 | 1 | 1 | 1 | 1 | 1 | 1 | 1 | 0 | 0 | 1 | 1 | 1 | 1 | 1 | 0 | 0 | 0 | 1 | 1 | 0 | 0 | -1 | 1 | 0 | 0 | 1 |
| A9 | 1 | 1 | 1 | 1 | 1 | 1 | 1 | 1 | 1 | 1 | 1 | 1 | 1 | 0 | 1 | 1 | 1 | 1 | 1 | 0 | 1 | 1 | 1 | 1 | 0 | 0 | 1 | 1 | 1 | 1 | 1 | 1 | 0 | 1 | 1 | 1 | 0 | 1 | 0 | 0 | 0 | 0 | 0 |
| A10 | 1 | 1 | 1 | 1 | 1 | 1 | 1 | 1 | 1 | 1 | 1 | 1 | 1 | 1 | 1 | 1 | 1 | 1 | 1 | 1 | 1 | 1 | 1 | 1 | 0 | 0 | 1 | 1 | 1 | 1 | 1 | 0 | 0 | 0 | 1 | 1 | 1 | 0 | 0 | 1 | 0 | 0 | 1 |
| A11 | 1 | 1 | 1 | 1 | 1 | 1 | 1 | 1 | 1 | 1 | 1 | 1 | 1 | 1 | 1 | 1 | 1 | 1 | 1 | 1 | 1 | 1 | 1 | 1 | 0 | 0 | 1 | 1 | 1 | 1 | 1 | 0 | 0 | 0 | 1 | 1 | 0 | 1 | 0 | 1 | 0 | 0 | 1 |
| A12 | 1 | 1 | 1 | 1 | 1 | 1 | 1 | 1 | 1 | 1 | 1 | 1 | 1 | 1 | 1 | 1 | 1 | 1 | 1 | 1 | 1 | 1 | 1 | 1 | 0 | 0 | 1 | 1 | 1 | 1 | 1 | 0 | 0 | 0 | 1 | 1 | 0 | 0 | 0 | 0 | 0 | 0 | 1 |
| A13 | 1 | 1 | 1 | 1 | 1 | 1 | 1 | 1 | 1 | 1 | 1 | 1 | 1 | 1 | 1 | 1 | 1 | 1 | 1 | 1 | 1 | 1 | 1 | 1 | 0 | 0 | 1 | 1 | 1 | 1 | 1 | 0 | 0 | 0 | 1 | 1 | 0 | 0 | 0 | 0 | 0 | 0 | 1 |
| A14 | 1 | 1 | 1 | 1 | 1 | 1 | 1 | 1 | 1 | 1 | 1 | 1 | 1 | 1 | 1 | 1 | 1 | 1 | 1 | 1 | 1 | 1 | 1 | 1 | 0 | 0 | 1 | -1 | 1 | 1 | 1 | 0 | 0 | 0 | 1 | 1 | 0 | 0 | 0 | 0 | 0 | 0 | 1 |
| A15 | 1 | 1 | 1 | 1 | 1 | 1 | 1 | 1 | 1 | 1 | 1 | 1 | 1 | 1 | 0 | 1 | 1 | 1 | 1 | 1 | 1 | 1 | 1 | 1 | 0 | 0 | 1 | 0 | 1 | 1 | 1 | 0 | 0 | 0 | 1 | 1 | 0 | 0 | 0 | 0 | 0 | 0 | 1 |
| A16 | 1 | 1 | 1 | 1 | 1 | 1 | 1 | 1 | 1 | 1 | 1 | 1 | 1 | 1 | 1 | 1 | 1 | 1 | 1 | 1 | 1 | 1 | 1 | 1 | 0 | 0 | 1 | 0 | 1 | 1 | 1 | 0 | 0 | 0 | 1 | 1 | 0 | 0 | 0 | 0 | 0 | 0 | 1 |
| A17 | 1 | 1 | 1 | 1 | 1 | 1 | 1 | 1 | 1 | 1 | 1 | 1 | 1 | 1 | 1 | 1 | 1 | 1 | 1 | 1 | 1 | 1 | 1 | 1 | 0 | 0 | 1 | 1 | 1 | 1 | 1 | 0 | 0 | 0 | 1 | 1 | 0 | 0 | 1 | 0 | 0 | 0 | 1 |
| A18 | 1 | 1 | 1 | 1 | 1 | 1 | 1 | 1 | 1 | 1 | 1 | 1 | 1 | 1 | 1 | 1 | 1 | 1 | 1 | 1 | 1 | 1 | 1 | 1 | 0 | 0 | 1 | 1 | 1 | 1 | 1 | 0 | 0 | 0 | 1 | 1 | 0 | 0 | 0 | 0 | 0 | 0 | 1 |
| A19 | 1 | 1 | 1 | 1 | 1 | 1 | 1 | 1 | 1 | 1 | 1 | 1 | 1 | 1 | 1 | 1 | 1 | 1 | 1 | 1 | 1 | 1 | 1 | 1 | 0 | 0 | 1 | -1 | 1 | 1 | 1 | 0 | 0 | 0 | 1 | 1 | 0 | 0 | 0 | 0 | 0 | 0 | 1 |
| A20 | 1 | 1 | 1 | 1 | 1 | 1 | 1 | 1 | 1 | 1 | 1 | 1 | 1 | 1 | 1 | 1 | 1 | 1 | 1 | 1 | 1 | 1 | 1 | 1 | 0 | 0 | 1 | 1 | 1 | 1 | 1 | 0 | 0 | 0 | 1 | 1 | 0 | 0 | 0 | 0 | 0 | 0 | 0 |
| A21 | 1 | 1 | 1 | 1 | 1 | 1 | 1 | 1 | 1 | 1 | 1 | 1 | 1 | 1 | 1 | 1 | 1 | 1 | 1 | 1 | 1 | 1 | 1 | 1 | 0 | -1 | 1 | 1 | 1 | 1 | 1 | 0 | 0 | 0 | 1 | 1 | 0 | 0 | -1 | 0 | 0 | 0 | 1 |
| A22 | 1 | 1 | 1 | 1 | 1 | 1 | 1 | 1 | 1 | 1 | 1 | 1 | 1 | 1 | 1 | 1 | 1 | 1 | 1 | 1 | 1 | 1 | 1 | 1 | 0 | 0 | 1 | 1 | 1 | 1 | 1 | 0 | 0 | 0 | 1 | 1 | 0 | 1 | -1 | 0 | 0 | -1 | 1 |
| A23 | 0 | 0 | 0 | 0 | 1 | 1 | 1 | 1 | 1 | 1 | 1 | 1 | 1 | 1 | 0 | 0 | 0 | 1 | 1 | 1 | 0 | 1 | 0 | 1 | 0 | 0 | 1 | 0 | 1 | 1 | 1 | 0 | 0 | 0 | 1 | 1 | 0 | 1 | 1 | 1 | 0 | 0 | 1 |
| A24 | 1 | 1 | 1 | 1 | 1 | 1 | 1 | 1 | 1 | 1 | 1 | 1 | 1 | 1 | 0 | 1 | 1 | -1 | 1 | 1 | 1 | 1 | 1 | 1 | 0 | 0 | 1 | 1 | 1 | 1 | 1 | 0 | 0 | 0 | 1 | 1 | 0 | 1 | 1 | 1 | 0 | 0 | 0 |
| A25 | 1 | 1 | 1 | 1 | 1 | 1 | 1 | 1 | 1 | 1 | 1 | 1 | 1 | 1 | 0 | 1 | 1 | 1 | 1 | 1 | 1 | 1 | 1 | 1 | 0 | 0 | 1 | 1 | 1 | 1 | 1 | 0 | 0 | -1 | 1 | 1 | 0 | 1 | 1 | 1 | 0 | 0 | -1 |
| A26p | 1 | 1 | 1 | 1 | 1 | 1 | 1 | 1 | 1 | 1 | 1 | 1 | 1 | 1 | 0 | 1 | 1 | 1 | 1 | 1 | 1 | 1 | 1 | 1 | 0 | 0 | 1 | 1 | 1 | 1 | 1 | 0 | 0 | -1 | 1 | 1 | -1 | 1 | 1 | 1 | 0 | 0 | -1 |
| A27 | 1 | 1 | 1 | 1 | 1 | 1 | 1 | 1 | 1 | 1 | 1 | 1 | 1 | 1 | 0 | 1 | 1 | 1 | 1 | 1 | 1 | 1 | 1 | 1 | 0 | 0 | 1 | 1 | 1 | 1 | 1 | 0 | 0 | 0 | 1 | 1 | 0 | 1 | 1 | 1 | 0 | 0 | 1 |

Note: A1:*Triticum timopheevii*; A2:*Triticum aestivum*; A3:*Aegilops speltoides* var. ligustica; A4:*Aegilops longissimi*; A5:*Thinopyrum obtusiflorum*; A5:*Agropyron cristatum*; A7:*Hordeum vulgare* subsp. Vulgare; A8: *Hordeum vulgare* subsp. Spontaneum; A9:*Elymus magellanicus*; A10:*Lolium perenne*; A11:*Bambusa oldhamii*; A12:*Zea perennis*; A13:*Zea luxurians*; A14:*Zea mays* subsp. Parviglumis; A15:*Zea mays* subsp. mays; A16: *Tripsacum dactyloides*; A17:*Chrysopogon zizanioides*; A18:*Saccharum officinarum*; A19:*Sorghum bicolor*; A20: *Sorghum bicolor* subsp. drummondii; A21: *Cenchrus fungigraminus*; A22: *Eleusine indica*; A23: *Oryza minuta*; A24:*Oryza rufipogon*; A25: *Oryza sativa* Indica Group; A26:*Oryza sativa* Japonica Group; A27:*Oryza coarctata*

**Supplementary table 11. Ka/Ks value analysis in Poaceae**

| Kaks | Gene | Species 1 Vs Species 2 | Kaks | Gene | Species 1 Vs Species 2 |
| --- | --- | --- | --- | --- | --- |
| 0.128357 | atp1 | Cenchrus fungigraminus vs AP008982 | NA | nad4L | Cenchrus fungigraminus vs AP008982 |
| NA | atp1 | Cenchrus fungigraminus vs AP012527 | NA | nad4L | Cenchrus fungigraminus vs AP012527 |
| 0.128357 | atp1 | Cenchrus fungigraminus vs AP013106 | NA | nad4L | Cenchrus fungigraminus vs AP013106 |
| 0.125402 | atp1 | Cenchrus fungigraminus vs AP013107 | NA | nad4L | Cenchrus fungigraminus vs AP013107 |
| 0.138351 | atp1 | Cenchrus fungigraminus vs BA000029 | NA | nad4L | Cenchrus fungigraminus vs BA000029 |
| 0.222964 | atp1 | Cenchrus fungigraminus vs DQ645537 | 0 | nad4L | Cenchrus fungigraminus vs DQ645537 |
| 0.222964 | atp1 | Cenchrus fungigraminus vs DQ645538 | 0 | nad4L | Cenchrus fungigraminus vs DQ645538 |
| 0.197409 | atp1 | Cenchrus fungigraminus vs DQ645539 | 0 | nad4L | Cenchrus fungigraminus vs DQ645539 |
| NA | atp1 | Cenchrus fungigraminus vs DQ984518 | NA | nad4L | Cenchrus fungigraminus vs DQ984518 |
| 0.0836019 | atp1 | Cenchrus fungigraminus vs EU365401 | NA | nad4L | Cenchrus fungigraminus vs EU365401 |
| 0.138351 | atp1 | Cenchrus fungigraminus vs JF281153 | NA | nad4L | Cenchrus fungigraminus vs JF281153 |
| 0.10307 | atp1 | Cenchrus fungigraminus vs JX999996 | NA | nad4L | Cenchrus fungigraminus vs JX999996 |
| 0.141452 | atp1 | Cenchrus fungigraminus vs KJ078648 | NA | nad4L | Cenchrus fungigraminus vs KJ078648 |
| NA | atp1 | Cenchrus fungigraminus vs KU176938 | NA | nad4L | Cenchrus fungigraminus vs KU176938 |
| 0.13662 | atp1 | Cenchrus fungigraminus vs LC107874 | NA | nad4L | Cenchrus fungigraminus vs LC107874 |
| 0.101083 | atp1 | Cenchrus fungigraminus vs MF616338 | 0.236264 | nad4L | Cenchrus fungigraminus vs MF616338 |
| 0.144648 | atp1 | Cenchrus fungigraminus vs MG429050 | NA | nad4L | Cenchrus fungigraminus vs MG429050 |
| 0.128357 | atp1 | Cenchrus fungigraminus vs MN127968 | NA | nad4L | Cenchrus fungigraminus vs MN127968 |
| 0.128357 | atp1 | Cenchrus fungigraminus vs MN127974 | NA | nad4L | Cenchrus fungigraminus vs MN127974 |
| 0.313551 | atp1 | Cenchrus fungigraminus vs MZ506736 | NA | nad4L | Cenchrus fungigraminus vs MZ506736 |
| NA | atp1 | Cenchrus fungigraminus vs NC_007982 | 0 | nad4L | Cenchrus fungigraminus vs NC_007982 |
| NA | atp1 | Cenchrus fungigraminus vs NC_008362 | NA | nad4L | Cenchrus fungigraminus vs NC_008362 |
| 0.300799 | atp1 | Cenchrus fungigraminus vs NC_056367 | NA | nad4L | Cenchrus fungigraminus vs NC_056367 |
| 0.128357 | atp1 | Cenchrus fungigraminus vs OK120846 | NA | nad4L | Cenchrus fungigraminus vs OK120846 |
| 0.139791 | atp1 | Cenchrus fungigraminus vs OQ086977 | NA | nad4L | Cenchrus fungigraminus vs OQ086977 |
| 0.139791 | atp1 | Cenchrus fungigraminus vs PP503006 | NA | nad4L | Cenchrus fungigraminus vs PP503006 |
| 0.278223 | atp4 | Cenchrus fungigraminus vs AP008982 | 0.0308166 | nad5 | Cenchrus fungigraminus vs AP008982 |
| NA | atp4 | Cenchrus fungigraminus vs AP012527 | NA | nad5 | Cenchrus fungigraminus vs AP012527 |
| 0.278223 | atp4 | Cenchrus fungigraminus vs AP013106 | 0.0308166 | nad5 | Cenchrus fungigraminus vs AP013106 |
| 0.369746 | atp4 | Cenchrus fungigraminus vs AP013107 | 0.0308166 | nad5 | Cenchrus fungigraminus vs AP013107 |
| NA | atp4 | Cenchrus fungigraminus vs BA000029 | 0.0405492 | nad5 | Cenchrus fungigraminus vs BA000029 |
| 0.730199 | atp4 | Cenchrus fungigraminus vs DQ645537 | 0.232997 | nad5 | Cenchrus fungigraminus vs DQ645537 |
| 0.778881 | atp4 | Cenchrus fungigraminus vs DQ645538 | 0.189466 | nad5 | Cenchrus fungigraminus vs DQ645538 |
| 0.695926 | atp4 | Cenchrus fungigraminus vs DQ645539 | 0.189466 | nad5 | Cenchrus fungigraminus vs DQ645539 |
| 0.660944 | atp4 | Cenchrus fungigraminus vs DQ984518 | 0.378295 | nad5 | Cenchrus fungigraminus vs DQ984518 |
| 0.113346 | atp4 | Cenchrus fungigraminus vs EU365401 | 0.0753963 | nad5 | Cenchrus fungigraminus vs EU365401 |
| 0.379511 | atp4 | Cenchrus fungigraminus vs JF281153 | 0.0405492 | nad5 | Cenchrus fungigraminus vs JF281153 |
| 0.471606 | atp4 | Cenchrus fungigraminus vs JX999996 | 0.0632027 | nad5 | Cenchrus fungigraminus vs JX999996 |
| NA | atp4 | Cenchrus fungigraminus vs KJ078648 | NA | nad5 | Cenchrus fungigraminus vs KJ078648 |
| NA | atp4 | Cenchrus fungigraminus vs KU176938 | NA | nad5 | Cenchrus fungigraminus vs KU176938 |
| 0.661541 | atp4 | Cenchrus fungigraminus vs LC107874 | 0.228257 | nad5 | Cenchrus fungigraminus vs LC107874 |
| 0.49544 | atp4 | Cenchrus fungigraminus vs MF616338 | 0.0828268 | nad5 | Cenchrus fungigraminus vs MF616338 |
| 0.411692 | atp4 | Cenchrus fungigraminus vs MG429050 | 0.116105 | nad5 | Cenchrus fungigraminus vs MG429050 |
| 0.276849 | atp4 | Cenchrus fungigraminus vs MN127968 | 0.0308166 | nad5 | Cenchrus fungigraminus vs MN127968 |
| 0.276849 | atp4 | Cenchrus fungigraminus vs MN127974 | 0.290228 | nad5 | Cenchrus fungigraminus vs MN127974 |
| 0.660944 | atp4 | Cenchrus fungigraminus vs MZ506736 | 0.228257 | nad5 | Cenchrus fungigraminus vs MZ506736 |
| 0.79162 | atp4 | Cenchrus fungigraminus vs NC_007982 | 0.189466 | nad5 | Cenchrus fungigraminus vs NC_007982 |
| 0.798024 | atp4 | Cenchrus fungigraminus vs NC_008362 | 0.233254 | nad5 | Cenchrus fungigraminus vs NC_008362 |
| 1.47729 | atp4 | Cenchrus fungigraminus vs NC_056367 | 0.343049 | nad5 | Cenchrus fungigraminus vs NC_056367 |
| 0.369746 | atp4 | Cenchrus fungigraminus vs OK120846 | 0.0308166 | nad5 | Cenchrus fungigraminus vs OK120846 |
| 0.18405 | atp4 | Cenchrus fungigraminus vs OQ086977 | 0.0308166 | nad5 | Cenchrus fungigraminus vs OQ086977 |
| 0.136833 | atp4 | Cenchrus fungigraminus vs PP503006 | 0 | nad5 | Cenchrus fungigraminus vs PP503006 |
| 0.488846 | atp6 | Cenchrus fungigraminus vs AP008982 | 0.923119 | nad6 | Cenchrus fungigraminus vs AP008982 |
| NA | atp6 | Cenchrus fungigraminus vs AP012527 | NA | nad6 | Cenchrus fungigraminus vs AP012527 |
| 0.488846 | atp6 | Cenchrus fungigraminus vs AP013106 | 0.923119 | nad6 | Cenchrus fungigraminus vs AP013106 |
| 0.458599 | atp6 | Cenchrus fungigraminus vs AP013107 | 0.923119 | nad6 | Cenchrus fungigraminus vs AP013107 |
| 0.487885 | atp6 | Cenchrus fungigraminus vs BA000029 | 0.493885 | nad6 | Cenchrus fungigraminus vs BA000029 |
| 0.471511 | atp6 | Cenchrus fungigraminus vs DQ645537 | 0.53035 | nad6 | Cenchrus fungigraminus vs DQ645537 |
| NA | atp6 | Cenchrus fungigraminus vs DQ645538 | 0.673443 | nad6 | Cenchrus fungigraminus vs DQ645538 |
| 0.480728 | atp6 | Cenchrus fungigraminus vs DQ645539 | 0.682187 | nad6 | Cenchrus fungigraminus vs DQ645539 |
| 0.464433 | atp6 | Cenchrus fungigraminus vs DQ984518 | 0.527123 | nad6 | Cenchrus fungigraminus vs DQ984518 |
| 0.350447 | atp6 | Cenchrus fungigraminus vs EU365401 | 0.778408 | nad6 | Cenchrus fungigraminus vs EU365401 |
| 0.487885 | atp6 | Cenchrus fungigraminus vs JF281153 | 0.510187 | nad6 | Cenchrus fungigraminus vs JF281153 |
| 0.256975 | atp6 | Cenchrus fungigraminus vs JX999996 | 0.454598 | nad6 | Cenchrus fungigraminus vs JX999996 |
| NA | atp6 | Cenchrus fungigraminus vs KJ078648 | NA | nad6 | Cenchrus fungigraminus vs KJ078648 |
| NA | atp6 | Cenchrus fungigraminus vs KU176938 | 0.463482 | nad6 | Cenchrus fungigraminus vs KU176938 |
| 0.460876 | atp6 | Cenchrus fungigraminus vs LC107874 | 0.517973 | nad6 | Cenchrus fungigraminus vs LC107874 |
| 0.224504 | atp6 | Cenchrus fungigraminus vs MF616338 | 0.516727 | nad6 | Cenchrus fungigraminus vs MF616338 |
| 0.314668 | atp6 | Cenchrus fungigraminus vs MG429050 | 0.480969 | nad6 | Cenchrus fungigraminus vs MG429050 |
| 0.426853 | atp6 | Cenchrus fungigraminus vs MN127968 | 0.494783 | nad6 | Cenchrus fungigraminus vs MN127968 |
| 0.426853 | atp6 | Cenchrus fungigraminus vs MN127974 | 0.494783 | nad6 | Cenchrus fungigraminus vs MN127974 |
| 0.464433 | atp6 | Cenchrus fungigraminus vs MZ506736 | 0.524858 | nad6 | Cenchrus fungigraminus vs MZ506736 |
| 0.480728 | atp6 | Cenchrus fungigraminus vs NC_007982 | 0.682187 | nad6 | Cenchrus fungigraminus vs NC_007982 |
| NA | atp6 | Cenchrus fungigraminus vs NC_008362 | 0.50137 | nad6 | Cenchrus fungigraminus vs NC_008362 |
| 0.381593 | atp6 | Cenchrus fungigraminus vs NC_056367 | 0.524159 | nad6 | Cenchrus fungigraminus vs NC_056367 |
| 0.450459 | atp6 | Cenchrus fungigraminus vs OK120846 | 0.364621 | nad6 | Cenchrus fungigraminus vs OK120846 |
| 0.437891 | atp6 | Cenchrus fungigraminus vs OQ086977 | 0.637801 | nad6 | Cenchrus fungigraminus vs OQ086977 |
| 0.492304 | atp6 | Cenchrus fungigraminus vs PP503006 | 0.42499 | nad6 | Cenchrus fungigraminus vs PP503006 |
| NA | atp8 | Cenchrus fungigraminus vs AP008982 | 0.06112 | nad7 | Cenchrus fungigraminus vs AP008982 |
| NA | atp8 | Cenchrus fungigraminus vs AP012527 | 0 | nad7 | Cenchrus fungigraminus vs AP012527 |
| 0.388877 | atp8 | Cenchrus fungigraminus vs AP013106 | 0.06112 | nad7 | Cenchrus fungigraminus vs AP013106 |
| 0.388877 | atp8 | Cenchrus fungigraminus vs AP013107 | 0.06112 | nad7 | Cenchrus fungigraminus vs AP013107 |
| NA | atp8 | Cenchrus fungigraminus vs BA000029 | 1.81806 | nad7 | Cenchrus fungigraminus vs BA000029 |
| 0.325114 | atp8 | Cenchrus fungigraminus vs DQ645537 | 0.376734 | nad7 | Cenchrus fungigraminus vs DQ645537 |
| 0.325114 | atp8 | Cenchrus fungigraminus vs DQ645538 | 0 | nad7 | Cenchrus fungigraminus vs DQ645538 |
| 0.325114 | atp8 | Cenchrus fungigraminus vs DQ645539 | 0.247563 | nad7 | Cenchrus fungigraminus vs DQ645539 |
| 0.220886 | atp8 | Cenchrus fungigraminus vs DQ984518 | 0 | nad7 | Cenchrus fungigraminus vs DQ984518 |
| 0.337698 | atp8 | Cenchrus fungigraminus vs EU365401 | 0 | nad7 | Cenchrus fungigraminus vs EU365401 |
| 0.405124 | atp8 | Cenchrus fungigraminus vs JF281153 | 0 | nad7 | Cenchrus fungigraminus vs JF281153 |
| 0.325613 | atp8 | Cenchrus fungigraminus vs JX999996 | 0 | nad7 | Cenchrus fungigraminus vs JX999996 |
| 0.320612 | atp8 | Cenchrus fungigraminus vs KJ078648 | 0.06112 | nad7 | Cenchrus fungigraminus vs KJ078648 |
| NA | atp8 | Cenchrus fungigraminus vs KU176938 | NA | nad7 | Cenchrus fungigraminus vs KU176938 |
| 0.187352 | atp8 | Cenchrus fungigraminus vs LC107874 | 0 | nad7 | Cenchrus fungigraminus vs LC107874 |
| 0.324798 | atp8 | Cenchrus fungigraminus vs MF616338 | 0.816382 | nad7 | Cenchrus fungigraminus vs MF616338 |
| 0.405124 | atp8 | Cenchrus fungigraminus vs MG429050 | 0.184408 | nad7 | Cenchrus fungigraminus vs MG429050 |
| 0.318452 | atp8 | Cenchrus fungigraminus vs MN127968 | 0.0817951 | nad7 | Cenchrus fungigraminus vs MN127968 |
| 0.318452 | atp8 | Cenchrus fungigraminus vs MN127974 | 0.0817951 | nad7 | Cenchrus fungigraminus vs MN127974 |
| 0.220886 | atp8 | Cenchrus fungigraminus vs MZ506736 | 0 | nad7 | Cenchrus fungigraminus vs MZ506736 |
| 0.325114 | atp8 | Cenchrus fungigraminus vs NC_007982 | 0.247563 | nad7 | Cenchrus fungigraminus vs NC_007982 |
| 0.18878 | atp8 | Cenchrus fungigraminus vs NC_008362 | 0 | nad7 | Cenchrus fungigraminus vs NC_008362 |
| 0.220886 | atp8 | Cenchrus fungigraminus vs NC_056367 | 0 | nad7 | Cenchrus fungigraminus vs NC_056367 |
| 0.461165 | atp8 | Cenchrus fungigraminus vs OK120846 | 0.0817951 | nad7 | Cenchrus fungigraminus vs OK120846 |
| 0.490083 | atp8 | Cenchrus fungigraminus vs OQ086977 | 0.0817951 | nad7 | Cenchrus fungigraminus vs OQ086977 |
| 0.486604 | atp8 | Cenchrus fungigraminus vs PP503006 | 0.0817951 | nad7 | Cenchrus fungigraminus vs PP503006 |
| 0.167456 | atp9 | Cenchrus fungigraminus vs AP008982 | 0 | nad9 | Cenchrus fungigraminus vs AP008982 |
| NA | atp9 | Cenchrus fungigraminus vs AP012527 | NA | nad9 | Cenchrus fungigraminus vs AP012527 |
| 0.167456 | atp9 | Cenchrus fungigraminus vs AP013106 | 0 | nad9 | Cenchrus fungigraminus vs AP013106 |
| 0.167456 | atp9 | Cenchrus fungigraminus vs AP013107 | 0 | nad9 | Cenchrus fungigraminus vs AP013107 |
| 0.284974 | atp9 | Cenchrus fungigraminus vs BA000029 | 0.0976369 | nad9 | Cenchrus fungigraminus vs BA000029 |
| 0.139517 | atp9 | Cenchrus fungigraminus vs DQ645537 | 0.894155 | nad9 | Cenchrus fungigraminus vs DQ645537 |
| 0.139517 | atp9 | Cenchrus fungigraminus vs DQ645538 | 0.721913 | nad9 | Cenchrus fungigraminus vs DQ645538 |
| 0.139517 | atp9 | Cenchrus fungigraminus vs DQ645539 | 0.721913 | nad9 | Cenchrus fungigraminus vs DQ645539 |
| 0.0742712 | atp9 | Cenchrus fungigraminus vs DQ984518 | 0.721913 | nad9 | Cenchrus fungigraminus vs DQ984518 |
| 0.135056 | atp9 | Cenchrus fungigraminus vs EU365401 | 0.0976369 | nad9 | Cenchrus fungigraminus vs EU365401 |
| 0.284974 | atp9 | Cenchrus fungigraminus vs JF281153 | 0.0976369 | nad9 | Cenchrus fungigraminus vs JF281153 |
| 0.16205 | atp9 | Cenchrus fungigraminus vs JX999996 | 0.178675 | nad9 | Cenchrus fungigraminus vs JX999996 |
| 0.167456 | atp9 | Cenchrus fungigraminus vs KJ078648 | NA | nad9 | Cenchrus fungigraminus vs KJ078648 |
| 0.304824 | atp9 | Cenchrus fungigraminus vs KU176938 | 0.0976369 | nad9 | Cenchrus fungigraminus vs KU176938 |
| 0.130688 | atp9 | Cenchrus fungigraminus vs LC107874 | 0.297648 | nad9 | Cenchrus fungigraminus vs LC107874 |
| 0.0510669 | atp9 | Cenchrus fungigraminus vs MF616338 | 0.133102 | nad9 | Cenchrus fungigraminus vs MF616338 |
| 0.431495 | atp9 | Cenchrus fungigraminus vs MG429050 | 0.0976369 | nad9 | Cenchrus fungigraminus vs MG429050 |
| 0.167456 | atp9 | Cenchrus fungigraminus vs MN127968 | 0 | nad9 | Cenchrus fungigraminus vs MN127968 |
| 0.167456 | atp9 | Cenchrus fungigraminus vs MN127974 | 0 | nad9 | Cenchrus fungigraminus vs MN127974 |
| 0.0742712 | atp9 | Cenchrus fungigraminus vs MZ506736 | 0.721913 | nad9 | Cenchrus fungigraminus vs MZ506736 |
| 0.139517 | atp9 | Cenchrus fungigraminus vs NC_007982 | 0.721913 | nad9 | Cenchrus fungigraminus vs NC_007982 |
| 0.109005 | atp9 | Cenchrus fungigraminus vs NC_008362 | NA | nad9 | Cenchrus fungigraminus vs NC_008362 |
| 0.130688 | atp9 | Cenchrus fungigraminus vs NC_056367 | 0.377765 | nad9 | Cenchrus fungigraminus vs NC_056367 |
| 0.167456 | atp9 | Cenchrus fungigraminus vs OK120846 | 0 | nad9 | Cenchrus fungigraminus vs OK120846 |
| 0.167456 | atp9 | Cenchrus fungigraminus vs OQ086977 | 0 | nad9 | Cenchrus fungigraminus vs OQ086977 |
| 0.256706 | atp9 | Cenchrus fungigraminus vs PP503006 | 0 | nad9 | Cenchrus fungigraminus vs PP503006 |
| 2.48012 | ccmB | Cenchrus fungigraminus vs AP008982 | 0.410665 | rpl16 | Cenchrus fungigraminus vs AP008982 |
| NA | ccmB | Cenchrus fungigraminus vs AP012527 | NA | rpl16 | Cenchrus fungigraminus vs AP012527 |
| 2.48012 | ccmB | Cenchrus fungigraminus vs AP013106 | 0.410665 | rpl16 | Cenchrus fungigraminus vs AP013106 |
| 2.48012 | ccmB | Cenchrus fungigraminus vs AP013107 | 0.410665 | rpl16 | Cenchrus fungigraminus vs AP013107 |
| 3.15815 | ccmB | Cenchrus fungigraminus vs BA000029 | NA | rpl16 | Cenchrus fungigraminus vs BA000029 |
| 1.44484 | ccmB | Cenchrus fungigraminus vs DQ645537 | 0.25729 | rpl16 | Cenchrus fungigraminus vs DQ645537 |
| 1.44484 | ccmB | Cenchrus fungigraminus vs DQ645538 | 0.25729 | rpl16 | Cenchrus fungigraminus vs DQ645538 |
| 1.34405 | ccmB | Cenchrus fungigraminus vs DQ645539 | 0.191457 | rpl16 | Cenchrus fungigraminus vs DQ645539 |
| 1.34405 | ccmB | Cenchrus fungigraminus vs DQ984518 | 0.170182 | rpl16 | Cenchrus fungigraminus vs DQ984518 |
| 1.06046 | ccmB | Cenchrus fungigraminus vs EU365401 | 0.649241 | rpl16 | Cenchrus fungigraminus vs EU365401 |
| 3.15815 | ccmB | Cenchrus fungigraminus vs JF281153 | NA | rpl16 | Cenchrus fungigraminus vs JF281153 |
| 2.48012 | ccmB | Cenchrus fungigraminus vs JX999996 | 0.400477 | rpl16 | Cenchrus fungigraminus vs JX999996 |
| 2.48012 | ccmB | Cenchrus fungigraminus vs KJ078648 | NA | rpl16 | Cenchrus fungigraminus vs KJ078648 |
| 3.22429 | ccmB | Cenchrus fungigraminus vs KU176938 | 0.501427 | rpl16 | Cenchrus fungigraminus vs KU176938 |
| 1.34405 | ccmB | Cenchrus fungigraminus vs LC107874 | 0.152954 | rpl16 | Cenchrus fungigraminus vs LC107874 |
| 3.07311 | ccmB | Cenchrus fungigraminus vs MF616338 | 0.556118 | rpl16 | Cenchrus fungigraminus vs MF616338 |
| 3.15815 | ccmB | Cenchrus fungigraminus vs MG429050 | 0.501427 | rpl16 | Cenchrus fungigraminus vs MG429050 |
| 2.48012 | ccmB | Cenchrus fungigraminus vs MN127968 | 0.326826 | rpl16 | Cenchrus fungigraminus vs MN127968 |
| 2.48012 | ccmB | Cenchrus fungigraminus vs MN127974 | 0.326826 | rpl16 | Cenchrus fungigraminus vs MN127974 |
| 1.34405 | ccmB | Cenchrus fungigraminus vs MZ506736 | NA | rpl16 | Cenchrus fungigraminus vs MZ506736 |
| 1.34405 | ccmB | Cenchrus fungigraminus vs NC_007982 | 0.191457 | rpl16 | Cenchrus fungigraminus vs NC_007982 |
| NA | ccmB | Cenchrus fungigraminus vs NC_008362 | 0.474136 | rpl16 | Cenchrus fungigraminus vs NC_008362 |
| 1.91142 | ccmB | Cenchrus fungigraminus vs NC_056367 | 0.170182 | rpl16 | Cenchrus fungigraminus vs NC_056367 |
| 2.48012 | ccmB | Cenchrus fungigraminus vs OK120846 | 0.410665 | rpl16 | Cenchrus fungigraminus vs OK120846 |
| 2.48012 | ccmB | Cenchrus fungigraminus vs OQ086977 | 0.410665 | rpl16 | Cenchrus fungigraminus vs OQ086977 |
| 1.26759 | ccmB | Cenchrus fungigraminus vs PP503006 | 0.410665 | rpl16 | Cenchrus fungigraminus vs PP503006 |
| NA | ccmC | Cenchrus fungigraminus vs AP008982 | 0.604577 | rps1 | Cenchrus fungigraminus vs AP008982 |
| NA | ccmC | Cenchrus fungigraminus vs AP012527 | NA | rps1 | Cenchrus fungigraminus vs AP012527 |
| NA | ccmC | Cenchrus fungigraminus vs AP013106 | 0.534731 | rps1 | Cenchrus fungigraminus vs AP013106 |
| NA | ccmC | Cenchrus fungigraminus vs AP013107 | 0.604577 | rps1 | Cenchrus fungigraminus vs AP013107 |
| 0.144386 | ccmC | Cenchrus fungigraminus vs BA000029 | 0.555452 | rps1 | Cenchrus fungigraminus vs BA000029 |
| NA | ccmC | Cenchrus fungigraminus vs DQ645537 | 0.590076 | rps1 | Cenchrus fungigraminus vs DQ645537 |
| NA | ccmC | Cenchrus fungigraminus vs DQ645538 | 0.603043 | rps1 | Cenchrus fungigraminus vs DQ645538 |
| NA | ccmC | Cenchrus fungigraminus vs DQ645539 | 0.603043 | rps1 | Cenchrus fungigraminus vs DQ645539 |
| NA | ccmC | Cenchrus fungigraminus vs DQ984518 | 0.472686 | rps1 | Cenchrus fungigraminus vs DQ984518 |
| NA | ccmC | Cenchrus fungigraminus vs EU365401 | 0.457953 | rps1 | Cenchrus fungigraminus vs EU365401 |
| 0.144386 | ccmC | Cenchrus fungigraminus vs JF281153 | 0.555452 | rps1 | Cenchrus fungigraminus vs JF281153 |
| 0.33777 | ccmC | Cenchrus fungigraminus vs JX999996 | 0.418488 | rps1 | Cenchrus fungigraminus vs JX999996 |
| NA | ccmC | Cenchrus fungigraminus vs KJ078648 | 0.491218 | rps1 | Cenchrus fungigraminus vs KJ078648 |
| 0.333244 | ccmC | Cenchrus fungigraminus vs KU176938 | 0.522124 | rps1 | Cenchrus fungigraminus vs KU176938 |
| NA | ccmC | Cenchrus fungigraminus vs LC107874 | 0.472686 | rps1 | Cenchrus fungigraminus vs LC107874 |
| NA | ccmC | Cenchrus fungigraminus vs MF616338 | 0.604783 | rps1 | Cenchrus fungigraminus vs MF616338 |
| 0.333244 | ccmC | Cenchrus fungigraminus vs MG429050 | 0.555452 | rps1 | Cenchrus fungigraminus vs MG429050 |
| NA | ccmC | Cenchrus fungigraminus vs MN127968 | 0.491218 | rps1 | Cenchrus fungigraminus vs MN127968 |
| NA | ccmC | Cenchrus fungigraminus vs MN127974 | 0.491218 | rps1 | Cenchrus fungigraminus vs MN127974 |
| NA | ccmC | Cenchrus fungigraminus vs MZ506736 | 0.472686 | rps1 | Cenchrus fungigraminus vs MZ506736 |
| NA | ccmC | Cenchrus fungigraminus vs NC_007982 | 0.603043 | rps1 | Cenchrus fungigraminus vs NC_007982 |
| 0.538653 | ccmC | Cenchrus fungigraminus vs NC_008362 | NA | rps1 | Cenchrus fungigraminus vs NC_008362 |
| NA | ccmC | Cenchrus fungigraminus vs NC_056367 | 0.472686 | rps1 | Cenchrus fungigraminus vs NC_056367 |
| NA | ccmC | Cenchrus fungigraminus vs OK120846 | 0.476695 | rps1 | Cenchrus fungigraminus vs OK120846 |
| 1.16727 | ccmC | Cenchrus fungigraminus vs OQ086977 | 0.393408 | rps1 | Cenchrus fungigraminus vs OQ086977 |
| NA | ccmC | Cenchrus fungigraminus vs PP503006 | 0.3413 | rps1 | Cenchrus fungigraminus vs PP503006 |
| 0.694591 | ccmFc | Cenchrus fungigraminus vs AP008982 | 0 | rps12 | Cenchrus fungigraminus vs AP008982 |
| NA | ccmFc | Cenchrus fungigraminus vs AP012527 | 0.126356 | rps12 | Cenchrus fungigraminus vs AP012527 |
| 0.800152 | ccmFc | Cenchrus fungigraminus vs AP013106 | 0 | rps12 | Cenchrus fungigraminus vs AP013106 |
| 0.800152 | ccmFc | Cenchrus fungigraminus vs AP013107 | 0 | rps12 | Cenchrus fungigraminus vs AP013107 |
| 0.774129 | ccmFc | Cenchrus fungigraminus vs BA000029 | 0.126356 | rps12 | Cenchrus fungigraminus vs BA000029 |
| NA | ccmFc | Cenchrus fungigraminus vs DQ645537 | 0 | rps12 | Cenchrus fungigraminus vs DQ645537 |
| 0.840226 | ccmFc | Cenchrus fungigraminus vs DQ645538 | 0 | rps12 | Cenchrus fungigraminus vs DQ645538 |
| 0.840226 | ccmFc | Cenchrus fungigraminus vs DQ645539 | 0 | rps12 | Cenchrus fungigraminus vs DQ645539 |
| 1.8624 | ccmFc | Cenchrus fungigraminus vs DQ984518 | NA | rps12 | Cenchrus fungigraminus vs DQ984518 |
| 0.640933 | ccmFc | Cenchrus fungigraminus vs EU365401 | NA | rps12 | Cenchrus fungigraminus vs EU365401 |
| 1.03842 | ccmFc | Cenchrus fungigraminus vs JF281153 | 0.126356 | rps12 | Cenchrus fungigraminus vs JF281153 |
| 0.638996 | ccmFc | Cenchrus fungigraminus vs JX999996 | 0 | rps12 | Cenchrus fungigraminus vs JX999996 |
| NA | ccmFc | Cenchrus fungigraminus vs KJ078648 | 0 | rps12 | Cenchrus fungigraminus vs KJ078648 |
| 0.561921 | ccmFc | Cenchrus fungigraminus vs KU176938 | 0.126356 | rps12 | Cenchrus fungigraminus vs KU176938 |
| 1.8624 | ccmFc | Cenchrus fungigraminus vs LC107874 | NA | rps12 | Cenchrus fungigraminus vs LC107874 |
| 0.457944 | ccmFc | Cenchrus fungigraminus vs MF616338 | 0 | rps12 | Cenchrus fungigraminus vs MF616338 |
| 1.03842 | ccmFc | Cenchrus fungigraminus vs MG429050 | 0.126356 | rps12 | Cenchrus fungigraminus vs MG429050 |
| 0.673754 | ccmFc | Cenchrus fungigraminus vs MN127968 | 0 | rps12 | Cenchrus fungigraminus vs MN127968 |
| 0.673754 | ccmFc | Cenchrus fungigraminus vs MN127974 | 0 | rps12 | Cenchrus fungigraminus vs MN127974 |
| 1.8624 | ccmFc | Cenchrus fungigraminus vs MZ506736 | NA | rps12 | Cenchrus fungigraminus vs MZ506736 |
| 0.840226 | ccmFc | Cenchrus fungigraminus vs NC_007982 | 0 | rps12 | Cenchrus fungigraminus vs NC_007982 |
| NA | ccmFc | Cenchrus fungigraminus vs NC_008362 | 0 | rps12 | Cenchrus fungigraminus vs NC_008362 |
| 1.8624 | ccmFc | Cenchrus fungigraminus vs NC_056367 | NA | rps12 | Cenchrus fungigraminus vs NC_056367 |
| 0.694591 | ccmFc | Cenchrus fungigraminus vs OK120846 | 0 | rps12 | Cenchrus fungigraminus vs OK120846 |
| 0.834752 | ccmFc | Cenchrus fungigraminus vs OQ086977 | 0 | rps12 | Cenchrus fungigraminus vs OQ086977 |
| 1.10622 | ccmFc | Cenchrus fungigraminus vs PP503006 | 0 | rps12 | Cenchrus fungigraminus vs PP503006 |
| 0.521342 | ccmFn | Cenchrus fungigraminus vs AP008982 | 0.430343 | rps13 | Cenchrus fungigraminus vs AP008982 |
| NA | ccmFn | Cenchrus fungigraminus vs AP012527 | NA | rps13 | Cenchrus fungigraminus vs AP012527 |
| 0.521342 | ccmFn | Cenchrus fungigraminus vs AP013106 | 0.430343 | rps13 | Cenchrus fungigraminus vs AP013106 |
| 0.521342 | ccmFn | Cenchrus fungigraminus vs AP013107 | 0.430343 | rps13 | Cenchrus fungigraminus vs AP013107 |
| 0.813555 | ccmFn | Cenchrus fungigraminus vs BA000029 | 1.11592 | rps13 | Cenchrus fungigraminus vs BA000029 |
| 0.594429 | ccmFn | Cenchrus fungigraminus vs DQ645537 | 0.438371 | rps13 | Cenchrus fungigraminus vs DQ645537 |
| 0.543099 | ccmFn | Cenchrus fungigraminus vs DQ645538 | 0.438371 | rps13 | Cenchrus fungigraminus vs DQ645538 |
| 0.944125 | ccmFn | Cenchrus fungigraminus vs DQ645539 | 0.438371 | rps13 | Cenchrus fungigraminus vs DQ645539 |
| 0.6437 | ccmFn | Cenchrus fungigraminus vs DQ984518 | 0.438371 | rps13 | Cenchrus fungigraminus vs DQ984518 |
| 0.718506 | ccmFn | Cenchrus fungigraminus vs EU365401 | 0.880033 | rps13 | Cenchrus fungigraminus vs EU365401 |
| 0.692058 | ccmFn | Cenchrus fungigraminus vs JF281153 | 1.11592 | rps13 | Cenchrus fungigraminus vs JF281153 |
| 0.906223 | ccmFn | Cenchrus fungigraminus vs JX999996 | 0.347609 | rps13 | Cenchrus fungigraminus vs JX999996 |
| 0.56765 | ccmFn | Cenchrus fungigraminus vs KJ078648 | 0.880033 | rps13 | Cenchrus fungigraminus vs KJ078648 |
| 0.635239 | ccmFn | Cenchrus fungigraminus vs KU176938 | 1.11592 | rps13 | Cenchrus fungigraminus vs KU176938 |
| 0.755194 | ccmFn | Cenchrus fungigraminus vs LC107874 | 0.438371 | rps13 | Cenchrus fungigraminus vs LC107874 |
| 0.504756 | ccmFn | Cenchrus fungigraminus vs MF616338 | NA | rps13 | Cenchrus fungigraminus vs MF616338 |
| 0.776576 | ccmFn | Cenchrus fungigraminus vs MG429050 | 1.11592 | rps13 | Cenchrus fungigraminus vs MG429050 |
| 0.645017 | ccmFn | Cenchrus fungigraminus vs MN127968 | 0.880033 | rps13 | Cenchrus fungigraminus vs MN127968 |
| 0.645017 | ccmFn | Cenchrus fungigraminus vs MN127974 | 0.880033 | rps13 | Cenchrus fungigraminus vs MN127974 |
| 0.594319 | ccmFn | Cenchrus fungigraminus vs MZ506736 | 0.438371 | rps13 | Cenchrus fungigraminus vs MZ506736 |
| 0.944125 | ccmFn | Cenchrus fungigraminus vs NC_007982 | 0.438371 | rps13 | Cenchrus fungigraminus vs NC_007982 |
| NA | ccmFn | Cenchrus fungigraminus vs NC_008362 | 0.438371 | rps13 | Cenchrus fungigraminus vs NC_008362 |
| 1.15395 | ccmFn | Cenchrus fungigraminus vs NC_056367 | 0.438371 | rps13 | Cenchrus fungigraminus vs NC_056367 |
| 0.569233 | ccmFn | Cenchrus fungigraminus vs OK120846 | 0.880033 | rps13 | Cenchrus fungigraminus vs OK120846 |
| 0.645017 | ccmFn | Cenchrus fungigraminus vs OQ086977 | 0.880033 | rps13 | Cenchrus fungigraminus vs OQ086977 |
| 0.593598 | ccmFn | Cenchrus fungigraminus vs PP503006 | 0.880033 | rps13 | Cenchrus fungigraminus vs PP503006 |
| 0.348282 | cob | Cenchrus fungigraminus vs AP008982 | 0.584758 | rps2 | Cenchrus fungigraminus vs AP008982 |
| NA | cob | Cenchrus fungigraminus vs AP012527 | NA | rps2 | Cenchrus fungigraminus vs AP012527 |
| NA | cob | Cenchrus fungigraminus vs AP013106 | 0.584663 | rps2 | Cenchrus fungigraminus vs AP013106 |
| 0.290538 | cob | Cenchrus fungigraminus vs AP013107 | 0.58059 | rps2 | Cenchrus fungigraminus vs AP013107 |
| 0.252243 | cob | Cenchrus fungigraminus vs BA000029 | 0.553161 | rps2 | Cenchrus fungigraminus vs BA000029 |
| 0.2352 | cob | Cenchrus fungigraminus vs DQ645537 | 0.569898 | rps2 | Cenchrus fungigraminus vs DQ645537 |
| 0.2352 | cob | Cenchrus fungigraminus vs DQ645538 | NA | rps2 | Cenchrus fungigraminus vs DQ645538 |
| 0.2352 | cob | Cenchrus fungigraminus vs DQ645539 | NA | rps2 | Cenchrus fungigraminus vs DQ645539 |
| NA | cob | Cenchrus fungigraminus vs DQ984518 | NA | rps2 | Cenchrus fungigraminus vs DQ984518 |
| 0.447287 | cob | Cenchrus fungigraminus vs EU365401 | 0.466788 | rps2 | Cenchrus fungigraminus vs EU365401 |
| 0.252243 | cob | Cenchrus fungigraminus vs JF281153 | 0.550552 | rps2 | Cenchrus fungigraminus vs JF281153 |
| 0.402632 | cob | Cenchrus fungigraminus vs JX999996 | 0.382189 | rps2 | Cenchrus fungigraminus vs JX999996 |
| NA | cob | Cenchrus fungigraminus vs KJ078648 | 0.591148 | rps2 | Cenchrus fungigraminus vs KJ078648 |
| 0.252243 | cob | Cenchrus fungigraminus vs KU176938 | NA | rps2 | Cenchrus fungigraminus vs KU176938 |
| 0.236663 | cob | Cenchrus fungigraminus vs LC107874 | 0.509008 | rps2 | Cenchrus fungigraminus vs LC107874 |
| 0.317447 | cob | Cenchrus fungigraminus vs MF616338 | 0.51618 | rps2 | Cenchrus fungigraminus vs MF616338 |
| 0.270208 | cob | Cenchrus fungigraminus vs MG429050 | 0.528165 | rps2 | Cenchrus fungigraminus vs MG429050 |
| 0.348282 | cob | Cenchrus fungigraminus vs MN127968 | 0.50732 | rps2 | Cenchrus fungigraminus vs MN127968 |
| 0.348282 | cob | Cenchrus fungigraminus vs MN127974 | 0.50732 | rps2 | Cenchrus fungigraminus vs MN127974 |
| 0.473074 | cob | Cenchrus fungigraminus vs MZ506736 | 0.592711 | rps2 | Cenchrus fungigraminus vs MZ506736 |
| 0.2352 | cob | Cenchrus fungigraminus vs NC_007982 | NA | rps2 | Cenchrus fungigraminus vs NC_007982 |
| 0.284803 | cob | Cenchrus fungigraminus vs NC_008362 | NA | rps2 | Cenchrus fungigraminus vs NC_008362 |
| 0.473074 | cob | Cenchrus fungigraminus vs NC_056367 | 0.542575 | rps2 | Cenchrus fungigraminus vs NC_056367 |
| 0.348282 | cob | Cenchrus fungigraminus vs OK120846 | 0.566561 | rps2 | Cenchrus fungigraminus vs OK120846 |
| 0.348282 | cob | Cenchrus fungigraminus vs OQ086977 | 0.566545 | rps2 | Cenchrus fungigraminus vs OQ086977 |
| 0.348282 | cob | Cenchrus fungigraminus vs PP503006 | 0.53407 | rps2 | Cenchrus fungigraminus vs PP503006 |
| 0.134115 | cox1 | Cenchrus fungigraminus vs AP008982 | 0.639583 | rps3 | Cenchrus fungigraminus vs AP008982 |
| 0.1146 | cox1 | Cenchrus fungigraminus vs AP012527 | 0.56135 | rps3 | Cenchrus fungigraminus vs AP012527 |
| 0.146666 | cox1 | Cenchrus fungigraminus vs AP013106 | 0.639583 | rps3 | Cenchrus fungigraminus vs AP013106 |
| 0.134115 | cox1 | Cenchrus fungigraminus vs AP013107 | 0.639583 | rps3 | Cenchrus fungigraminus vs AP013107 |
| 0.681687 | cox1 | Cenchrus fungigraminus vs BA000029 | 0.530688 | rps3 | Cenchrus fungigraminus vs BA000029 |
| 0.259348 | cox1 | Cenchrus fungigraminus vs DQ645537 | 0.437786 | rps3 | Cenchrus fungigraminus vs DQ645537 |
| 0.290255 | cox1 | Cenchrus fungigraminus vs DQ645538 | 0.56381 | rps3 | Cenchrus fungigraminus vs DQ645538 |
| 0.326181 | cox1 | Cenchrus fungigraminus vs DQ645539 | 0.60906 | rps3 | Cenchrus fungigraminus vs DQ645539 |
| 0.206604 | cox1 | Cenchrus fungigraminus vs DQ984518 | 0.607498 | rps3 | Cenchrus fungigraminus vs DQ984518 |
| 0.175691 | cox1 | Cenchrus fungigraminus vs EU365401 | 0.461817 | rps3 | Cenchrus fungigraminus vs EU365401 |
| 0.1146 | cox1 | Cenchrus fungigraminus vs JF281153 | 0.56135 | rps3 | Cenchrus fungigraminus vs JF281153 |
| 0.531441 | cox1 | Cenchrus fungigraminus vs JX999996 | 0.808951 | rps3 | Cenchrus fungigraminus vs JX999996 |
| 0.134115 | cox1 | Cenchrus fungigraminus vs KJ078648 | NA | rps3 | Cenchrus fungigraminus vs KJ078648 |
| 0.1146 | cox1 | Cenchrus fungigraminus vs KU176938 | 0.596316 | rps3 | Cenchrus fungigraminus vs KU176938 |
| 0.291531 | cox1 | Cenchrus fungigraminus vs LC107874 | 0.451491 | rps3 | Cenchrus fungigraminus vs LC107874 |
| 0.576551 | cox1 | Cenchrus fungigraminus vs MF616338 | 0.48743 | rps3 | Cenchrus fungigraminus vs MF616338 |
| 0.1146 | cox1 | Cenchrus fungigraminus vs MG429050 | 0.515116 | rps3 | Cenchrus fungigraminus vs MG429050 |
| 0.090758 | cox1 | Cenchrus fungigraminus vs MN127968 | 0.677145 | rps3 | Cenchrus fungigraminus vs MN127968 |
| 0.090758 | cox1 | Cenchrus fungigraminus vs MN127974 | 0.677145 | rps3 | Cenchrus fungigraminus vs MN127974 |
| 0.206604 | cox1 | Cenchrus fungigraminus vs MZ506736 | 0.607498 | rps3 | Cenchrus fungigraminus vs MZ506736 |
| 0.326181 | cox1 | Cenchrus fungigraminus vs NC_007982 | 0.60906 | rps3 | Cenchrus fungigraminus vs NC_007982 |
| 0.585039 | cox1 | Cenchrus fungigraminus vs NC_008362 | 0.553901 | rps3 | Cenchrus fungigraminus vs NC_008362 |
| 0.344207 | cox1 | Cenchrus fungigraminus vs NC_056367 | 0.479907 | rps3 | Cenchrus fungigraminus vs NC_056367 |
| 0.133749 | cox1 | Cenchrus fungigraminus vs OK120846 | 0.678727 | rps3 | Cenchrus fungigraminus vs OK120846 |
| 0.138432 | cox1 | Cenchrus fungigraminus vs OQ086977 | 0.697112 | rps3 | Cenchrus fungigraminus vs OQ086977 |
| 0.114437 | cox1 | Cenchrus fungigraminus vs PP503006 | 0.678727 | rps3 | Cenchrus fungigraminus vs PP503006 |
| 0.164889 | cox2 | Cenchrus fungigraminus vs AP008982 | 0.856209 | rps4 | Cenchrus fungigraminus vs AP008982 |
| 0.0791691 | cox2 | Cenchrus fungigraminus vs AP012527 | NA | rps4 | Cenchrus fungigraminus vs AP012527 |
| 0.164889 | cox2 | Cenchrus fungigraminus vs AP013106 | 0.778272 | rps4 | Cenchrus fungigraminus vs AP013106 |
| 0.164889 | cox2 | Cenchrus fungigraminus vs AP013107 | 0.778272 | rps4 | Cenchrus fungigraminus vs AP013107 |
| 0.0452433 | cox2 | Cenchrus fungigraminus vs BA000029 | 1.76787 | rps4 | Cenchrus fungigraminus vs BA000029 |
| 0.114963 | cox2 | Cenchrus fungigraminus vs DQ645537 | 0.60019 | rps4 | Cenchrus fungigraminus vs DQ645537 |
| 0 | cox2 | Cenchrus fungigraminus vs DQ645538 | 0.517338 | rps4 | Cenchrus fungigraminus vs DQ645538 |
| 0 | cox2 | Cenchrus fungigraminus vs DQ645539 | NA | rps4 | Cenchrus fungigraminus vs DQ645539 |
| 0.116283 | cox2 | Cenchrus fungigraminus vs DQ984518 | 0.865232 | rps4 | Cenchrus fungigraminus vs DQ984518 |
| 0.0907043 | cox2 | Cenchrus fungigraminus vs EU365401 | 1.06327 | rps4 | Cenchrus fungigraminus vs EU365401 |
| 0.0452433 | cox2 | Cenchrus fungigraminus vs JF281153 | 1.76787 | rps4 | Cenchrus fungigraminus vs JF281153 |
| 0.0639008 | cox2 | Cenchrus fungigraminus vs JX999996 | 1.00626 | rps4 | Cenchrus fungigraminus vs JX999996 |
| NA | cox2 | Cenchrus fungigraminus vs KJ078648 | 0.777755 | rps4 | Cenchrus fungigraminus vs KJ078648 |
| 0.0826152 | cox2 | Cenchrus fungigraminus vs KU176938 | 1.77736 | rps4 | Cenchrus fungigraminus vs KU176938 |
| 0 | cox2 | Cenchrus fungigraminus vs LC107874 | 0.795755 | rps4 | Cenchrus fungigraminus vs LC107874 |
| 0.200956 | cox2 | Cenchrus fungigraminus vs MF616338 | 0.793619 | rps4 | Cenchrus fungigraminus vs MF616338 |
| 0.0826152 | cox2 | Cenchrus fungigraminus vs MG429050 | 1.35389 | rps4 | Cenchrus fungigraminus vs MG429050 |
| 0.0792819 | cox2 | Cenchrus fungigraminus vs MN127968 | 0.73326 | rps4 | Cenchrus fungigraminus vs MN127968 |
| 0.0792819 | cox2 | Cenchrus fungigraminus vs MN127974 | 0.73326 | rps4 | Cenchrus fungigraminus vs MN127974 |
| 0.116283 | cox2 | Cenchrus fungigraminus vs MZ506736 | 0.865232 | rps4 | Cenchrus fungigraminus vs MZ506736 |
| 0 | cox2 | Cenchrus fungigraminus vs NC_007982 | 0.517338 | rps4 | Cenchrus fungigraminus vs NC_007982 |
| 0 | cox2 | Cenchrus fungigraminus vs NC_008362 | NA | rps4 | Cenchrus fungigraminus vs NC_008362 |
| 0 | cox2 | Cenchrus fungigraminus vs NC_056367 | 0.748783 | rps4 | Cenchrus fungigraminus vs NC_056367 |
| 0.13954 | cox2 | Cenchrus fungigraminus vs OK120846 | 0.774183 | rps4 | Cenchrus fungigraminus vs OK120846 |
| 0.110531 | cox2 | Cenchrus fungigraminus vs OQ086977 | 0.706305 | rps4 | Cenchrus fungigraminus vs OQ086977 |
| 0.0792819 | cox2 | Cenchrus fungigraminus vs PP503006 | 0.774183 | rps4 | Cenchrus fungigraminus vs PP503006 |
| 0.224209 | cox3 | Cenchrus fungigraminus vs AP008982 | 0.744368 | rps7 | Cenchrus fungigraminus vs AP008982 |
| NA | cox3 | Cenchrus fungigraminus vs AP012527 | NA | rps7 | Cenchrus fungigraminus vs AP012527 |
| 0.0863848 | cox3 | Cenchrus fungigraminus vs AP013106 | 0.744368 | rps7 | Cenchrus fungigraminus vs AP013106 |
| 0.225859 | cox3 | Cenchrus fungigraminus vs AP013107 | 0.744368 | rps7 | Cenchrus fungigraminus vs AP013107 |
| 0 | cox3 | Cenchrus fungigraminus vs BA000029 | 4.17047 | rps7 | Cenchrus fungigraminus vs BA000029 |
| 0.0887922 | cox3 | Cenchrus fungigraminus vs DQ645537 | NA | rps7 | Cenchrus fungigraminus vs DQ645537 |
| 0.0887922 | cox3 | Cenchrus fungigraminus vs DQ645538 | NA | rps7 | Cenchrus fungigraminus vs DQ645538 |
| 0.0887922 | cox3 | Cenchrus fungigraminus vs DQ645539 | NA | rps7 | Cenchrus fungigraminus vs DQ645539 |
| 0.0887922 | cox3 | Cenchrus fungigraminus vs DQ984518 | NA | rps7 | Cenchrus fungigraminus vs DQ984518 |
| 0 | cox3 | Cenchrus fungigraminus vs EU365401 | NA | rps7 | Cenchrus fungigraminus vs EU365401 |
| 0.0887922 | cox3 | Cenchrus fungigraminus vs JF281153 | 4.17047 | rps7 | Cenchrus fungigraminus vs JF281153 |
| 0 | cox3 | Cenchrus fungigraminus vs JX999996 | NA | rps7 | Cenchrus fungigraminus vs JX999996 |
| 0 | cox3 | Cenchrus fungigraminus vs KJ078648 | 0.744368 | rps7 | Cenchrus fungigraminus vs KJ078648 |
| 0 | cox3 | Cenchrus fungigraminus vs KU176938 | 4.17047 | rps7 | Cenchrus fungigraminus vs KU176938 |
| 0.0887922 | cox3 | Cenchrus fungigraminus vs LC107874 | NA | rps7 | Cenchrus fungigraminus vs LC107874 |
| 0 | cox3 | Cenchrus fungigraminus vs MF616338 | NA | rps7 | Cenchrus fungigraminus vs MF616338 |
| 0 | cox3 | Cenchrus fungigraminus vs MG429050 | 4.17047 | rps7 | Cenchrus fungigraminus vs MG429050 |
| 0.0886727 | cox3 | Cenchrus fungigraminus vs MN127968 | 0.744368 | rps7 | Cenchrus fungigraminus vs MN127968 |
| 0.0886727 | cox3 | Cenchrus fungigraminus vs MN127974 | 0.744368 | rps7 | Cenchrus fungigraminus vs MN127974 |
| 0.0887922 | cox3 | Cenchrus fungigraminus vs MZ506736 | 1.07467 | rps7 | Cenchrus fungigraminus vs MZ506736 |
| 0.0887922 | cox3 | Cenchrus fungigraminus vs NC_007982 | NA | rps7 | Cenchrus fungigraminus vs NC_007982 |
| 0.0887922 | cox3 | Cenchrus fungigraminus vs NC_008362 | NA | rps7 | Cenchrus fungigraminus vs NC_008362 |
| 0.0887922 | cox3 | Cenchrus fungigraminus vs NC_056367 | NA | rps7 | Cenchrus fungigraminus vs NC_056367 |
| 0 | cox3 | Cenchrus fungigraminus vs OK120846 | 0.744368 | rps7 | Cenchrus fungigraminus vs OK120846 |
| 0 | cox3 | Cenchrus fungigraminus vs OQ086977 | 0.744368 | rps7 | Cenchrus fungigraminus vs OQ086977 |
| 0 | cox3 | Cenchrus fungigraminus vs PP503006 | 0.744368 | rps7 | Cenchrus fungigraminus vs PP503006 |
| 0.475687 | matR | Cenchrus fungigraminus vs AP008982 | 2.23873 | nad2 | Cenchrus fungigraminus vs AP008982 |
| 0.579255 | matR | Cenchrus fungigraminus vs AP012527 | 2.55545 | nad2 | Cenchrus fungigraminus vs AP012527 |
| 0.543573 | matR | Cenchrus fungigraminus vs AP013106 | 2.23873 | nad2 | Cenchrus fungigraminus vs AP013106 |
| 0.543573 | matR | Cenchrus fungigraminus vs AP013107 | 2.35873 | nad2 | Cenchrus fungigraminus vs AP013107 |
| 0.469202 | matR | Cenchrus fungigraminus vs BA000029 | NA | nad2 | Cenchrus fungigraminus vs BA000029 |
| 0.848078 | matR | Cenchrus fungigraminus vs DQ645537 | NA | nad2 | Cenchrus fungigraminus vs DQ645537 |
| 0.765788 | matR | Cenchrus fungigraminus vs DQ645538 | NA | nad2 | Cenchrus fungigraminus vs DQ645538 |
| 0.765788 | matR | Cenchrus fungigraminus vs DQ645539 | NA | nad2 | Cenchrus fungigraminus vs DQ645539 |
| 0.531205 | matR | Cenchrus fungigraminus vs DQ984518 | NA | nad2 | Cenchrus fungigraminus vs DQ984518 |
| 0.648939 | matR | Cenchrus fungigraminus vs EU365401 | 1.22664 | nad2 | Cenchrus fungigraminus vs EU365401 |
| 0.579255 | matR | Cenchrus fungigraminus vs JF281153 | 2.55545 | nad2 | Cenchrus fungigraminus vs JF281153 |
| 0.550423 | matR | Cenchrus fungigraminus vs JX999996 | 0.959364 | nad2 | Cenchrus fungigraminus vs JX999996 |
| NA | matR | Cenchrus fungigraminus vs KJ078648 | NA | nad2 | Cenchrus fungigraminus vs KJ078648 |
| 0.666858 | matR | Cenchrus fungigraminus vs KU176938 | NA | nad2 | Cenchrus fungigraminus vs KU176938 |
| 0.531205 | matR | Cenchrus fungigraminus vs LC107874 | 0.73332 | nad2 | Cenchrus fungigraminus vs LC107874 |
| 1.16149 | matR | Cenchrus fungigraminus vs MF616338 | 0.823226 | nad2 | Cenchrus fungigraminus vs MF616338 |
| 0.623515 | matR | Cenchrus fungigraminus vs MG429050 | 0.882882 | nad2 | Cenchrus fungigraminus vs MG429050 |
| 0.591439 | matR | Cenchrus fungigraminus vs MN127968 | 1.72593 | nad2 | Cenchrus fungigraminus vs MN127968 |
| 0.591439 | matR | Cenchrus fungigraminus vs MN127974 | 1.72593 | nad2 | Cenchrus fungigraminus vs MN127974 |
| 0.531205 | matR | Cenchrus fungigraminus vs MZ506736 | NA | nad2 | Cenchrus fungigraminus vs MZ506736 |
| 0.765788 | matR | Cenchrus fungigraminus vs NC_007982 | NA | nad2 | Cenchrus fungigraminus vs NC_007982 |
| NA | matR | Cenchrus fungigraminus vs NC_008362 | NA | nad2 | Cenchrus fungigraminus vs NC_008362 |
| 0.531205 | matR | Cenchrus fungigraminus vs NC_056367 | 0.243577 | nad2 | Cenchrus fungigraminus vs NC_056367 |
| 0.543573 | matR | Cenchrus fungigraminus vs OK120846 | 2.23873 | nad2 | Cenchrus fungigraminus vs OK120846 |
| NA | matR | Cenchrus fungigraminus vs OQ086977 | 1.72593 | nad2 | Cenchrus fungigraminus vs OQ086977 |
| 0.591439 | matR | Cenchrus fungigraminus vs PP503006 | 2.23873 | nad2 | Cenchrus fungigraminus vs PP503006 |
| 0.28685 | mttB | Cenchrus fungigraminus vs AP008982 | NA | nad3 | Cenchrus fungigraminus vs AP008982 |
| NA | mttB | Cenchrus fungigraminus vs AP012527 | NA | nad3 | Cenchrus fungigraminus vs AP012527 |
| 0.28685 | mttB | Cenchrus fungigraminus vs AP013106 | NA | nad3 | Cenchrus fungigraminus vs AP013106 |
| 0.28685 | mttB | Cenchrus fungigraminus vs AP013107 | NA | nad3 | Cenchrus fungigraminus vs AP013107 |
| NA | mttB | Cenchrus fungigraminus vs BA000029 | NA | nad3 | Cenchrus fungigraminus vs BA000029 |
| NA | mttB | Cenchrus fungigraminus vs DQ645537 | 0 | nad3 | Cenchrus fungigraminus vs DQ645537 |
| NA | mttB | Cenchrus fungigraminus vs DQ645538 | 0 | nad3 | Cenchrus fungigraminus vs DQ645538 |
| NA | mttB | Cenchrus fungigraminus vs DQ645539 | 0 | nad3 | Cenchrus fungigraminus vs DQ645539 |
| NA | mttB | Cenchrus fungigraminus vs DQ984518 | NA | nad3 | Cenchrus fungigraminus vs DQ984518 |
| 0.217608 | mttB | Cenchrus fungigraminus vs EU365401 | NA | nad3 | Cenchrus fungigraminus vs EU365401 |
| NA | mttB | Cenchrus fungigraminus vs JF281153 | NA | nad3 | Cenchrus fungigraminus vs JF281153 |
| 0.203231 | mttB | Cenchrus fungigraminus vs JX999996 | NA | nad3 | Cenchrus fungigraminus vs JX999996 |
| 0.28685 | mttB | Cenchrus fungigraminus vs KJ078648 | NA | nad3 | Cenchrus fungigraminus vs KJ078648 |
| NA | mttB | Cenchrus fungigraminus vs KU176938 | NA | nad3 | Cenchrus fungigraminus vs KU176938 |
| NA | mttB | Cenchrus fungigraminus vs LC107874 | NA | nad3 | Cenchrus fungigraminus vs LC107874 |
| 0 | mttB | Cenchrus fungigraminus vs MF616338 | NA | nad3 | Cenchrus fungigraminus vs MF616338 |
| NA | mttB | Cenchrus fungigraminus vs MG429050 | NA | nad3 | Cenchrus fungigraminus vs MG429050 |
| 0.28685 | mttB | Cenchrus fungigraminus vs MN127968 | NA | nad3 | Cenchrus fungigraminus vs MN127968 |
| 0.28685 | mttB | Cenchrus fungigraminus vs MN127974 | NA | nad3 | Cenchrus fungigraminus vs MN127974 |
| NA | mttB | Cenchrus fungigraminus vs MZ506736 | NA | nad3 | Cenchrus fungigraminus vs MZ506736 |
| NA | mttB | Cenchrus fungigraminus vs NC_007982 | 0 | nad3 | Cenchrus fungigraminus vs NC_007982 |
| NA | mttB | Cenchrus fungigraminus vs NC_008362 | 0 | nad3 | Cenchrus fungigraminus vs NC_008362 |
| NA | mttB | Cenchrus fungigraminus vs NC_056367 | NA | nad3 | Cenchrus fungigraminus vs NC_056367 |
| 0.28685 | mttB | Cenchrus fungigraminus vs OK120846 | NA | nad3 | Cenchrus fungigraminus vs OK120846 |
| 0.208216 | mttB | Cenchrus fungigraminus vs OQ086977 | NA | nad3 | Cenchrus fungigraminus vs OQ086977 |
| 0.28685 | mttB | Cenchrus fungigraminus vs PP503006 | NA | nad3 | Cenchrus fungigraminus vs PP503006 |
| 0.621707 | nad1 | Cenchrus fungigraminus vs AP008982 | 0.087406 | nad4 | Cenchrus fungigraminus vs AP008982 |
| NA | nad1 | Cenchrus fungigraminus vs AP012527 | 0.241041 | nad4 | Cenchrus fungigraminus vs AP012527 |
| 1.06067 | nad1 | Cenchrus fungigraminus vs AP013106 | 0.101835 | nad4 | Cenchrus fungigraminus vs AP013106 |
| 0.621707 | nad1 | Cenchrus fungigraminus vs AP013107 | 0.101835 | nad4 | Cenchrus fungigraminus vs AP013107 |
| 0.384847 | nad1 | Cenchrus fungigraminus vs BA000029 | 0.19377 | nad4 | Cenchrus fungigraminus vs BA000029 |
| 0.489922 | nad1 | Cenchrus fungigraminus vs DQ645537 | 0.61853 | nad4 | Cenchrus fungigraminus vs DQ645537 |
| 0.489922 | nad1 | Cenchrus fungigraminus vs DQ645538 | 0.61853 | nad4 | Cenchrus fungigraminus vs DQ645538 |
| 0.489922 | nad1 | Cenchrus fungigraminus vs DQ645539 | 0.61853 | nad4 | Cenchrus fungigraminus vs DQ645539 |
| 0.490844 | nad1 | Cenchrus fungigraminus vs DQ984518 | 0.488101 | nad4 | Cenchrus fungigraminus vs DQ984518 |
| 0.670973 | nad1 | Cenchrus fungigraminus vs EU365401 | 0.262756 | nad4 | Cenchrus fungigraminus vs EU365401 |
| 0.384847 | nad1 | Cenchrus fungigraminus vs JF281153 | 0.241041 | nad4 | Cenchrus fungigraminus vs JF281153 |
| 0.485542 | nad1 | Cenchrus fungigraminus vs JX999996 | 0.0972304 | nad4 | Cenchrus fungigraminus vs JX999996 |
| NA | nad1 | Cenchrus fungigraminus vs KJ078648 | 0.151172 | nad4 | Cenchrus fungigraminus vs KJ078648 |
| NA | nad1 | Cenchrus fungigraminus vs KU176938 | 0.52914 | nad4 | Cenchrus fungigraminus vs KU176938 |
| NA | nad1 | Cenchrus fungigraminus vs LC107874 | 0.488101 | nad4 | Cenchrus fungigraminus vs LC107874 |
| 0.71271 | nad1 | Cenchrus fungigraminus vs MF616338 | 0.231236 | nad4 | Cenchrus fungigraminus vs MF616338 |
| 0.277996 | nad1 | Cenchrus fungigraminus vs MG429050 | 0.192915 | nad4 | Cenchrus fungigraminus vs MG429050 |
| 0.743408 | nad1 | Cenchrus fungigraminus vs MN127968 | 0.151172 | nad4 | Cenchrus fungigraminus vs MN127968 |
| 0.742344 | nad1 | Cenchrus fungigraminus vs MN127974 |  |  |  |
| 1.02508 | nad1 | Cenchrus fungigraminus vs MZ506736 |  |  |  |
| 0.489922 | nad1 | Cenchrus fungigraminus vs NC_007982 |  |  |  |
| 0.732788 | nad1 | Cenchrus fungigraminus vs NC_008362 |  |  |  |
| 0.723919 | nad1 | Cenchrus fungigraminus vs NC_056367 |  |  |  |
| 0.743408 | nad1 | Cenchrus fungigraminus vs OK120846 |  |  |  |
| 0.622445 | nad1 | Cenchrus fungigraminus vs OQ086977 |  |  |  |
| 0.692256 | nad1 | Cenchrus fungigraminus vs PP503006 |  |  |  |

**Supplementary table 12. Pi value analysis in Poaceae**

| #No. | Region | Pi | Total Number of mutations | Region length |
| --- | --- | --- | --- | --- |
| 1 | *atp1* | 0.01248 | 69 | 1544 |
| 2 | *atp4* | 0.02028 | 47 | 684 |
| 3 | *atp6* | 0.03403 | 76 | 1586 |
| 4 | *atp8* | 0.02416 | 54 | 1185 |
| 5 | *atp9* | 0.03668 | 38 | 279 |
| 6 | *ccmB* | 0.00656 | 35 | 652 |
| 7 | *ccmC* | 0.00245 | 11 | 723 |
| 8 | *ccmFc* | 0.00846 | 48 | 2439 |
| 9 | *ccmFn* | 0.00718 | 52 | 1889 |
| 10 | *cob* | 0.00655 | 27 | 1563 |
| 11 | *cox1* | 0.0115 | 60 | 2235 |
| 12 | *cox2* | 0.00818 | 25 | 783 |
| 13 | *cox3* | 0.00329 | 18 | 843 |
| 14 | *matR* | 0.00737 | 58 | 2037 |
| 15 | *mttB* | 0.00739 | 9 | 823 |
| 16 | *nad1* | 0.00686 | 21 | 988 |
| 17 | *nad2* |  |  |  |
| 18 | *nad3* | 0.00471 | 6 | 468 |
| 19 | *nad4* | 0.00482 | 27 | 1488 |
| 20 | *nad4L* | 0.00357 | 5 | 324 |
| 21 | *nad5* | 0.00399 | 29 | 2013 |
| 22 | *nad6* | 0.01808 | 46 | 1360 |
| 23 | *nad7* | 0.00239 | 15 | 1206 |
| 24 | *nad9* | 0.00557 | 9 | 864 |
| 25 | *rpl16* | 0.01211 | 21 | 558 |
| 26 | *rps1* | 0.03145 | 46 | 677 |
| 27 | *rps12* | 0.00748 | 10 | 378 |
| 28 | *rps13* | 0.00759 | 10 | 351 |
| 29 | *rps2* | 0.09525 | 174 | 2481 |
| 30 | *rps3* | 0.01735 | 109 | 1716 |
| 31 | *rps4* | 0.02803 | 89 | 1452 |
| 32 | *rps7* | 0.00304 | 7 | 447 |
| 33 | *rrn18* | 0.00212 | 15 | 1997 |
| 34 | *rrn26* | 0.0032 | 31 | 3602 |
| 35 | *rrn5* | 0.00875 | 3 | 128 |

**Supplementary table 13. Homologous analysis in Poaceae**

| #ref | ref len | ref homo len | prop in ref | query | query len | query homo len | prop in query |
| --- | --- | --- | --- | --- | --- | --- | --- |
| *Zea_perennis* | 570354 | 539307 | 94.56% | *Zea_luxurians* | 539368 | 515507 | 95.58% |
| *Zea_luxurians* | 539368 | 480937 | 89.17% | *Zea_mays_subsp._mays* | 569630 | 516572 | 90.69% |
| *Zea_mays_subsp._mays* | 569630 | 560924 | 98.47% | *Zea_mays_subsp._parviglumis* | 680603 | 653188 | 95.97% |
| *Zea_mays_subsp._parviglumis* | 680603 | 491220 | 72.17% | *Tripsacum_dactyloides* | 704100 | 464856 | 66.02% |
| *Tripsacum_dactyloides* | 704100 | 291444 | 41.39% | *Sorghum_bicolor_subsp._drummondii* | 449028 | 250415 | 55.77% |
| *Sorghum_bicolor_subsp._drummondii* | 449028 | 441021 | 98.22% | *Sorghum_bicolor* | 468628 | 463203 | 98.84% |
| *Sorghum_bicolor* | 468628 | 305194 | 65.13% | *Chrysopogon_zizanioides* | 551622 | 306372 | 55.54% |
| *Chrysopogon_zizanioides* | 551622 | 337056 | 61.10% | *Saccharum_officinarum* | 445482 | 313980 | 70.48% |
| *Saccharum_officinarum* | 445482 | 194834 | 43.74% | *Cenchrus_macrourus* | 404398 | 194519 | 48.10% |
| *Cenchrus_macrourus* | 404398 | 217258 | 53.72% | *Eleusine_indica* | 520691 | 221244 | 42.49% |
| *Eleusine_indica* | 520691 | 203487 | 39.08% | *Oryza_sativa_Japonica_Group* | 490520 | 254400 | 51.86% |
| *Oryza_sativa_Japonica_Group* | 490520 | 451211 | 91.99% | *Oryza_sativa_Indica_Group* | 637692 | 617429 | 96.82% |
| *Oryza_sativa_Indica_Group* | 637692 | 560835 | 87.95% | *Oryza_minuta* | 515022 | 489060 | 94.96% |
| *Oryza_minuta* | 515022 | 483479 | 93.88% | *Oryza_rufipogon* | 525913 | 424500 | 80.72% |
| *Oryza_rufipogon* | 525913 | 378686 | 72.01% | *Oryza_coarctata* | 491065 | 317904 | 64.74% |
| *Oryza_coarctata* | 491065 | 255713 | 52.07% | *Bambusa_oldhamii* | 509941 | 219592 | 43.06% |
| *Bambusa_oldhamii* | 509941 | 219931 | 43.13% | *Lolium_perenne* | 678580 | 258484 | 38.09% |
| *Lolium_perenne* | 678580 | 312122 | 46.00% | *Hordeum_vulgare_subsp._spontaneum* | 525605 | 304878 | 58.01% |
| *Hordeum_vulgare_subsp._spontaneum* | 525605 | 525605 | 100.00% | *Hordeum_vulgare_subsp._vulgare* | 525599 | 525599 | 100.00% |
| *Hordeum_vulgare_subsp._vulgare* | 525599 | 317081 | 60.33% | *Elymus_magellanicus* | 583450 | 311028 | 53.31% |
| *Elymus_magellanicus* | 583450 | 303904 | 52.09% | *Agropyron_cristatum* | 381065 | 280152 | 73.52% |
| *Agropyron_cristatum* | 381065 | 309631 | 81.25% | *Thinopyrum_obtusiflorum* | 390725 | 305199 | 78.11% |
| *Thinopyrum_obtusiflorum* | 390725 | 324111 | 82.95% | *Aegilops_longissima* | 399005 | 317687 | 79.62% |
| *Aegilops_longissima* | 399005 | 359579 | 90.12% | *Triticum_timopheevii* | 443419 | 375582 | 84.70% |
| *Triticum_timopheevii* | 443419 | 410715 | 92.62% | *Triticum_aestivum* | 452528 | 420505 | 92.92% |
| *Triticum_aestivum* | 452528 | 424783 | 93.87% | *Aegilops_speltoides_var._ligustica* | 476091 | 429937 | 90.31% |
