# Supplementary figures and images for: The complete mitochondrial genome of Cenchrus fungigraminus indicates structural dynamics and sequence divergences in Poaceae family
Source: Front Plant Sci. 2025 May 30;16:1589847. doi: 10.3389/fpls.2025.1589847 (PMC12162664; doi:10.3389/fpls.2025.1589847)

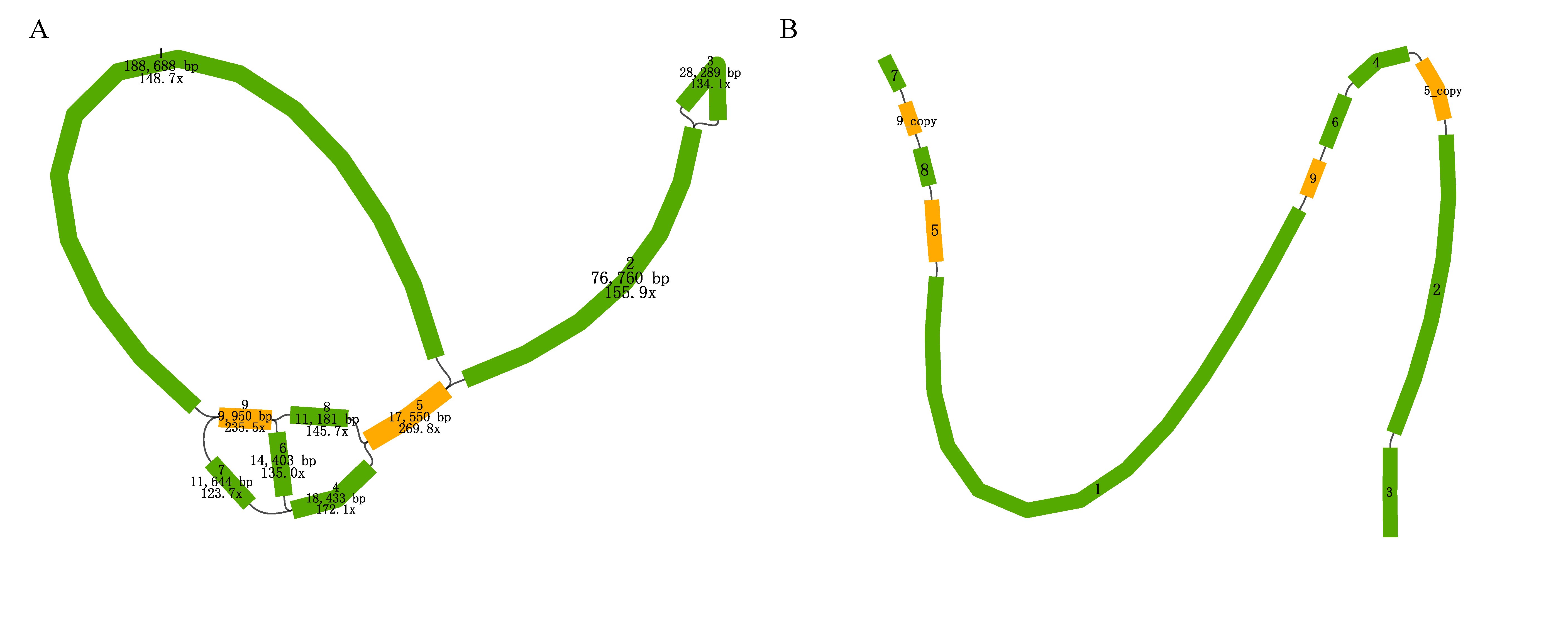

Supplement: Supplementary file 2 [file Image1.jpeg]

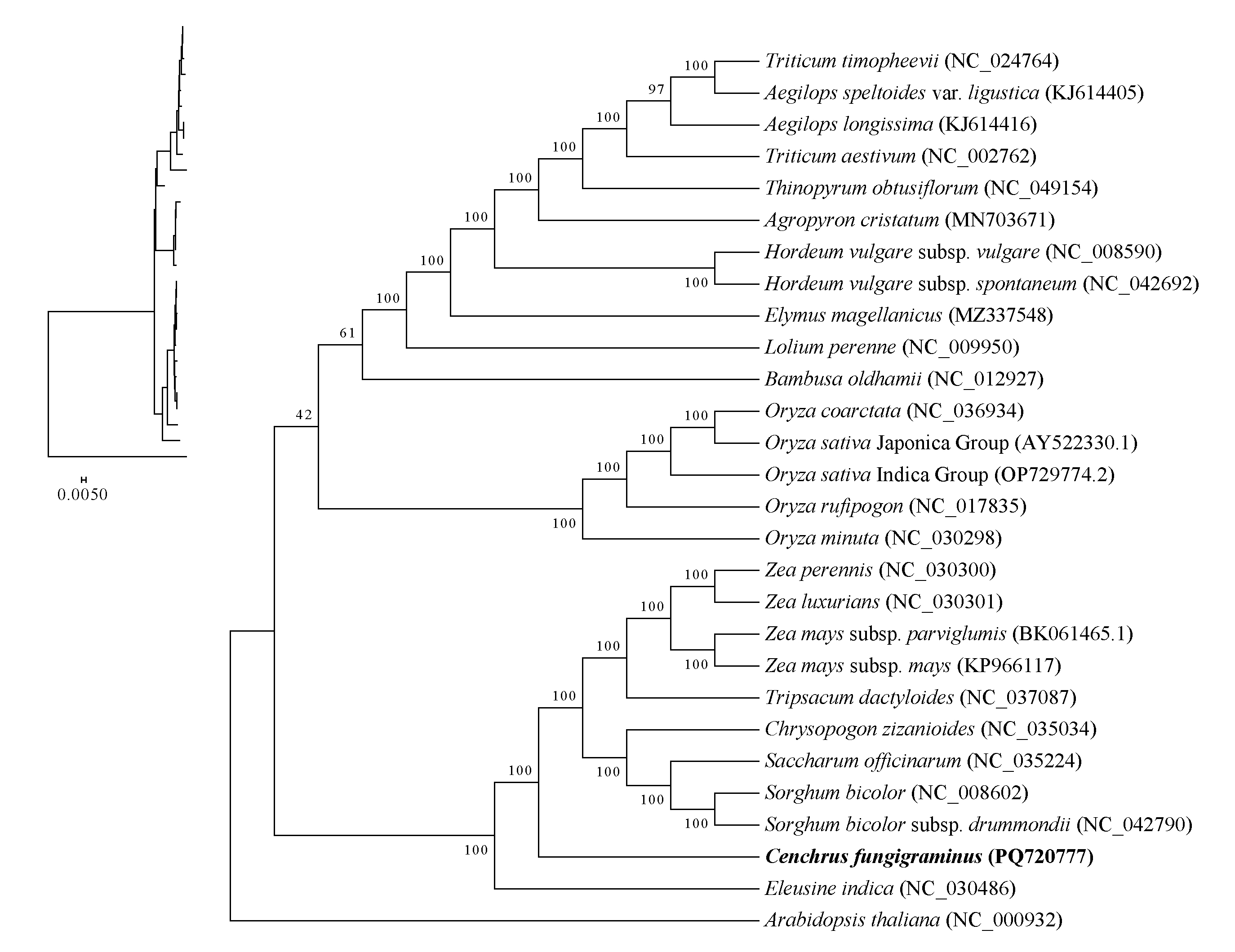

Supplement: Supplementary file 3 [file Image2.jpeg]

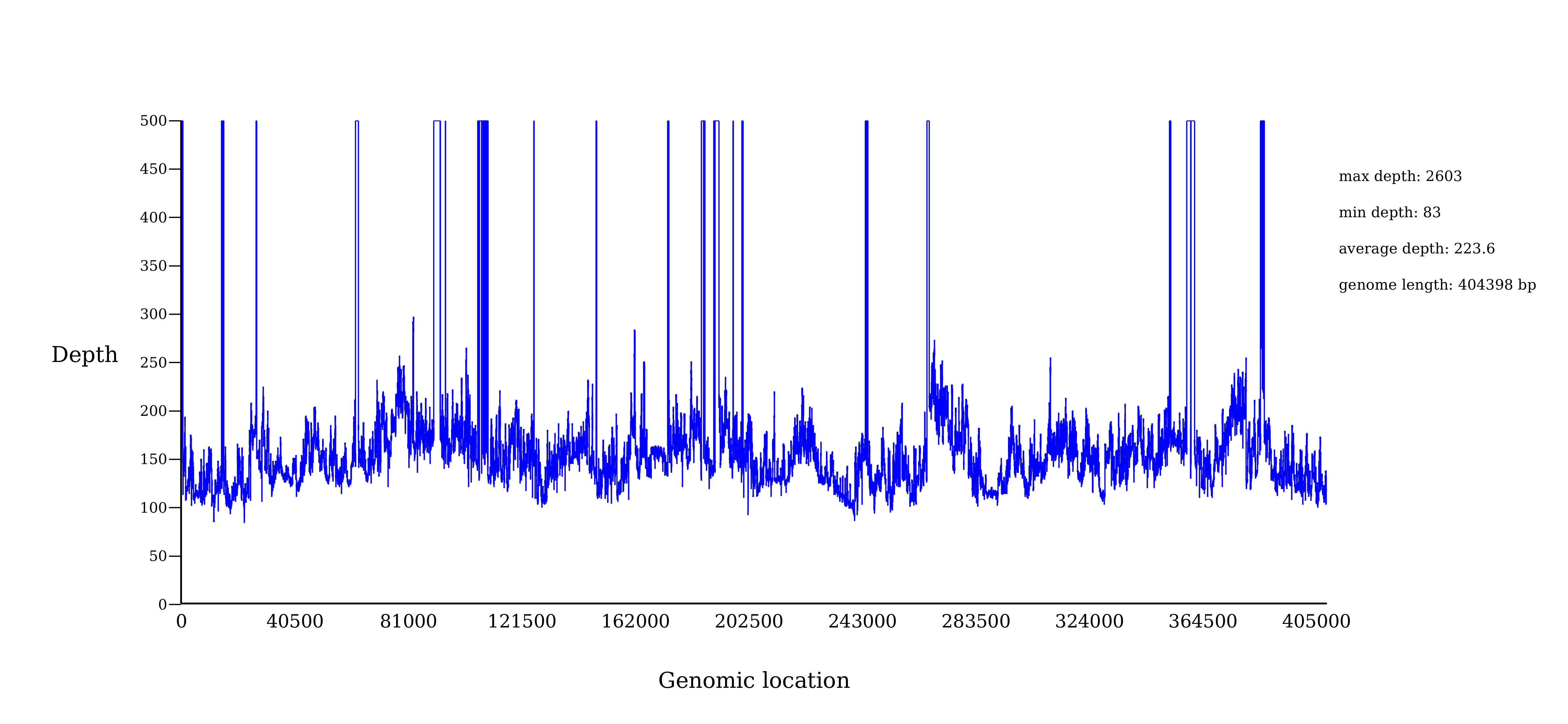

Supplement: Supplementary file 5 [file Image4.jpeg]
